# Computer-aided discovery of dual-target compounds for Alzheimer’s from ayurvedic medicinal plants

**DOI:** 10.1371/journal.pone.0325441

**Published:** 2025-06-25

**Authors:** Mohibullah Shah, Sadia Pervaiz, Iqra Ahmad, Shahid Habib Ansari, Valdir Ferreira De Paula Junior, Sibtain Ahmed, Muhammed Umer Khan, Khaled Fahmi Fawy, Umar Nishan, Hanna Dib, Mohamed A.O. Abdelfattah

**Affiliations:** 1 Department of Biochemistry, Bahauddin Zakariya University, Multan, Pakistan; 2 Department of Animal Science, Federal University of Ceara, Fortaleza, Brazil; 3 Faculty of Veterinary Medicine, Postgraduate Program in Veterinary Sciences, State University of Ceara, Fortaleza, Brazil; 4 Institute of Molecular Biology and Biotechnology, The University of Lahore, Lahore, Pakistan; 5 Chemistry Department, Faculty of Science, King Khalid University, Abha, Saudi Arabia; 6 Research Center for Advanced Materials Science (RCAMS), King Khalid University, AlQura’a, Abha, Saudi Arabia; 7 Department of Chemistry, Kohat University of Science & Technology, Kohat, Pakistan; 8 College of Engineering and Technology, American University of the Middle East, Kuwait; Guru Nanak College, INDIA

## Abstract

Alzheimer’s disease (AD) is a neurodegenerative disorder characterized by cognitive decline, driven by the accumulation of amyloid-beta plaques and neurofibrillary tangles. It involves the dysfunction of key enzymes such as Acetylcholinesterase (AChE) and β*-*secretase (BACE1), making them critical targets for therapeutic intervention. In this study we investigated an in-house library of 820 secondary metabolites obtained from Ayurvedic plants against AChE and BACE1 with the aim to discover novel leads for AD. Virtual screening resulted in 15 ligands, mostly belonging to the ursane-type or dammarene-type triterpene saponins of *Centella asiatica,* reestablishing the potency of this plant in drug discovery against AD. The binding affinities were further verified by molecular dynamics (MD) simulation trajectories, including root mean square fluctuations (RMSF), root mean square deviation (RMSD), hydrogen bonding analysis, Coulomb interaction calculation, Lennard-Jones interactions, and the total interaction energy. Moreover, extensive Principal Component Analysis (PCA) and Gibbs free energy landscape were performed. Our results demonstrated three compounds, namely (S)-eriodictyol 7-O-(6-β-O-trans-p-coumaroyl)-β-d-glucopyranoside, sitoindoside-X and 1,5-di-o-caffeoyl quinic acid as more effective in treating AD due to their comparable drug-like properties. Drug-likeness, structural chemistry, pharmacophore, and ADMET (Absorption, Distribution, Metabolism, Excretion, and Toxicity) analysis support their potential for future drug development. To establish the effectiveness of these lead compounds against AD, additional experimental testing should be performed.

## Introduction

Alzheimer’s disease (AD), a multifactorial neurodegenerative disorder, accounts for 60–70% of total cases of dementia worldwide [1]. It is mainly characterized by amnesia, apraxia, aphasia, dysphagia, disorientation, insomnia, depression, and delusions [2]. The likelihood of getting Alzheimer’s disease rises substantially with age, with a prevalence rate of about 2–10% and 20–30% at 65 and 85 years, respectively [3]. It is principally delineated by the formation of senile plaques and neurofibrillary tangles in the brain hippocampus region [4]. FDA-approved drugs for AD mainly include donepezil, rivastigmine, galantamine, and tacrine (AChE inhibitors); memantine (N-methyl-D-aspartate (NMDA) antagonist); and aducanumab (amyloid-β aggregation inhibitor). These drugs commonly cause side effects such as gastrointestinal issues (e.g., nausea, vomiting, diarrhea) associated with AChE inhibitors, dizziness and headaches with memantine, and brain swelling or amyloid-related imaging abnormalities (ARIA) with aducanumab. They have serious side effects and provide symptomatic relief rather than a permanent cure [5].

**Fig 1:**
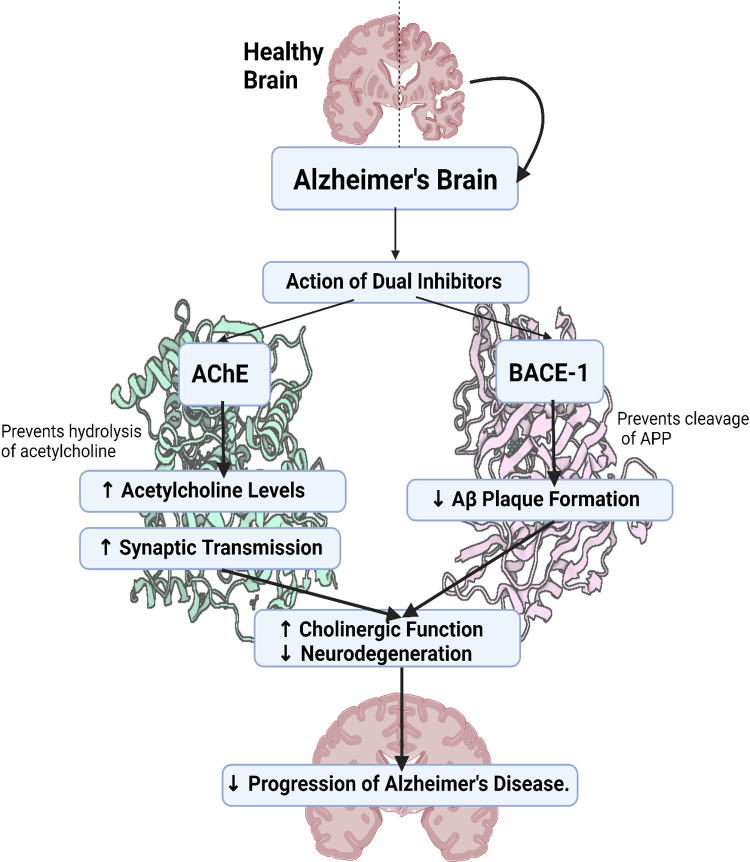
Diagram illustrating the effects of acetylcholinesterase (AChE) and β-secretase (BACE1) on signaling pathways, reducing the progression of Alzheimer’s disease.

The initial etiology of this disease is still unknown due to its complexity. Several hypotheses have been put forward regarding AD pathophysiology, most notably the cholinergic, β-amyloid, hyperphosphorylated tau, neuroinflammation, metal ion, and oxidative stress hypotheses, with the β-amyloid hypothesis being the most prominent [6]. This hypothesis gained support after the discovery of amyloid β-protein (Aβ) and its link to familial AD and trisomy 21 and has already led to the development of drugs that have entered clinical trials or have been on the market [7]. For instance, on June 7, 2021, the U.S. Food and Drug Administration (FDA) approved Aduhelm (aducanumab) for the treatment of Alzheimer’s disease [8]. Aβ’s formation and aggregation are crucial in this hypothesis. BACE1 is a key enzyme involved in the amyloidogenic pathway. It cleaves the amyloid precursor protein (APP) at the beta-site, leading to the production of soluble beta-amyloid (sAPPβ) and a membrane-bound fragment called C99. This fragment is then further processed by gamma-secretase to produce Aβ peptides [9] (Fig 1). The researchers speculate that using the BACE1 target can inhibit the deposition of Aβ and also delay tau protein pathological changes to a certain extent [10,11]. Another significant theory, the cholinergic hypothesis, posits that AD is partly caused by the loss of cholinergic neurons, which play a crucial role in memory and learning. In AD patients, the levels of acetylcholine are significantly reduced due to the degeneration of cholinergic neurons. Inhibiting AChE can prevent the degradation of acetylcholine, thereby increasing its concentration and improving cholinergic transmission [12]. Inhibiting acetylcholinesterase (AChE), with drugs like donepezil and rivastigmine has been proven effective [13].

Despite the success of single-target drugs, they overlook the complex interactions among different causes of a disease and do not account for the potential resistance of disease to treatment [14]. Therefore, the complex interactions of AD’s pathogenic factors prompt interest in dual-target drugs, aiming for improved efficacy by addressing multiple pathways simultaneously. Recent research focuses on combining AChE and BACE1 inhibitors, with compounds demonstrating strong inhibition activity and design approaches being explored. Maliszewski et al. for instance, produced several novel nitrogen mustard analogs by substituting a dipeptide residue for the 1,3,5-triazine ring and examined their inhibitory effects on both AChE and BACE1 by Ellman’s colorimetric and fluorescence resonance energy transfer methods, respectively [15]. Dhamodharan et al. used both linear and nonlinear techniques to create powerful inhibitors against AChE and BACE1 targets by utilizing a variety of machine-learning models [16]. Moreover, Wang et al. designed, synthesized, and biologically evaluated the furocoumarin scaffold of notopterol as a triple inhibitor of AChE/BACE1/GSK3β for the treatment of AD in mice [17]. This literature positions our work within the broader context of current research.

The significance of natural plants and their derivatives as a source of pharmaceuticals and diverse chemical structures has already been established [18–21]. Ayurvedic scriptures are replete with knowledge of Ayurvedic plants called Nervines that can aid in the improvement of memory and cognitive function in Alzheimer’s disease patients [22]. Phytoconstituent analysis of Ayurvedic plants revealed the presence of numerous beneficial substances, including alkaloids, flavonoids, terpenoids, lignans, tannins, phenols, and saponins, that exhibit high therapeutic potential against cognitive deterioration, amyloid-β aggregation, neuroinflammation, hypolipidemia, and oxidative stress [23]. Several in silico studies have explored potential inhibitors of these AChE and BACE1, often focusing on compounds from medicinal plants with established cognitive-enhancing properties. Flavonoids which have the widest source, are among the most notable dual inhibitors of AChE and BACE1. Coumarins, particularly the amidic nonpeptidic derivatives, have strong dual inhibitory ability, second only to flavonoids. Furthermore, terpenoids (Terreusterpenes D) and alkaloids (liensinine) have also exhibited favorable dual inhibitory activity for AChE and BACE 1 [24]. Previous research investigating Centella asiatica, have identified several compounds with dual-inhibitory activities. For instance, Marzouk Hagar Ali (2022) reported naringin and stigmasterol [25], Mawaddani (2020) focused on flavonol, germacrene B [26], and sitosterol and Khairinisa (2025) used multiple ligand mapping and MD simulations to reveal that asiaticoside and madecassoside from Centella asiatica exhibited strong binding affinities to AChE [27].

In our study, we explored the potential of a diverse and unique plant-based library, including Ayurvedic plants with therapeutic effects on the nervous system to develop dual inhibitors targeting AChE and BACE1, which are the key contributors to AD, leading to cognitive decline and memory loss. The rational basis for our approach is grounded in the complex pathology of AD, where simultaneously inhibiting both AChE and BACE1 could offer a more comprehensive treatment strategy than single-target drugs. Additionally, while dual-target strategies for AChE and BACE1 are common in AD research, no approved drugs currently target both enzymes simultaneously, which emphasizes the need for the study. Our approach is novel in that it combines both protein- and ligand-based drug design techniques, focusing on the active site, advanced molecular dynamics simulations, pharmacophore development, and pharmacokinetic profiling. We thereby performed *in silico* screening of 820 phytoconstituents from 10 selected Ayurvedic plants. Ethnopharmacological evidence supports the use of these plants in traditional medicine for treating memory loss, cognitive dysfunction, and other nervous system disorders. The binding affinities and interactions of these compounds were confirmed through MD simulation at 100 ns. We extended our investigation to include a comprehensive pharmacophore analysis, an examination of the structural chemistry of the top-performing compounds, and assessments of drug-likeness and biological properties. Additionally, we conducted ADME (Absorption, Distribution, Metabolism, and Excretion) and toxicity (T) analyses of the selected top dual inhibitors to find dual inhibitors of AChE and BACE1 for developing lead compounds with fewer side effects and more potency against Alzheimer’s disease.

## Materials and methods

### Retrieval and preparation of protein

**Fig 2:**
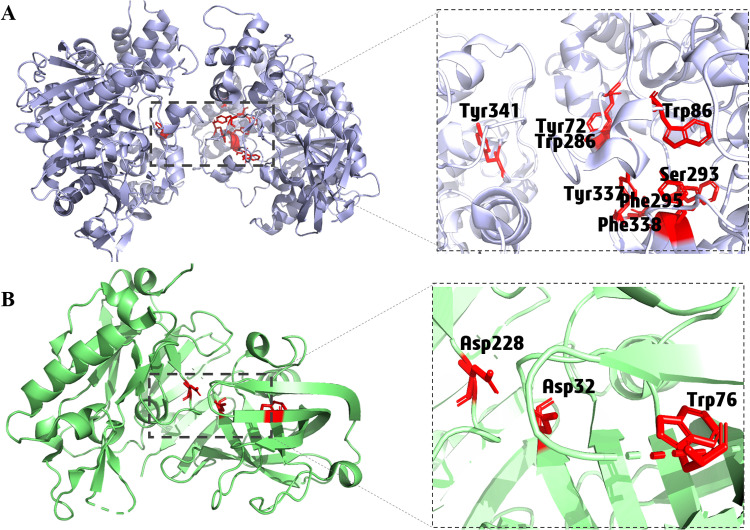
Structure of (A) human acetylcholinesterase (AChE, PDB ID: 6O4W) and (B) β-secretase (BACE1, PDB ID: 6EJ3); essential active site residues are highlighted in red.

The crystal structures of AChE and BACE1 were retrieved from the protein databank (https://www.rcsb.org) with PDB IDs of 6O4W [28] and 6EJ3 [29] respectively (Fig 2A and B). The Protein Data Bank is a publicly available repository that provides high-resolution structures of biological macromolecules. As performed in our recent studies [30,31], protein structures as receptors were prepared using the standard protocol for the MOE software, where all ligands and non-essential water molecules were removed. However, essential water molecules numbered 34, 88, 155, 157, and 260 were kept for AChE. The protonation and energy minimization of both proteins were carried out utilizing default MOE parameters.

### Phytoconstituents retrieval from Ayurvedic plants

Phytochemicals are biologically active nutrients widely reported to have desirable health benefits [32]. In this study, an in-house library of 820 secondary metabolites was prepared from ten Ayurvedic plants (S1 Table) [33–49] based on their potential to treat neurodegenerative disorders and the richness of their phytochemical diversity. A comprehensive literature survey was carried out to compile a comprehensive list of the phytoconstituents found in these plants. This rigorous approach was aimed at ensuring a thorough representation of their diverse phytochemical profiles in our database.

The selected Ayurvedic plants include *Bacopa monnieri* (L.) Pennell*, Nelumbo nucifera* Gaertn.*, Terminalia chebula* Retz.*, Acorus calamus* L.*, Withania somnifera* (L.) Dunal, *Emblica officinalis* Gaertn., *Coriandrum sativum* L., *Centella Asiatica* (L.) Urb., *Rauvolfia serpentina* Benth. ex Kurz, and *Punica granatum* L. [50].

*B. monnieri*, widely known as Brahmi, belonging to the family of Scrophulariaceae is a nootropic ayurvedic herb known to be effective in treating neurological disorders for more than 3000 years [33,48]. *N. nucifera* (indian lotus), locally known as Poddo, belongs to the family Nymphaeaceae, and has been conventionally used (for more than 2000 years) to treat various disorders such as nervous disorders, epilepsy, hypertensin and cardiovascular diseases [40,51]. *T. chebula*, known as the Haritaki plant, is a widely used medicinal plants in the Ayurveda. Its various has pharmacological uses include neuroprotective, cardioprotective, antidiabetic,and antimicrobial, properties [42,52]. *A. calamus*, commonly known as Vacha in Sanskrit, belongs to the family Acoraceae is a traditional Ayurvedic medicinal herb, which is practiced to treat a wide range of health ailments, including neurological disorders, for learning and memory-enhancing activity [47]. Simlarly, *W. somnifera* (known as Ashwagandha) [35,49], *E. officinalis* (known as Amla) [37], *C. sativum* (known as coriander and cilantro) [40,45,46], *C. Asiatica* (known as Gotu Kola) [26,43,53,54], *R. serpentina* (known as Sarpagandha) [39] and *P. granatum* [38,50] have also been extensively studied for their neuroprotective, antioxidant, and anti-inflammatory properties.

*A. calamus* yielded compounds 1–124, the majority of which were phenylpropanoids (75% α-asarone), alkaloids, sesquiterpenoids, monoterpenes, xanthine glycosides, saponins, and sterols [47]. *B. monnieri* (Comp. 125–186) had bacoside A as the main active phytoconstituent. Other secondary metabolites included alkaloids, flavonoids, triterpenoids, sesquiterpenoids (plantainoside B), triterpenoid saponins (bacosides I–XII), and sterols [48]. The bioactive compounds of *W. somnifera* (Ashwagandha) ranged from 187 to 248 and primarily comprised withanolides, alkaloids, sitoindosides, flavonoids, and steroidal lactones [35]. *T. chebula* (comp. 249–312) mainly included tannins (32%–34%), monoterpenes, triterpenoids, flavonoids, and phenolic carboxylic compounds [52]. The perennial herb *C. asiatica* (comp. 313–378) mainly yielded triterpene pentacyclic saponins (asiaticoside and asiatic acid), flavonoids, terpenoids, and many other polyphenols [36]. *E. officinalis* (Amla)-derived compounds (379–431) exhibited their ayurvedic potential due to the content of hydrolyzable tannins, flavonoids, phenolic compounds, flavanone glycosides, alkaloids, and vitamin C (ascorbic acid) [37]. Compounds 432–522 were acquired from *N. nucifera* (Indian Lotus) and included triterpenoids, megastigmane/sesquiterpene compounds, flavonoids, phenolic compounds, alkaloids, and sterols [41]. The phytochemicals of *P. granatum*. (pomegranate) ranged from 523–600 and included alkaloids, polyphenols (predominantly tannins and punicagalins), triterpenoids, and flavonoids [38]. *R. serpentina.* (Sarpagandhi) main phytoconstituents were indole alkaloids, flavonoids, tannins, saponins, and phenols (comp. 601–694) [39]. *C. sativum* (comp. 695–820), commonly called Dhania or Chinese parsley, contained secondary metabolites that included terpenoids, alkaloids, flavonoids, polyphenols, glycosides, sterols, tocols, and tannins [40].

### Ligand preparation

The names and structures of 820 phytoconstituents from ten Ayurvedic plants, as well as those of the standard inhibitors donepezil and elenbecestat (S1 Table), were obtained from the literature survey. They were searched and retrieved from the PubChem database (http://www.pubchem.ncbi.nlm.nih.gov) in.mol format, a free chemical database that provides detailed information on the properties and biological activities of small molecules. For compounds not available in PubChem, we manually sketched them using ChemDraw 12.0 based on reported structures. An *in-house* database was created in the molecular operating environment (MOE). The addition of partial charges and energy minimizations was carried out using default parameters (using the MMFF94x force field) to standardize the dataset and increase the stability of ligands [30].

### Active site determination

The active site was determined by using the SiteFinder application of MOE software, which identifies potential binding pockets based on geometric and physicochemical properties.The binding pocket was chosen considering the position of the co-crystallized ligands, their larger size, and the already reported amino acid residues for AChE [28] and BACE1 [55]. Dummy atoms were built at alpha-sphere centers to serve as the active site.

### Pose validation

The co-crystallized ligands donepezil and B7T were re-docked into AChE and BACE1, respectively. PyMol software, a molecular visualization tool, was used to calculate the root mean square deviation (RMSD) value [30,56]. This was achieved by superimposing re-docked poses of ligands to their respective native confirmations in the AChE and BACE1 crystal structures. A RMSD value less than 2 Å will validate the ability of the docking algorithm to regenerate the native pose [57].

### Molecular docking

The docking of the energy-minimized database of 820 compounds was carried out using the default parameters of Molecular Operating Environment (MOE), 2022.02 Chemical Computing Group ULC, 1010 Sherbooke St. West, Suite #910, Montreal, QC, Canada, H3A 2R7, MOE2022.v11.18.1), separately against prepared structures of human AChE and β*-secretase* (BACE1) in MOE software. The induced fit model was enabled to allow for flexible target binding to ligands [58]. The docking process was divided into multiple stages to guarantee the best possible contact between the ligands and the target proteins. The Triangle Matcher Algorithm was initially used to position the ligand. During the first rescoring step, London dG was used with a maintain value set to 10. After the first score, ligand placement within the active sites was adjusted using the forcefield approach as part of a refining process. The GBVI/WSA dG was used once more in the second rescoring phase, this time with a retain value of 5. With this method, it was made sure that the final analysis only took into account the ligand positions that were most energetically beneficial. Duplicates were eliminated throughout the process. Protein-ligand interactions and their binding affinities were predicted by flexible docking. The highest-scoring compounds were selected for further downstream analysis. The ligand scores were compared to those of the standard inhibitors, donepezil, and elenbecestat [59].

### Pharmacophore study

After thoroughly examining ligand interactions among the top compounds, the best compound was selected based on its position according to the docking score and its optimal molecular weight (near 500) compared to the other top compounds. The pharmacophoric features were generated for the binding confirmations of compound 6, namely S-eriodictyol7-O-(6-β-O-trans-p-coumaroyl)-β-d-glucopyranoside, with both AChE and BACE binding sites. A high-quality pharmacophore model was created with the Pharmacophore Query Editor in MOE that generated a variety of predefined pharmacophore features, including hydrogen bond donors (Don), hydrogen bond acceptors (Acc), aromatic centers (Aro), Pi ring centers (PiR), aromatic rings or Pi rings normal (PiN), hydrophobic atoms (Hyd), anionic atoms (Ani), and cationic atoms (Cat), among others. The radius of the detected pharmacophore features was calibrated to 1.0 Å with a tolerance value of 1.2 and a threshold value of 50%. The distances between the features were also measured [60].

### Molecular dynamic simulation

In order to examine the binding stability, conformation, and interaction processes of the chosen bioactive compounds (ligands) and receptors, two of the top ligands were subjected to molecular dynamic simulation. The AChE co-crystals were retrieved from the Protein Data Bank (PDB) with PDB ID: 6O4W [28] and BACE1, co-crystals with PDB ID: 6EJ3 [29]. Both were subjected to preparation by editing the missing residues using SwissPDBViewer [61], followed by protonation adjustment using the APBS (Adaptive Poisson-Boltzmann Solver) web service [62]. Subsequently, the inhibitors co-crystalized compound Donepezil/AChE, and CP23/BACE1 were retrieved from each complex using PyMOL software [63] to obtain the regions of interest for the compounds. GROMACS is a widely used software package for simulating biomolecular interactions. GROMACS v2024 [64] atomistic simulations were conducted to analyze the structural changes of the complex formed by AChE and BACE1 with natural inhibitors, utilizing the CHARMM36 force field. Subsequently, a “cubic” type box with edges of 2 nm was created for solvating the system with water molecules parameterized using TIP3P [65]. Additionally, Na^+^ ions were employed to neutralize the system. Electrostatic interactions were simulated using the fast particle-mesh Ewald approach, while van der Waals interactions (vdW) were calculated using the cutoff scheme. Initially, the complex underwent energy minimization using the steepest descent approach. Temperature equilibration of the system was achieved at 310 K using the NVT ensemble, and pressure equilibration was attained using Berendsen coupling with a compressibility of 4.5 × 10−5 bar in the NPT ensemble. Molecular dynamics (MD) simulations were conducted based on the adapted protocols already published by de Paula Junior *et al*. [66]. In summary, the MD was simulated on a 100 ns scale using an accelerated hybrid CPU-GPU Python architecture. The stability of the systems was evaluated using root mean square deviation (RMSD), root mean square fluctuation (RMSF), hydrogen bond interaction mapping analysis, Coulomb interaction calculation, Lennard-Jones interactions, Gibbs free energy landscapes, and principal component analysis (PCA) was performed to predict correlated motions generated during protein-ligand and the total interaction energy of the system [66].

### Drug-likeness analysis

The Molinspiration online server was used to perform QSAR studies to evaluate the physiochemical, structural, and pharmacokinetic properties of lead compounds along with their bioactivity score. These properties were compared to those of the standard inhibitors donepezil and elenbecestat to check their therapeutic potential. Additionally, DruLiTo software assesses the ligand’s propensity to abide by drug-likeness rules. These rules included the Lipinski rule, the Ghose filter, the Veber filter, MDL Drug Data Report (MDDR)-like rules, blood-brain barrier (BBB) permeability, the CMC-like rule, and the quantitative estimate of drug-likeness (QED). Lipinski’s rule of five is of utmost importance, suggesting a molecular weight of ≤ 500 Da, Log P ≤ 5, H bond acceptors ≤ 10, and H bond donors ≤ 5 for a compound to be a potential drug candidate. While BBB permeability needs a molecular weight ≤ 400–500 Da and 8–10 hydrogen bonds. MDDR rule shows for a compound to be drug-like No. of rotatable bonds should be ≥ 6, no. rings ≥ 3, and no. of rigid bonds ≥ 18. Veber’s filter describes better oral bioavailability as requiring a polar surface area ≤ 140 and a number of rotatable bonds ≤ 10 [67].

### ADMET analysis

ADMET (Absorption, Distribution, Metabolism, Excretion, and Toxicity) profiles of top compounds were predicted using ADMET Lab 2, an online platform that evaluates absorption, distribution, metabolism, excretion, and toxicity characteristics of compounds [68]. Later they were compared with standards donepezil and elenbecestat. The predicted pharmacokinetic features mainly included absorption, distribution, metabolism, excretion, and toxicity.

## Results and discussions

### Active site determination

The MOE indicated two active sites, one having 163 amino acid residues and the other having 175 amino acids, were selected based on previously reported essential amino acid residues for human AChE and BACE1, respectively (Fig 2A and B). Active site 1 contained all the essential amino acid residues, including Ser293, Phe295, Tyr72, Tyr341, Trp86, Trp286, Tyr337, and Phe338, required for the binding of the co-crystallized ligand donepezil to AChE [28]. BACE1 active site 2 was defined by the presence of Asp32, Asp228, and Trp76, essential for hydrogen bonding of the co-crystallized ligand B7T to BACE1 [55].

### Binding interactions analysis by molecular docking

The *in-house* library, comprising 820 secondary metabolites retrieved from ten Ayurvedic medicinal plants (S1 Table), in addition to the standard inhibitors donepezil and elenbecestat, was docked against the target proteins AChE and BACE1, respectively. The molecular docking analysis revealed that 773 out of 820 phyto-constituents showed good interactions, while 47 compounds were found inactive, displaying no interactions in comparison to standard inhibitors (S1 Table). Molecular docking extrapolates different intermolecular interactions, including hydrogen bonds, hydrophobic interactions, H-Pi bonds, and π-stacking. The nature and type of these interactions play a significant role in making protein-ligand complexes stable for carrying out various biological and physiological functions. These include cellular modulation, enzyme inhibition or activation, gene regulation, and signal transduction [69].

The molecular docking revealed 15 compounds as dual inhibitors against AChE and BACE1. They exhibited remarkably more negative scoring functions as compared to the standard inhibitors donepezil and elenbecestat (Table 1). The lower (more negative) scores reflect a more stable and favorable interaction in the docking analysis. It suggests that our ligands may be more effective in inhibiting the targets compared to the standard inhibitors donepezil and elenbecestat. These compounds were further subjected to downstream analysis. The majority of these dual inhibitors belonged predominantly to the class of triterpenes, which have six isoprene units. *C. asiatica* was the source of 12 out of these 15 secondary metabolites, showing the high potential of this plant for treating neurological issues. Our findings align with previous *in silico* studies. For instance, Mawaddani et al. [26] demonstrated the potential of *C. asiatica* flavonoids, such as sitosterol and flavonol, to inhibit BACE1 through molecular docking, with the binding energies of −239.7 kcal/mol and −188.1 kcal/mol, respectively, which are comparable to the compounds identified in our study. Furthermore, Jusril et al. [70] confirmed that triterpenes like asiatic acid and madecassic acid, both detected in *C. asiatica*, exhibited strong AChE inhibitory activity through *in silico* docking studies, further supporting the inhibitory properties observed in our lead compounds. *C. asiatica* (Common name: Indian pennywort, Gotu Kola), belonging to the family Apiaceae, has an established role as a rejuvenating herb. It had been reported to have an IC_50_ value of 270 ± 18.9 µM against a positive control for the AChE inhibition assay [57].

**Table 1:** Structures, class, and docking scores of top fifteen ayurvedic compounds against acetylcholinesterase (AChE) and β-secretase (BACE1).

Sr. No.	Name	Plant	Class of compound	AChE score (kcal/mol)	BACE1 score (kcal/mol)
S1	Donepezil		Piperidine derivative	−10.34	–
S2	Elenbecestat		Synthetic organic	–	−6.22
1	Tannic acid	*Nelumbo nucifera*	Aromatic phenolic compound (Tannin)	−13.19	−13.31
2	Centellasaponin A	*Centella asiatica*	Triterpenes	−12.07	−10.31
3	Centelloside A	*Centella asiatica*	Triterpenes	−11.77	−9.68
4	23-O-acetyl asiaticoside B	*Centella asiatica*	Triterpenes	−11.12	−9.99
5	Scheffoleoside A	*Centella asiatica*	Triterpenes	−10.73	−9.71
6	(S)-eriodictyol 7-O-(6-B-O-trans-p-coumaroyl)-B-dglucopyranoside	*Emblica officinalis*	acrylated flavanone glycosides	−10.72	−8.68
7	Asiaticoside C	*Centella asiatica*	Triterpenes	−10.69	−9.93
8	Sitoindoside-X	*Withania somnifera*	withanolide saponin	−10.61	−9.69
9	Asiaticoside G	*Centella asiatica*	Triterpenes	−10.54	−9.10
10	Asiaticoside I	*Centella asiatica*	Triterpenes	−10.54	−9.73
11	Centellasaponin H	*Centella asiatica*	Triterpenes	−10.50	−10.17
12	Asiaticoside	*Centella asiatica and Bacopa monnieri*	Triterpenes	−10.49	−8.66
13	centelloside F	*Centella asiatica*	Triterpenes	−10.49	−9.15
14	Centellasaponin I	*Centella asiatica*	Triterpenes	−10.48	−9.43
15	1,5-di-o-caffeoyl quinic acid	*Centella asiatica*	Phenylpropanoids	−10.37	−7.50

**Fig 3:**
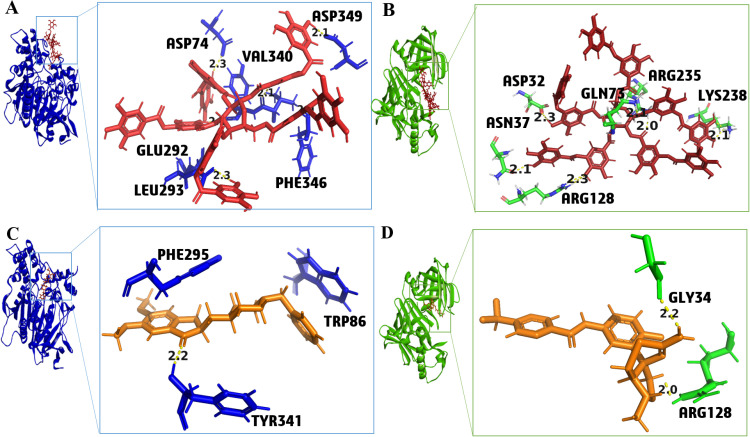
Ligand protein interaction of (A) Compound 1 (red) with acetylcholinesterase (AChE), (B) Compound 1 (red) with β-secretase (BACE1), (C) Standard donepezil (orange) with AChE, and (D) Standard elenbecestat (orange) with BACE1.

The compound 1, tannic acid, inhibited AChE and BACE1 with the highest docking scores of −13.19 and −13.31, respectively, as compared to the standard inhibitors donepezil (−10.34) and elenbecestat (−6.22) (Table 1). It formed seven hydrogen bonds with AChE involving Tyr34, Asp74, Leu293, Phe346, Asp349, Val340, and Glu292 residues (Figs 3A and S1A). Tannin also made six H-bonds with BACE1 involving Asp32, Asn37, Arg235, Gln73, Arg128, and Lys238 residues (Fig 3B and S1B) Two critical amino acids were involved during binding to the compound 1 namely Tyr341 for AChE and Asp32 for BACE1. However, the standard donepezil showed interactions with Phe295 (H-bond), Tyr341 (H-pi), and Trp86 (π- π stacking) (Fig 3C and S2A) and the standard elenbesectat formed two H-bonds with Gly34 and Arg128 against BACE1 (Fig 3D and S2B). The 2D ligand interaction diagrams of compound 1 and both the standards, with AChE and BACE1 are presented in S1 and S2 Figs. It is a tannin derived from the flowers of *N. nucifera*, showing an IC_50_ value of 76 ± 9.2 µg/ml against AChE [46]. However, the relatively high IC50 suggests that a higher concentration of tannic acid is required to achieve significant inhibition of AChE. These results may indicate a limited pharmacological applicability unless optimized. Moreover, it also exhibited IC_50_ value of 115.05 nM against AChE, in another study [71]. The discrepancy in IC50 values from different studies highlights the variability in experimental conditions. It creates the need for further standardized studies to fully assess its inhibitory potential. *N. nucifera* (common name: Lotus, Kamala) belongs to the family Nelumbonaceae and has gained ample attention in Ayurveda for its anti-oxidant, anti-epileptic, anti-inflammatory, and anti-aging properties [51]. *In vivo* analysis of tannic acid also indicated a positive effect on memory loss, hyperactivation, object identification, and tau protein aggregation [72]. It also acts as a potent anti-oxidant, having a ki value of 50.96 ± 2.18µM for AChE [73].

**Fig 4:**
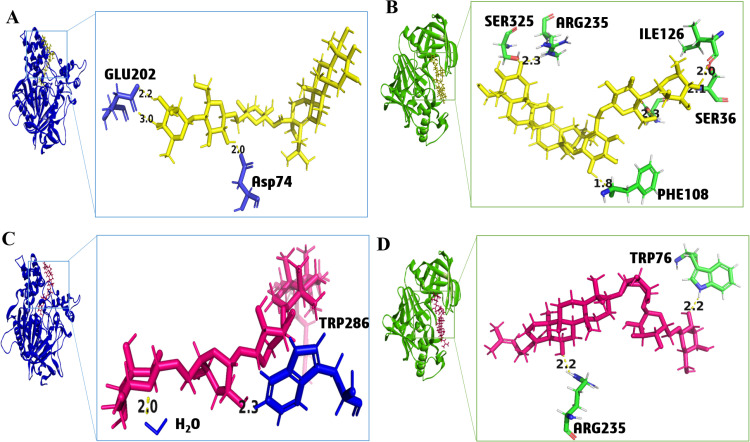
Ligand-protein interaction of (A) Compound 2 (Yellow) with acetylcholinesterase (AChE), (B) Compound 2 (Yellow) with β-secretase (BACE1), (C) Compound 3 (Pink) with AChE, and (D) Compound 3 (Pink) with BACE1.

The compound 2, centellasaponin A, showed docking scores of −12.07 and −10.31 against AChE and BACE1, respectively (Table 1). It is a triterpenoid obtained from *C. asiatica.* It made two H-bonds, with the Glu202 having a binding energy of −1.4 and −3.6 kcal/mol and one with Asp 74 (−1.2 kcal/mol) regarding AChE (Fig 4A and S3A). The binding energies of the compounds are presented in S2 Table. It made six H-bonds with BACE1 involving Arg235, Ser325, Asp32, Ile126, Phe108, and Ser36 residues (Fig 4B and S3B). The compound 3, centelloside A, was also derived from *C. asiatica*. It showed docking scores of −11.77 and −9.68 against AChE and BACE1, respectively (Table 1). It made one H-bond with HOH 875 (−1.4 kcal/mol) and two H-pi bonds with Trp286 (−0.6 kcal/mol) (Fig 4C and S3C). It also made two H-bonds with BACE1 involving Trp76 and Arg235 with binding energies of −1.7 kcal/mol and −1.5 kcal/mol, respectively (Fig 4D and S3D). The 2D interaction profiles of compounds 2 and 3 are presented in S3A-D Fig. *In vivo* studies on *C. asiatica* depicted a dose-dependent improvement in the antioxidant and cognitive abilities of the mice model [74]. *C. asiatica* is also used as a neuroprotectant for improving memory, cognitive decline, learning processes, epilepsy, and depression in Ayurvedic and traditional Chinese medicine [36].

The compound 4, 23-O-acetyl asiaticoside B, showed dual inhibition of AChE and BACE1 with docking scores of −11.12 and −9.99, respectively (Table 1). It made two H-bonds with AChE, including amino acid residues Asp74 (−2.2 kcal/mol) and Phe295 (−3.2 kcal/mol), and two H-pi bonds involving Phe338 (−1.0 kcal/mol) and Trp286 (−0.6 kcal/mol) (S4A and B Fig). It also made 4 hydrogen bonds with the BACE1 receptor, including Asp228, Lys224, Thr72, and Thr232 residues (S4C and D Fig). Asiaticosides are widely reported in the literature for preventing amyloid-β plaques, neuroinflammation, and neurofibrillary tangles (NFTs) [75]. The compound 5 was scheffoleoside A, which could inhibit both AChE and BACE1 with scores of −10.73 and −9.71, respectively. It made one hydrogen bond with Asp74 (−0.6 kcal/mol) and one H-pi bond with Tyr337 (−0.6 kcal/mol) in the case of AChE (S5A and B Fig). It also made two H-bonds with BACE1 involving the amino acids Gly230 (−0.7 kcal/mol) and Lys321 (−3.7 kcal/mol) (S5C and D Fig).

The compound 6, S-eriodictyol7-O-(6-β-O-trans-p-coumaroyl)-β-d-glucopyranoside, is a flavanone glycoside that inhibits AChE and BACE1 with binding scores of −10.72 and −8.68, respectively (Table 1). It inhibited AChE by making one hydrogen bond with Asp74 (3.0 kcal/mol). It also made one π-π and one H-pi interaction with Trp86 (−1.6 and −0.6 kcal/mol) and one with Trp286 (1.6 kcal/mol). Its aromatic group was involved in π-π interaction with the Trp 86 residue (S6A and B Fig). It could inhibit BACE1 by making two H-bonds with Asp228 (−3.1 and −4.1 kcal/mol) and one with Arg128 (−0.8 kcal/mol). It also made one H-pi bond, with Trp76 having −1.0 kcal/mol binding energy (S6C and D Fig). Three critical residues, namely Tyr286 and Trp86 for AChE and Asp228 for BACE1, were involved in the binding with compound 6. It was derived from *E. officinalis* (fruit), which was previously reported to have an IC_50_ = 7 ± 0.9 µg/ml for AChE against the positive control Physostigmine [54].

The compound 7, asiaticoside C, showed a binding score of −10.69 and −9.93 for AChE and BACE1, respectively (Table 1). It inhibited AChE by making two H-bonds with Ser293 (−2.1 kcal/mol) and one with Phe295 (−1.4 kcal/mol). It also showed an H-pi bond with Trp286 (−0.9 kcal/mol) (S7A and B Fig). It inhibited BACE1 by making nine H-bonds with Asn233, Thr72, Asp32, Lys321, Asp228, Lys321, Asp32, Thr72, and Arg128, having binding energies of −0.9, −0.6, −2.2, −4.2, −2.8, −4.2, −0.9, −0.6, and −1.0 kcal/mol, respectively (S7C and D Fig). Five critical residues, namely Tyr286, Ser293, and Phe295 for AChE and Asp228 and Asp32 for BACE1, were involved in the binding with compound 7. The compound 8, sitoindoside-X, is a withanolide saponin that demonstrated −10.61 and −9.69 docking scores for AChE and BACE1, respectively. It formed two H-bonds with Tyr341 (−0.8 kcal/mol) and HOH832 (−0.5 kcal/mol) and an H-pi bond with Tyr341 (−0.7 kcal/mol) against AChE (S8A and B Fig). It also inhibited BACE1 by making three H-bonds with Asn37 (−0.8 kcal/mol), Thr232 (−2.0 kcal/mol), and Asn233 (−1.6 kcal/mol) (S8C and D Fig). It was derived from *W. somnifera* (Ashwagandha) that previously showed an IC_50_ value of 850 ± 65 µg/ml against the physostigmine control (0.075 ± 0.003 µg/ml) for the AChE inhibition assay [50]. It also functions as a nootropic agent that enhances memory and cognitive abilities and revives the nervine tonic [49].

The compound 9, asiaticoside G, is a triterpenoid that presented high binding scores of −10.54 and −9.10 for AChE and BACE1, respectively (Table 1). It binds with AChE by making three H-bonds with Asp74 (−2.1 kcal/mol), Phe295 (−0.8 kcal/mol), and HOH788 (−0.7 kcal/mol) and three H-pi bonds with Tyr341 (−0.9 kcal/mol), Tyr337 (−0.9 kcal/mol), and Tyr337 (−0.7 kcal/mol) (S9A and B Fig). It also interacted with BACE1 by making three H-bonds with Arg307, Thr72, and Trp76 residues acting as hydrogen acceptors (S9C and D Fig). Four critical residues, namely Tyr341, Tyr337, and Phe295 for AChE and Trp76 for BACE1, were involved in binding with the compound 9. The compound 10, asiaticoside I, showed a docking score of −10.54 against AChE by making five hydrogen bonds with His447, Glu202, His287, HOH797, and HOH708, having −1.9, −1.6, −0.6, −0.6, and −0.8 kcal/mol energy, respectively (S10A and B Fig). It also presented a binding score of −9.73 against BACE1 by making two H-bonds with Asp228 (2.6 and −0.6 kcal/mol) and one with Asp32 (−3.0 kcal/mol) (S10C and D Fig).

In this study, the compound 11, centellasaponin H, presented a docking score of −10.50 against AChE by making three H-bonds with HOH711 (−1.3 kcal/mol), Ser293 (−1.3 kcal/mol), and Asp74 (0.3 kcal/mol) (S11A and B Fig). It also binds to the BACE1 active site by making two H-bonds with Leu263 (−1.1 kcal/mol) and Arg128 (−1.3 kcal/mol) (S11C and D Fig). The compound 12, asiaticoside, was derived from the plants *C. asiatica* and *B. monnieri*. It interacted with AChE by forming two H-bonds with Asp74 and HOH875 with −1.8 and −0.7 kcal/mol energies, respectively (S12A and B Fig). It also inhibited BACE1 by making two H-bonds with Asp228 (−3.6 and −2.2 kcal/mol) (S12C and D Fig). *In vivo* studies on asiaticoside have already reported its therapeutic potential for treating wounds and scars, memory loss, depression, stress, and anxiety [76]. The high potency of *B. monnieri* (common name: Brahmi, water hyssop) as a perennial nootropic herb for improving learning behavior, memory loss, depression, and cognitive dysfunction has also been established [77]. It showed an IC_50_ value of 523 ± 39.7 µg/ml against a positive control for the AChE inhibition assay [50].

Compound 13, centelloside F, showed docking scores of −10.49 and −9.15 against AChE and BACE1, respectively (Table 1). It interacted with AChE through two H-bonds with HOH756 (−0.4 kcal/mol) and Glu202 (−1.1 kcal/mol) and an H-pi bond with Tyr337 (−0.8 kcal/mol) (S13A and B Fig). It also inhibited BACE1 by making four hydrogen bonds with Phe108 (−2.3 kcal/mol), Arg307 (−1.3 kcal/mol), Lys224 (−2.9 kcal/mol), and Arg307 (−2.0 kcal/mol) (S13C and D Fig). The compound 14, centellasaponin I, is a triterpenoid that showed −10.48 and −9.43 scores against AChE and BACE1. It binds to AChE by five H-bonds, with Ser293, Phe295, Asp74, HOH771, and HOH875 having binding energies of −0.8, −1.2, −1.3, −1.4, and −0.2 kcal/mol, respectively (S14A and B Fig). It also inhibited BACE1 by making four H-bonds with residues Thr232 (−0.9 kcal/mol), Trp76 (−2.5 kcal/mol), and Arg128 (−0.8 and −4.0 kcal/mol) (S14C and D Fig).

The compound 15, 1,5-di-o-caffeoyl quinic acid, a phenylpropanoid, exhibited docking scores of −10.37 and −7.50 against AChE and BACE1, respectively (Table 1). It inhibited AChE by making two H-bonds with HOH756 (−0.7 kcal/mol) and Glu202 (−3.7 kcal/mol), a H-pi bond with Trp286 (−0.6 kcal/mol) and π-π stacking with Trp86 (−0.6 kcal/mol) (S15A and B Fig). It interacted with BACE1 by making three H-bonds with residues Thr232, Tyr198, and Asp32 (S15C and D Fig). Three critical residues were involved in the binding with compound 15, namely Tyr286 and Trp86 for AChE and Asp32 for BACE1. This study indicated that the top compounds interacted with critical residues of the target proteins, AChE and BACE1, inhibiting their physiological functions, which aligns with the study’s hypothesis. These findings suggest that the identified compounds may serve as promising leads for the development of dual-target inhibitors for Alzheimer’s disease. The next steps involve experimental validation to confirm their efficacy and safety in vitro and *in-vivo*. *C. asiatica* was the main source for twelve active dual inhibitors of AChE and BACE1, including centelloside A, centelloside F, centellasaponin A, centellasaponin I, centellasaponin H, scheffoleoside A, asiaticoside, 23-O-acetyl asiaticoside B, asiaticoside C, asiaticoside G, asiaticoside I, and 1,5-di-o-caffeoyl quinic acid, confirming the high potential of this plant for treatment of Alzheimer’s disease. This perennial aquatic herb is native to the Indian subcontinent and can be devoured as a culinary vegetable [53].

The binding profile analysis of the top 15 active ligands for human AChE indicated Asp74, Phe295, Ser293, Glu202, and Tyr341 as the most commonly involved residues for hydrogen bonding. Trp86, Trp286, Phe338, Tyr337, and Tyr341 displayed pi-alkyl or H-pi interactions. While aromatic groups of Trp86 and Trp286 mainly formed π-π stacking. The most common residues for BACE1 mainly involved Gly34, Arg128, Thr72, Thr232, Trp76, Asp32, Asp228, Ser325, Asn37, Arg235, and Lys321 for H-bonding and Trp76 for H-pi bonding. These findings concurred with the already-reported essential amino acids in the literature for the inhibition of AChE and BACE1 [28,29]. According to our results, compounds 1, 6, 7, 9, and 15 interacted with most of these critical residues.

Furthermore, we analyzed the probability distribution of hydrogen bond numbers to gain a more comprehensive insight into the interaction patterns (S2 Table). For AChE, the distribution indicates that 20% of compounds form 1 hydrogen bond, around 46.67% form 2 hydrogen bonds, 13.33% form 3 hydrogen bonds, 13.33% form 5 hydrogen bonds, and 6.67% form 7 hydrogen bonds. For BACE, approximately 33.33% of compounds form 2 hydrogen bonds, 20% form 3 hydrogen bonds, 26.67% form 4 hydrogen bonds, 13.33% form 6 hydrogen bonds, and 6.67% form 9 hydrogen bonds.

Surprisingly, compound 3, 5, and 6 formed mirrored H-bond numbers of the standards with the respective proteins (S2 Table). These results are noteworthy for their role as dual inhibitors, demonstrating potential for favorable binding to both target proteins. Additionally, compounds 11 and 12 formed the same number of hydrogen bonds with BACE1 as elenbecestat. Other than that, all other compounds formed a greater number of hydrogen bonds and hydrophobic interactions, indicating their stronger stability with the active sites of both targets. In general, compounds formed more bonds with BACE (average bond number 3.7) compared to AChE (average bond number 2.6). These findings suggest a higher affinity of these compounds for BACE1 over AChE. These results represent the variable binding affinities for AChE and BACE1 across different compounds.

### Pose validation

**Fig 5:**
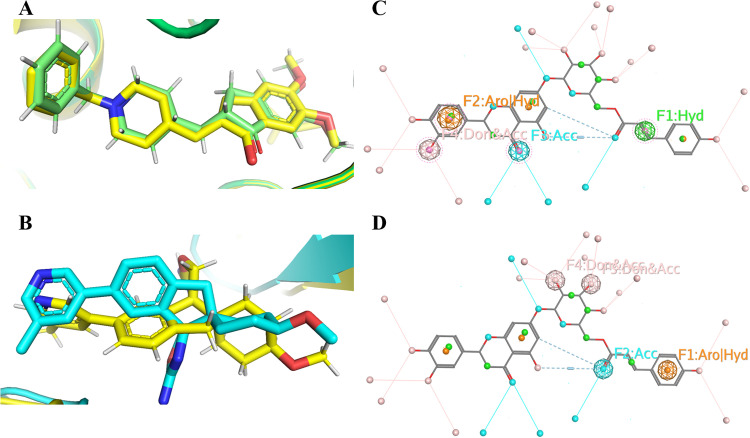
Superimposed structure of (A) native (yellow) and re-docked B7T (green), (B) native (yellow) and re-docked donepezil (green), (C) pharmacophore model with receptor acetylcholinesterase (AChE, PDB: 6O4W), and (D) pharmacophore model with receptorβ-secretase (BACE1, PDB: 6EJ3) generated by ZINC Pharmer. Hydrogen bond acceptors are depicted as orange spheres, hydrogen bond donors as white spheres, hydrophobic characteristics as green spheres, and aromatic rings as purple spheres.

As performed in a recent study [58], using redocking and superimposition techniques, we implemented a strict validation approach to guarantee the accuracy and the screening’s reliance on docking. The root mean square deviation values of the native and re-docked poses of co-crystallized ligands of human AChE and BACE1 were determined subsequently (Fig 5A and B). PyMOL software predicted the RMSD values to be 0.00 and 0.183 Å for AChE and BACE1, respectively. As the values determined were very low and satisfactory, they validated the docking algorithm’s capability to reproduce the native conformations of docked ligands [78].

### Pharmacophore features of notable compounds

The pharmacophore features of 15 top-ranked compounds were determined by the Pharma Gist webserver. Electronic and steric features of the pharmacophore are critical for strong binding interactions in a ligand-receptor complex to hinder the biological activity of the receptor. The features mainly included H-bond acceptor, aromatic rings, hydrophobic, positive ionizable, negative ionizable, and the number of features (Table 2). Compound 1 (tannic acid) showed the highest number of 57 features in corroboration with docking and MD simulation results, including 11 aromatic ring features, 46 hydrogen acceptors, and 4 hydrogen donors. All compounds showed higher features than the standards donepezil (12 features) and elenbecestat (11 features). (S)-eriodictyol 7-O-(6-β-O-trans-p-coumaroyl)-β-d-glucopyranoside (Compound 6) showed the most comparable pharmacophore features to the positive control. It displayed 19 features, including 1 hydrophobic, 5 aromatic, and 13 hydrogen acceptors.

**Table 2:** Pharmacophore features of top 15 compounds by Pharma Zist.

Ligand No./ Standards	Atoms	Features	Spatial features	Aromatic	Hydrophobic	Donor	Acceptors	Negatives	Positives
S1(Donepezil)	57	12	12	2	6	0	4	0	0
S2 (Elenbecestat)	49	11	11	2	1	3	5	0	0
1	143	57	32	11	0	04	46	0	0
2	100	51	32	3	29	0	19	0	0
3	97	50	32	3	31	0	16	0	0
4	105	49	32	3	25	0	21	0	0
5	100	51	32	3	29	0	19	0	0
6	56	19	19	5	1	0	13	0	0
7	102	50	32	3	27	0	20	0	0
8	98	44	32	2	30	0	12	0	0
9	97	47	32	3	24	0	20	0	0
10	97	46	32	3	23	0	20	0	0
11	113	54	32	4	25	0	25	0	0
12	97	49	32	3	27	0	19	0	0
13	100	48	32	3	25	0	20	0	0
14	100	51	32	3	29	0	19	0	0
15	60	23	17	2	2	6	12	0	0

The MOE pharmacophore query was employed to generate the pharmacophore model for both receptors, i.e., AChE and BACE1, highlighting the comprehensive set of features essential for interactions. Compound 6 displayed a comparable number of features for both receptors. This is also evident from the mirrored H bond numbers of the standards with the respective proteins. Therefore, the pharmacophore model of compound 6 was generated for the active sites of AChE (Fig 5C) and BACE1 (Fig 5D).

The pharmacophoric features of compound 6 that have been found with the active sites of the AChE and BACE offer significant knowledge on the molecular interactions required for efficient inhibition of both enzymes. It is noteworthy that compound 6 shows comparable properties in its interactions with BACE1 and AChE, suggesting a possible dual-inhibition ability (Fig 3C and 3D). The importance of nonpolar interactions for stabilizing ligand-protein complexes is highlighted by the hydrophobic characteristic seen in both AChE and BACE1 interactions. Furthermore, the existence of acceptor characteristics in both situations highlights how crucial hydrogen bond acceptors are for forming certain interactions inside the corresponding enzyme active sites. Moreover, the combination of aromatic and hydrophobic attributes detected in both AChE and BACE1 interactions underscores the crucial function of aromatic moieties possessing hydrophobic attributes in enhancing binding affinity. These shared features suggest that compound 6 holds promise as a dual inhibitor targeting both AChE and BACE1, which could be advantageous for therapeutic interventions, particularly in the context of AD. This approach allows us to expand the chemical space explored for potential inhibitors, providing a broader basis for drug discovery beyond currently known structures.

### Structural chemistry of notable compounds

**Fig 6:**
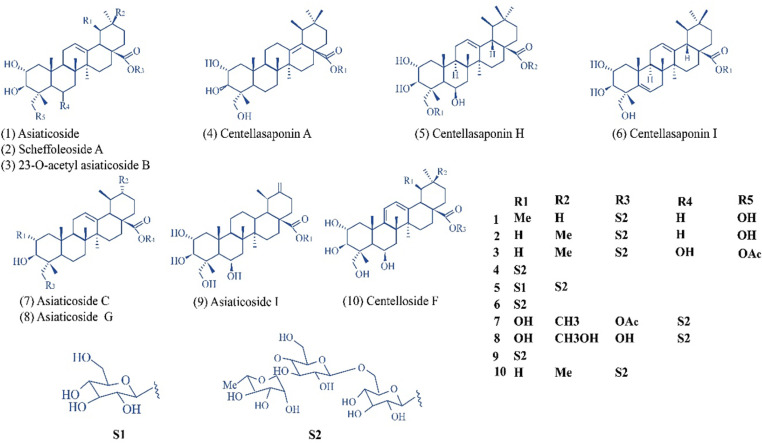
Structural chemistry of 10 ursane-type triterpene saponins as dual inhibitors of acetylcholinesterase (AChE) and β-secretase (BACE1).

In our virtual screening results for the identification of dual inhibitors, among the top 15 compounds, 10 compounds were found to be having the common structural moieties (Fig 6). Thse compounds included centelloside F, scheffoleoside A, asiaticoside, asiaticoside C, asiaticoside G, asiaticoside I, 23-O-acetyl asiaticoside B, centellasaponin A, centellasaponin H, and centellasaponin I, isolated from the perennial herb *C. asiatica*. They are classified as ursane-type triterpene saponins, based on their core structure, which is crucial for their biological activity. Such triterpenoids are known for their wide range of pharmacological properties, including anti-inflammatory, anticancer, and antimicrobial activities and neuroprotective effects [54]. Among the top compounds, two belong to the class of dammarane-type triterpene saponins, namely, centelloside A and sitoindoside-X. Like Ursane saponins, dammarane saponins exhibit a triterpene backbone (a four-ring structure in contrast to the five-ring structure in Ursane saponins) with various sugar moieties attached [79]. The discovery that these common structural elements exhibit dual inhibitory activity against AChE and BACE is notable.

The binding modes of 10 ursane saponins and 2 dammarane saponins indicated that the glycone groups engaged in H bonding with active site residues, a critical interaction across almost all top compounds, highlighting its role in binding affinity and specificity towards both AChE and BACE. On the other hand, the triterpenoid backbone (aglycone) more frequently participated in hydrophobic and aromatic interactions, anchoring the compounds within the active sites, thereby stabilizing the inhibitor-enzyme complex. The combined hydrophilic and hydrophobic interactions of these triterpene saponins represent their strong efficacy as dual inhibitors and guide the rational design of novel therapeutic agents targeting AD.

Our top compounds, particularly ursane saponins and dammarane saponins, and our standards, donepezil, and elenbecestat, exhibit distinct structural features. The saponins have a triterpene backbone with attached sugar moieties and hydroxyl groups. Donepezil contains a piperidine ring, while elenbecestat features a heterocyclic ring system (S2 Fig), which are responsible for their binding with the active sites of AChE and BACE1, respectively (S2 Fig). In contrast to the amine and other functional groups in donepezil and elenbecestat, triterpene saponins lack these features. Furthermore, the saponins are larger and more complex molecules compared to the relatively smaller structures of donepezil and elenbecestat. It is noteworthy that both standards are the inhibitors of their respective enzymes, and the triterpene saponins provide unique structural scaffolds as the dual inhibitors for the treatment of AD.

### Molecular dynamic simulation

**Fig 7:**
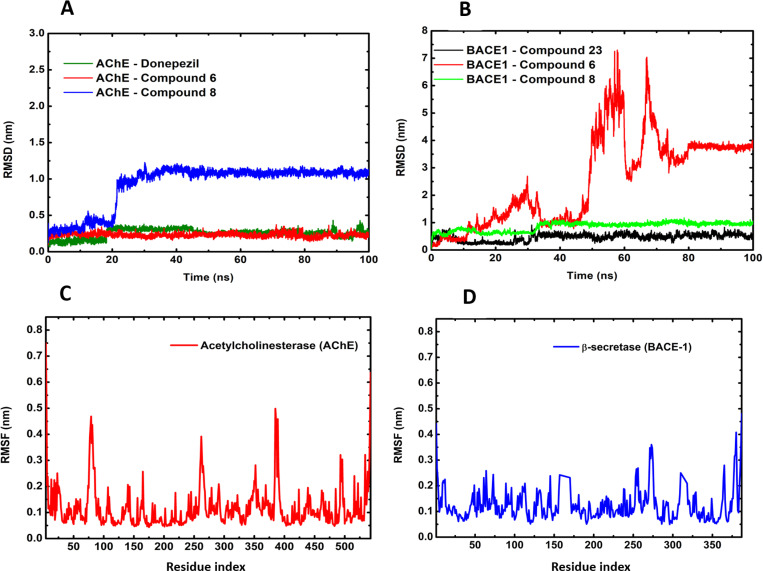
Molecular Docking (MD) simulation trajectory plot of acetylcholinesterase (AChE) and β-secretase (BACE1) exploring molecular variation with the calculation of protein backbone root mean square deviation (RMSD) of each ligand-protein complex and root mean square fluctuation (RMSF) of the free protein. (A) Superposition of the three ligands (compound 6, 8, and donepezil) highlighting increased molecular stability for the inhibitors compound 6 and donepezil, with stability peaking after 20 ns for compound 8. (B) compound 23, compound 6, and compound 8 for the system BACE1, with low deviation in RMSD for the compounds 23 and 8. (C) RMSF calculation for protein AChE shows the regions of flexibility and rigidity of the structural dynamics in 100 ns, and (D) RMSF calculation for protein BACE1.

To investigate the stability of the protein-ligand docked complex with the best interactions and the binding pose produced by docking, MD simulations under physiological conditions were performed. The stability of a protein system, or protein-ligand complex, can be calculated on a nanometric scale (nm) over a given time period, such as in nanoseconds (ns). During the MD trajectory, the small-scale RMSD deviation, up to 0.3 nm, shows the stability of the complex under analysis. Correspondingly, the RMSD graph for the AChE-Donepezil (Standard) and AChE-Compound 6 (S-eriodictyol7-O-(6-β-O-trans-p-coumaroyl)-β-d-glucopyranoside) complexes converged well and exhibited RMSD values below 0.5 nm, consistent with other studies [80,81]. However, the AChE-Compound 8 (Sitoindoside-X) system showed variations from 0.12–0.92 nm, with a stability peak of 1.0 nm after 20 ns of simulation (Fig 7A). After, we observed that the BACE1-Compound 23 (Standard) system showed variations of about 0.5 nm; the BACE1-Compound 8 (Sitoindoside-X) system showed similar variations and the BACE1-Compound 6 (S-eriodictyol7-O-(6-β-O-trans-p-coumaroyl)-β-d-glucopyranoside) system showed variations till 7.0 nm (Fig 7B). The deviations for BACE1 being higher than 0.3 nm, the system presented conformational and thermodynamic stability, and the average values are lower compared to the results of another study, which reported 1.5 nm. The differential shapes and flexibility of the binding sites for AChE and BACE1 account for the greater RMSD values for compound 8 with AChE and compound 6 with BACE1 when compared to their interactions with the other proteins. Each protein has a distinct structure, which might cause the identical molecule to interact differently. This can lead to increased mobility (higher RMSD) in one protein but not in the other. This occurs because certain compounds may require more conformational changes to bind in one protein than another because they fit more readily into the binding pocket of that protein. These variations demonstrate how the chemicals adjust to the unique structure of each protein rather than pointing to a weaker affinity. The RMSF was conducted for both targets, BACE1 and AChE, with their respective systems. It was observed that there were no significant fluctuations throughout the trajectory; both systems remained stable with values below 0.3 nm (Fig 7C and D).

**Fig 8:**
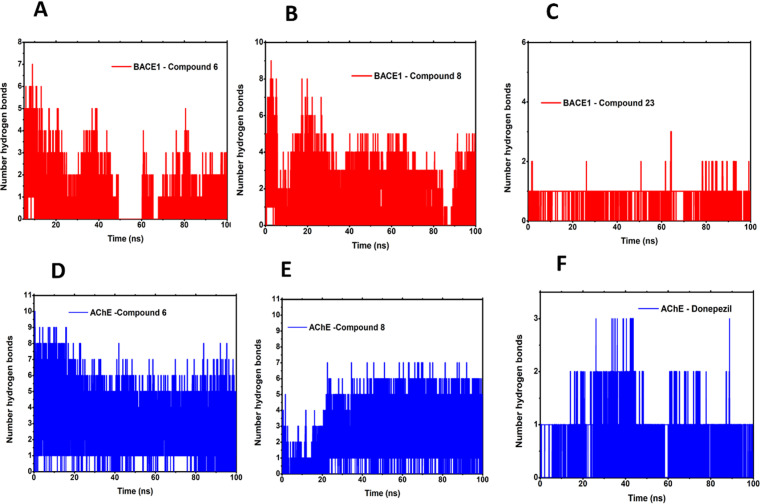
Projection of the atoms of the acetylcholinesterase (AChE) and*β*-secretase (BACE1) proteins in physical motion in the binding of hydrogen interactions in 100 ns. (A) AChE with compound 6; (B) AChE with compound 8; (C) AChE with Donepezil; (D) BACE1 with compound 6; (E) BACE1 with compound 8; (F) BACE1 with compound 23.

**Fig 9:**
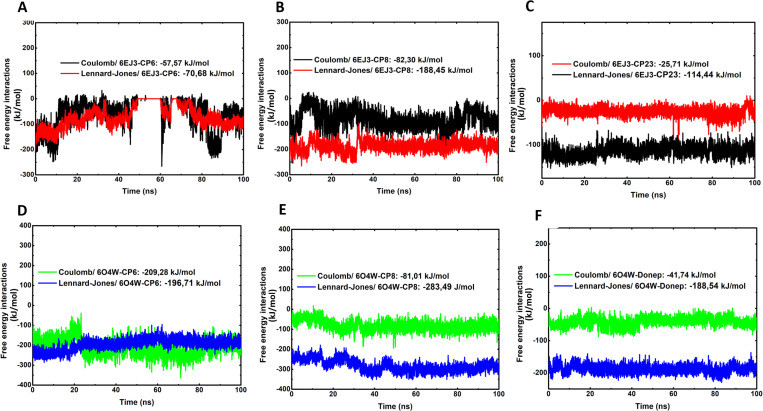
Distribution panel of short-range Coulombic interaction energy and short-range Lennard-Jones energy for both systems: acetylcholinesterase (AChE, PDB: 6O4W) andβ-secretase (BACE1, PDB: 6EJ3). (A) The 6ej3-cp6 system obtained a Coulomb interaction energy of −57.57 kJ/mol and a Lennard-Jones energy of −70.68 kJ/mol, with a total energy of −128.25 kJ/mol. (B) The 6ej3-cp8 system obtained a Coulomb interaction energy of −82.30 kJ/mol and a Lennard-Jones energy of −188.45 kJ/mol, with a total energy of −270.75 kJ/mol. (C) The 6ej3-cp23 system obtained a Coulomb interaction energy of −25.71 kJ/mol and a Lennard-Jones energy of −114.44 kJ/mol, with a total energy of −140.15 kJ/mol. (D) Whereas the 6o4w-cp6 system obtained a Coulomb interaction energy of −209.28 kJ/mol and a Lennard-Jones energy of −196.71 kJ/mol, with a total energy of −405.99 kJ/mol. (E) For the 6o4w-cp8, we obtained a Coulomb interaction energy of −81.01 kJ/mol and a Lennard-Jones energy of −283.49 kJ/mol, with a total energy of −364.05 kJ/mol. (F) Finally, the 6o4w-Donepe obtained a Coulomb interaction energy of −41.74 kJ/mol and a Lennard-Jones energy of −188.54 kJ/mol, with a total energy of −230.28 kJ/mol. cp6: compound 6; cp8: compound 8; cp23; Compound 23 (standard); Donep: Donepezil (standard).

In quantifying the number of hydrogen bonds between the protein and the ligand, the participation of these interactions throughout the simulation became evident, with greater incidence at the active sites. A high frequency of hydrogen bond interactions was observed between the AChE and compounds 6 and 8 in comparison to the standard donepezil (Fig 8A, B, and C). Similarly, a high frequency of hydrogen bonds was observed between BACE1, and Compound 6 and 8 with reference to the standard compound 23 (Fig 8D-F), evidencing the complementarity between the catalytic pocket of the target and the ligands. Additionally, the Coul-SR and LJ-SR terms were respectively calculated for each system, namely, 6EJ3-CP6 (−57,57 kJ/mol, −70,68 kJ/mol), 6EJ3-CP8 (−82,30 kJ/mol, −188,45 kJ/mol), 6EJ3-CP23 (−25,71 kJ/mol, −114,44 kJ/mol), 6O4W-CP6 (−209,28 kJ/mol, −196,71 kJ/mol), 6O4W-CP8 (−81,01 kJ/mol, −283,49 kJ/mol) and 6O4W-Donep (−41,74 kJ/mol, −188,54 kJ/mol). (Fig 9A-F). Thus, it is clear that the complexes 6o4w-CP6, 6o4w-CP8, 6ej3-CP6, and 6ej3-CP8 are strong candidates against Alzheimer’s disease.

**Fig 10:**
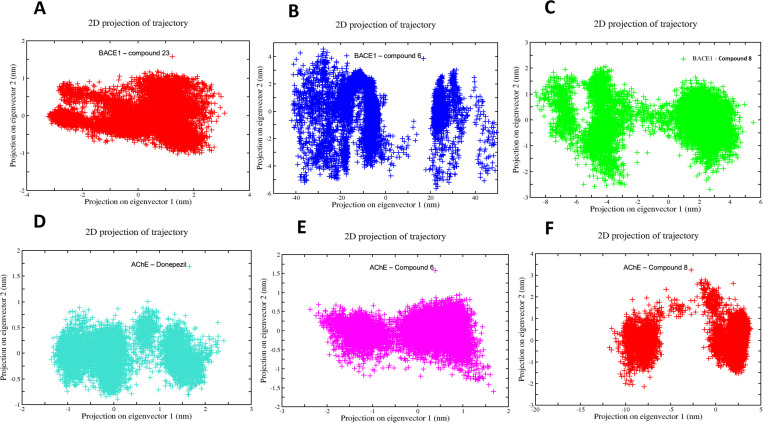
Comparison of the 2D projection of the two eigenvectors corresponding to the conformational fluctuations of the receptor systems: acetylcholinesterase (AChE, PDB:6O4W) and β-secretase (BACE1, PDB: 6EJ3), BACE1-compound 23 (A); BACE1-compound 6 (B); BACE1-compound 8 (C); AChE-donepezil (D); AChE-compound 6 (E); AChE-compound 8 (F).

**Fig 11:**
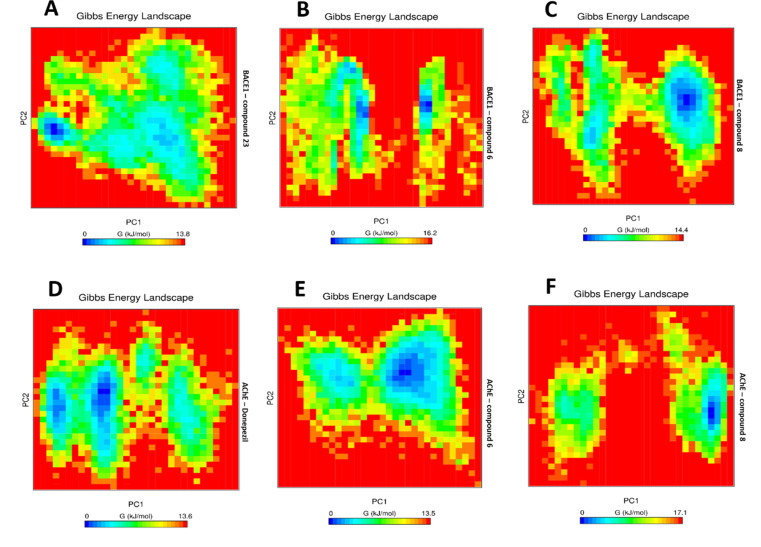
The Gibbs free energy landscape for acetylcholinesterase (AChE) and*β*-secretase (BACE1), plotted using PC1 and PC2, illustrates lower energy systems as represented by deeper blue regions on the contour map. BACE1-compound 23 (A); BACE1-compound 6 (B); BACE1-compound 8 (C); AChE-Donepezil (D); AChE-compound 6 (E); AChE-compound 8 (F). G: Gibbs free energy (kJ/mol); PC1: Principal Component 1; PC2: Principal Component 2.

In PCA, the sum of the eigenvalues suggests the overall flexibility of a structure under varying conditions. Our results, based on the first five eigenvectors, captured the most important collective motions of the inhibitor/molecular target interaction during a 100 ns simulation. These five eigenvectors accounted for fluctuations in the system after each ligand’s binding to their respective targets. The 2D graphs generated for the BACE1-compound 23 co-crystallized systems demonstrated greater stability compared to the systems with inhibitors compound 6 and compound 8 (Fig 10A-C). Similarly, the 2D graph of the AChE-Donepezil co-crystallized system showed greater stability compared to its inhibitors, compound 6 and compound 8 (Fig 10D-F). These results suggest that the complexes formed with co-crystallized ligands exhibit less correlated movements and, therefore, greater stability compared to the inhibitors. The Gibbs free energy landscape suggests thermodynamic stability for the co-crystallized BACE1-compound 23 system, with a lower free energy of 13.8 kJ/mol compared to BACE1-compound 6 at 16.2 kJ/mol and BACE1-compound 8 at 14.4 kJ/mol (Fig 11A-C). Similarly, the results obtained for the AChE/inhibitor systems showed a similar interaction energy profile, with the lowest energy for the AChE-compound 6 complex at 13.5 kJ/mol, followed by the interaction energy of AChE-donepezil at 13.6 kJ/mol and AChE-compound 8 at 17.1 kJ/mol (Fig 11D-F). These results indicate that the interactions formed by the co-crystallized compounds provide more stable conformations, which may be crucial for understanding the efficiency and selectivity of these ligands with respect to their molecular targets.

### Drug likeness and pharmacokinetic analysis

The smile format of fifteen top-hit compounds was given as input to the molinesperation online server. It predicted the drug-likeness of compounds by anticipating their bioactivity and physiochemical properties. These properties mainly included log P (≤ 5), polar surface area (<140 Å), molecular weight (<500 Da), number of rotatable bonds (>6), number of OH bonds (≤ 10), number of OH and NH bonds (≤ 5), violations, and molecular volume (Table 3). Compound 6 and 15, named S-eriodictyol7-O-(6-β-O-trans-p-coumaroyl)-β-d-glucopyranoside and 1,5-di-o-caffeoyl quinic acid, showed molecular properties comparable to those of the standard donepezil and elenbecestat. Compounds 6 and 15 showed molecular weights of 596.54 and 516.46 Da, respectively. The molecular weight limit for AChE has been increased to 600 Da due to its greater active site [82]. Compounds having molecular weights up to 400–600 Da have the ability to cross the blood–brain barrier [83]. As the majority of top hit ligands were triterpenoids derived from *C. asiatica* extracts, their oral bioavailability can be further improved by using a variety of bioenhancers like Centell-S [84].

**Table 3:** Molecular and biological properties of top 15 compounds.

Ligand No./ Standards	Log p	PSA (Å²)	nAtoms	M.Wt (g/mol)	nOH	nOHNH	nViolations	nrotb	Volume (Å^3^)	GPCR	ICM	KI	NRL	PI	EI
S1 (Donepezil)	4.1	38.78	28	379.5	4	0	0	6	367.89	0.22	−0.14	−0.16	0.03	0.03	0.25
S2 (Elenbecestat)	2.55	102.5	30	437.45	7	3	0	4	350.45	−0.24	−0.42	−0.34	−0.73	−0.21	−0.18
1	7.06	777.98	122	1701.21	46	25	4	31	1305.93	−4.06	−4.07	−4.08	−4.08	−4.04	−4.05
2	0.14	315.21	67	959.13	19	12	3	10	875.52	−3.44	−3.71	−3.69	−3.59	−3.04	−3.32
3	3.19	257.68	63	899.12	16	10	3	12	846.09	−2.81	−3.51	−3.54	−3.21	−2.24	−2.79
4	0.09	341.52	71	1017.17	21	12	3	12	920.1	−3.6	−3.75	−3.77	−3.68	−3.45	−3.53
5	0.3	315.21	67	959.13	19	12	3	10	875.55	−3.39	−3.71	−3.69	−3.58	−3	−3.28
6	2.21	212.67	43	596.54	13	7	3	8	497.19	−0.02	−0.61	−0.41	−0.05	0	−0.04
7	1.07	321.29	70	1001.17	20	11	3	12	912.41	−3.57	−3.74	−3.77	−3.64	−3.31	−3.51
8	9.04	181.59	62	871.16	12	4	3	22	846.24	−2.74	−3.4	−3.56	−3.09	−2.03	−2.52
9	−0.64	335.44	68	975.13	20	13	3	11	884.15	−3.46	−3.72	−3.71	−3.59	−3.08	−3.36
10	−0.36	335.44	68	977.15	20	13	3	10	890.15	−3.48	−3.69	−3.71	−3.61	−3.11	−3.37
11	−2.32	414.59	79	1137.27	25	16	3	13	1015.71	−3.76	−3.85	−3.87	−3.81	−3.71	−3.72
12	0.37	315.21	67	959.13	19	12	3	10	875.9	−3.38	−3.7	−3.7	−3.55	−2.96	−3.26
13	−0.8	335.44	68	973.12	20	13	3	10	877.38	−3.46	−3.72	−3.71	−3.61	−3.17	−3.33
14	0.12	315.21	67	957.12	19	12	3	10	869.33	−3.37	−3.71	−3.69	−3.58	−3.02	−3.26
15	1.42	211.28	37	516.46	12	7	3	9	431.08	0.18	0.04	−0.01	0.5	0.21	0.42

Log p: partition coefficient; PSA: polar surface area; nAtoms: number of atoms; M.Wt: molecular weight; nOH: number of hydroxyl groups; nOHNH: number of hydroxyl and amine groups; nViolations: number of violations of Lipinski’s rule of five; nrotb: number of rotatable bonds; Volume: molecular volume; GPCR: G-protein coupled receptor; ICM: ion channel modulators; KI: kinase inhibitors; NRL: nuclear receptor ligands; PI: protease inhibitors; EI: enzyme inhibitors.

This tool also predicted the bioactivity scores of the top 15 ligands by evaluating G protein-coupled receptor (GPCR) ligands, enzyme inhibitors, nuclear receptor ligands, kinase inhibitors, protease inhibitors, and ion channel modulator values. Compounds 6 and 15 showed bioactivity scores comparable to those of donepezil and elenbecestat (Table 3). DruLiTo software used physiochemical properties to identify many rules and filters that determine drug-likeness (Table 4). The MDDR rule was followed by all fifteen ligands, whereas the Veber rule was followed by compound 7 only. This implies that while all ligands have potential as drug candidates, only compound 7 is well-suited for oral administration, and the others may require optimization to improve their bioavailability. No ligand was reported to follow Lipinski’s rule of 5 completely. As Lipinski’s rule is mostly for drugs synthesized chemically and taken orally, natural products and their derivatives and substrates for biological transporters, as well as those compounds having large, flexible, and hydrophobic active sites, can be exempted [85]. There are significant examples of effective drugs like telaprevir and atazanavir that didn’t follow at least two of Lipinski’s rules [86]. So, these active ligands derived from Ayurvedic plants have significant potential to act as dual inhibitors of AChE and BACE1.

The CMC-50 (Combinatorial Methodology Center’s rule of 50) shows that none of the ligands, including the standards (donepezil and elenbecestat), comply with this rule, which often relates to medicinal chemistry and compound progression (Table 4). This may indicate potential challenges in progressing these compounds through certain stages of drug development. The blood-brain barrier (BBB) permeability results suggest that only donepezil can cross the BBB, as indicated by the “+” symbol. This is expected given its use as an Alzheimer’s drug. All other ligands fail to pass this barrier, which may limit their efficacy in treating CNS disorders unless modifications are made to improve brain penetration. Elenbecestat, despite also being a neurological drug, does not comply with the BBB rule.

**Table 4:** Drug likeness prediction by DruLito software.

Ligand No./ Standards	Lipinski’s Rule	Ghose’s Rule	CMC-50	MDDR	Veber Rule	BBB	Unweighted QED	Weighted QED
S1 (Donepezil)	+	–	–	+	+	+	+	+
S2 (Elenbecestat)	+	–	–	–	+	–	+	+
1	–	–	–	+	–	–	–	–
2	–	–	–	+	–	–	–	–
3	–	–	–	+	–	–	–	–
4	–	–	–	+	–	–	–	–
5	–	–	–	+	–	–	–	–
6	–	–	–	+	–	–	–	–
7	–	–	–	+	+	–	–	–
8	–	–	–	+	–	–	–	–
9	–	–	–	+	–	–	–	–
10	–	–	–	+	–	–	–	–
11	–	–	–	+	–	–	–	–
12	–	–	–	+	–	–	–	–
13	–	–	–	+	–	–	–	–
14	–	–	–	+	–	–	–	–
15	–	–	–	+	–	–	–	–

CMC-50: 50% Correct Mean Classification; MDDR: MDL Drug Data Report; BBB: Blood-Brain Barrier; Unweighted QED: Unweighted Quantitative Estimation of Drug-likeness; Weighted QED: Weighted Quantitative Estimation of Drug-likeness.

For the unweighted QED (Quantitative Estimate of Drug-likeness) and weighted QED, both standards score positively, indicating a high drug-likeness based on multiple physicochemical properties. In contrast, none of the other ligands meet these criteria when certain factors (like molecular weight, solubility, and permeability) are given more emphasis, which suggests they may need significant structural optimization to be considered drug-like.

The ADMET analysis of high-scoring compounds was performed in ADMET lab 2. It mainly analyzes log S, human intestinal absorption (HIA), Caco-2 permeability, clearance of drug (CL), CYP450 isozyme activity, half-life (T_1/2_), carcinogenicity, and toxicity (Table 5). All ligands except compound 1 and elenbecestat showed a normal range of log S (−4 to 0.5 log mol/L). Compounds 1, 6, 8, and 15 showed comparable inhibitory values for cytochrome P450 isozymes, including CYP 1A2, 2C19, 2C9, 2D6, and 3A4. The values range from 0 (non-inhibitor) to −1 (inhibitor). Compounds 1, 6, and 8 showed high clearance rates close to Donepezil (10.635 ml/min/kg) with the values of 12.8, 9.7, and 12.6 ml/min/kg, respectively. However, compound 15 showed a relatively low clearance rate (4.548 ml/min/kg) similar to Elenbecestat (4.805 ml/min/kg). All compounds showed moderate half-lives except 1, 14, and 15. Donepezil exhibits a moderate half-life, while Elenbecestat has a very short half-life. The half-life of compound 3 was comparable to Donepezil and it may offer similar pharmacokinetics. Human hepatotoxicity (H-HT) was very low for all compounds as compared to standards donepezil and elenbecestat. Compounds 1 and 6, along with the standards, showed a high toxicity value for drug-induced liver injury (DILI). The carcinogenicity (TD50) value has a range from 0 (non-carcinogen) to 1 (carcinogen), which is very low for all compounds, similar to the values of both the standards. An LD50-oral value of 0 showed all compounds had no acute toxicity when taken orally, confirming the non-toxic and non-carcinogenic nature of all compounds, similar to both the standards. This study showed all top-scored ligands have satisfactory ADMET profiles, comparable to the reference standards, fulfilling most of the criteria of drug-likeness, and thus are proposed as potential dual inhibitors of AChE and BACE1.

**Table 5:** ADMET (Absorption, Distribution, Metabolism, Excretion, and Toxicity) analyses of top compounds by ADMET Lab 2.

Ligand No./ Standards	LogS	HIA (%)	Caco-2	BBB	CYP1A2	CYP2C19	CYP2C9	CYP2D6	CYP3A4	CL (ml/min/kg)	T12 (h)	hERG	H-HT	DILI	FDAMDD	Carcin- ogenicity	LC50FM (mg/L)	LD50_ Oral (mg/kg)
S1 (Donepezil)	−4.307	0.003	−4.793	0.975	0.14	0.413	0.097	0.986	0.339	10.635	0.164	0.99	0.381	0.805	0.817	0.079	5.338	0
S2 (Elenbecestat)	−5.132	0.007	−5.043	0.402	0.313	0.468	0.203	0.046	0.093	4.805	0.029	0.915	0.78	0.93	0.972	0.076	3.308	0
1	1.308	1	−7.722	0	0.464	0.002	0.06	0	0	12.869	0.998	0.001	0.014	0.961	0.018	0.001	4.04	0
2	−2.017	0.997	−5.901	0.137	0	0	0	0	0.017	0.551	0.022	0.003	0.185	0.018	0.012	0.018	3.446	0
3	−2.988	0.974	−5.621	0.05	0	0	0	0	0.004	0.519	0.17	0.051	0.128	0.008	0.074	0.021	7.195	0
4	−3.056	0.952	−6.113	0.028	0.001	0.001	0	0.002	0.107	0.435	0.614	0.248	0.191	0.104	0.031	0.014	6.545	0
5	−2.163	0.995	−5.934	0.12	0	0	0	0	0.019	0.668	0.022	0.003	0.171	0.013	0.024	0.028	4.614	0
6	−4.406	0.861	−6.521	0.041	0.046	0.211	0.701	0.409	0.348	9.725	0.586	0.045	0.097	0.928	0.221	0.624	6.032	0
7	−2.748	0.998	−5.976	0.082	0	0	0	0	0.022	0.693	0.015	0.001	0.165	0.025	0.023	0.033	5.33	0
8	−4.3	0.268	−5.117	0.215	0.001	0.063	0.089	0.001	0.383	12.633	0.028	0.075	0.246	0.528	0.785	0.048	5.313	0
9	−1.502	0.999	−6.368	0.147	0	0	0	0	0.014	0.614	0.03	0.003	0.173	0.016	0.025	0.022	3.93	0
10	−2.787	0.989	−6.419	0.016	0.002	0	0	0	0.041	0.602	0.623	0.503	0.206	0.066	0.003	0.006	5.813	0
11	−2.606	0.998	−6.535	0.163	0	0	0	0	0.035	0.05	0.641	0.165	0.184	0.042	0.007	0.019	4.953	0
12	−2.296	0.997	−6.173	0.101	0	0	0	0	0.015	0.66	0.02	0.002	0.168	0.019	0.032	0.025	4.552	0
13	−3.106	0.979	−6.425	0.137	0	0.001	0	0	0.111	0.669	0.607	0.233	0.22	0.022	0.136	0.027	3.726	0
14	−3.545	0.95	−6.317	0.062	0.001	0.001	0	0.001	0.09	0.522	0.757	0.044	0.203	0.026	0.05	0.047	4.577	0
15	−2.026	0.916	−6.212	0.078	0.074	0.063	0.401	0.019	0.095	4.548	0.957	0.018	0.111	0.045	0.063	0.13	4.484	0

LogS: Logarithm of Solubility; HIA (%): Human Intestinal Absorption Percentage; Caco-2: Caco-2 Permeability Assay; BBB: Blood-Brain Barrier; CYP1A2, CYP2C19, CYP2C9, CYP2D6, CYP3A4: Cytochrome P450 Enzymes 1A2, 2C19, 2C9, 2D6, and 3A4; CL: Clearance; T12: Half-Life; hERG: human Ether-à-go-go-Related Gene inhibition; H-HT: Hepatotoxicity; DILI: Drug-Induced Liver Injury; FDAMDD: FDA Maximum Recommended Daily Dose; LC50FM: Lethal Concentration 50% in freshwater medium; LD50_Oral: Lethal Dose 50% for oral administration.

### Significance, limitations, and future directions

Compared to previous *in-vitro, in-vivo*, and *in-silico* studies by Maliszewski et al., Dhamodharan et al., and Wang et al. to find the dual inhibitors of AChE and BACE1, our *in-silico* approach integrates both virtual screening and MD simulations to evaluate dual inhibitors targeting AChE and BACE1 [15–17]. While our top-ranked structures, namely (S)-eriodictyol 7-O-(6-β-O-trans-p-coumaroyl)-β-D-glucopyranoside, sitoindoside-X, and 1,5-di-O-caffeoyl quinic acid, are distinct and structurally unique compared to the compounds identified in these studies, such as the 1,3,5-triazine ring and the furocoumarin scaffold of notopterol, our work aligns with and contributes to the broader context of ongoing research. Recognizing the potential of our top compounds in Alzheimer’s therapy, we uncover their feasibility as therapeutic agents, considering their efficacy, safety, and the practicality of their development and production. These compounds might integrate into or enhance current treatment paradigms for AD. Additionally, our compounds have the potential for translating laboratory findings into clinical applications and also for future research and development in this promising area.

However, this study may not capture the full biological complexity, and the findings are preliminary, utilizing only computational tools. As a next step, experimental validation of the pharmacological effects of top-ranked compounds is imperative. This should include applying strategies for lead optimization, assessing their ability to cross the blood-brain barrier, their metabolism and clearance rates, and their impact on cognitive functions in disease models. Additionally, exploring the synergistic effects of these compounds with existing Alzheimer’s treatments could provide insights into more effective combination therapies. Future research should also aim to expand the compound library to include more diverse chemical entities, potentially uncovering novel scaffolds for dual inhibition of AChE and BACE1. Future studies must consider adding dynamic cross-correlation map (DCCM) and probability density function (PDF) analyses. Further computational studies using higher-level methods, such as ONIOM (Our own N-layered Integrated Molecular Orbital and Molecular Mechanics) or other QM/MM (Quantum Mechanics/Molecular Mechanics) approaches, could add valuable additions to these results. These methods, especially with the availability of low-cost yet reliable DFT (Density Functional Theory) techniques, are highly suitable for analyzing drug-protein interactions.

To confirm the inhibition potency of the lead compounds, enzyme inhibition assays using recombinant human AChE and BACE1 enzymes could be conducted. Kinetic studies could be performed to elucidate the mode of inhibition. Furthermore, the compounds could be tested in appropriate animal models of AD to evaluate their ability to reduce amyloid-beta plaque accumulation and improve cognitive function. Additionally, toxicity studies should be performed to assess the safety profile of the compounds in the animal models.

## Conclusion

Alzheimer’s disease is a neurodegenerative disorder characterized by cognitive decline, driven by the accumulation of amyloid-beta plaques and neurofibrillary tangles. It involves the dysfunction of key enzymes such as AChE and BACE1, making them critical targets for therapeutic intervention. In this study, we aimed to explore the potential of 820 compounds from Ayurvedic medicinal plants in developing dual inhibitors that can target both AChE and BACE1 to offer a more comprehensive therapeutic strategy for AD. The top fifteen lead dual inhibitors are reported by the extensive analysis of molecular docking, MD simulations, pharmacophores, drug-likeness, and ADMET analyses. Compounds 6, 8, and 15, namely (S)-eriodictyol 7-O-(6-β-O-trans-p-coumaroyl)-β-d-glucopyranoside, (which also presented mirrored H bond numbers for both proteins compared to standards) sitoindoside-X and 1,5-di-o-caffeoyl quinic acid had the most comparable drug-like properties required to be a potential lead compound. Among the top compounds, these three compounds have the strongest potential to be strongly accommodated in the active sites of target enzymes and might have BBB penetration potential. The MD simulation studies of these compounds supported this hypothesis, representing the stability in the active sites. This study also elaborated that most of the compounds that showed high dual inhibition of AChE and BACE1 were triterpene saponins belonging to the plant *Centella asiatica* (L.) Urb., highlighting their affinity for the active sites of both proteins as well as emphasizing the high potency of *C. asiatica* to be used for the treatment of Alzheimer’s disease. The crucial outcomes of this study underscore the potential of Ayurvedic compounds, particularly those from *C. asiatica*, as dual inhibitors of AChE and BACE1. It leads to a significant leap forward in the quest for more effective Alzheimer’s treatments. Further experimental validation is required to exploit the therapeutic potential of these lead compounds for the treatment of AD.

## Supporting information

S1 Fig 2D ligand interaction profiles: (A) Compound 1 with acetylcholinesterase (AChE) and (B) Compound 1 with β-secretase (BACE1). (TIF)

S2 Fig 2D ligand interaction profiles: (A) Standard Donepezil with acetylcholinesterase (AChE) and (B) Standard elenbecestat with β-secretase (BACE1). (TIF)

S3 Fig 2D ligand interaction profiles: (A) Compound 2 with acetylcholinesterase (AChE), (B) Compound 2 with β-secretase (BACE1), (C) Compound 3 with AChE and (D) Compound 3 with BACE1. (TIF)

S4 Fig Ligand-protein interactions of Compound 4 (purple) with acetylcholinesterase (AChE) and β-Secretase (BACE1). (A) 3D (B) 2D interaction profile of compound 4 with AChE; (C) 3D and (D) 2D interaction profile of compound 4 with BACE1. Residues from AChE are highlighted in blue and residues from BACE1 are highlighted in green.
(TIF)

S5 Fig Ligand-protein interactions of Compound 5 (cyan) with acetylcholinesterase (AChE) and β-Secretase (BACE1). (A) 3D (B) 2D interaction profile of compound 5 with AChE; (C) 3D and (D) 2D interaction profile of compound 5 with BACE1. Residues from AChE are highlighted in blue and residues from BACE1 are highlighted in green.
(TIF)

S6 Fig Ligand-protein interactions of Compound 6 with acetylcholinesterase (AChE) and β-Secretase (BACE1). (A) 3D (B) 2D interaction profile of compound 6 with AChE; (C) 3D and (D) 2D interaction profile of compound 6 with BACE1. Residues from AChE are highlighted in blue and residues from BACE1 are highlighted in green.
(TIF)

S7 Fig Ligand-protein interactions of Compound 7 with acetylcholinesterase (AChE) and β-Secretase (BACE1). (A) 3D (B) 2D interaction profile of compound 7 with AChE; (C) 3D and (D) 2D interaction profile of compound 7 with BACE1. Residues from AChE are highlighted in blue and residues from BACE1 are highlighted in green.
(TIF)

S8 Fig Ligand-protein interactions of Compound 8 with acetylcholinesterase (AChE) and β-Secretase (BACE1). (A) 3D (B) 2D interaction profile of compound 8 with AChE; (C) 3D and (D) 2D interaction profile of compound 8 with BACE1. Residues from AChE are highlighted in blue and residues from BACE1 are highlighted in green.
(TIF)

S9 Fig Ligand-protein interactions of Compound 9 with acetylcholinesterase (AChE) and β-Secretase (BACE1). (A) 3D (B) 2D interaction profile of compound 9 with AChE; (C) 3D and (D) 2D interaction profile of compound 9 with BACE1. Residues from AChE are highlighted in blue and residues from BACE1 are highlighted in green.
(TIF)

S10 Fig Ligand-protein interactions of Compound 10 with acetylcholinesterase (AChE) and β-Secretase (BACE1). (A) 3D (B) 2D interaction profile of compound 10 with AChE; (C) 3D and (D) 2D interaction profile of compound 10 with BACE1. Residues from AChE are highlighted in blue and residues from BACE1 are highlighted in green.
(TIF)

S11 Fig Ligand-protein interactions of Compound 11 with acetylcholinesterase (AChE) and β-Secretase (BACE1). (A) 3D (B) 2D interaction profile of compound 11 with AChE; (C) 3D and (D) 2D interaction profile of compound 11 with BACE1. Residues from AChE are highlighted in blue and residues from BACE1 are highlighted in green.
(TIF)

S12 Fig Ligand-protein interactions of Compound 12 with acetylcholinesterase (AChE) and β-Secretase (BACE1). (A) 3D (B) 2D interaction profile of compound 12 with AChE; (C) 3D and (D) 2D interaction profile of compound 12 with BACE1. Residues from AChE are highlighted in blue and residues from BACE1 are highlighted in green.
(TIF)

S13 Fig Ligand-protein interactions of Compound 13 with acetylcholinesterase (AChE) and β-Secretase (BACE1). (A) 3D (B) 2D interaction profile of compound 13 with AChE; (C) 3D and (D) 2D interaction profile of compound 13 with BACE1. Residues from AChE are highlighted in blue and residues from BACE1 are highlighted in green.
(TIF)

S14 Fig Ligand-protein interactions of Compound 14 with acetylcholinesterase (AChE) and β-Secretase (BACE1). (A) 3D (B) 2D interaction profile of compound 14 with AChE; (C) 3D and (D) 2D interaction profile of compound 14 with BACE1. Residues from AChE are highlighted in blue and residues from BACE1 are highlighted in green.
(TIF)

S15 Fig Ligand-protein interactions of Compound 15 with acetylcholinesterase (AChE) and β-Secretase (BACE1). (A) 3D (B) 2D interaction profile of compound 15 with AChE; (C) 3D and (D) 2D interaction profile of compound 15 with BACE1. Residues from AChE are highlighted in blue and residues from BACE1 are highlighted in green.
(TIF)

S1 Table Library of phytoconstituents obtained from selected ayurvedic plants. The docking scores of these phytochemicals are mentioned with acetylcholinesterase (AChE) and β-secretase (BACE1).
(DOCX)

S2 Table Ligand Interaction report of dual inhibitors of human acetylcholinesterase (AChE, PDB: 6O4W) and β-secretase (BACE1, PDB: 6EJ3). (DOCX)
