# Supplementary figures and images for: Computer-aided discovery of dual-target compounds for Alzheimer’s from ayurvedic medicinal plants
Source: PLoS One. 2025 Jun 25;20(6):e0325441. doi: 10.1371/journal.pone.0325441 (PMC12193798; doi:10.1371/journal.pone.0325441)

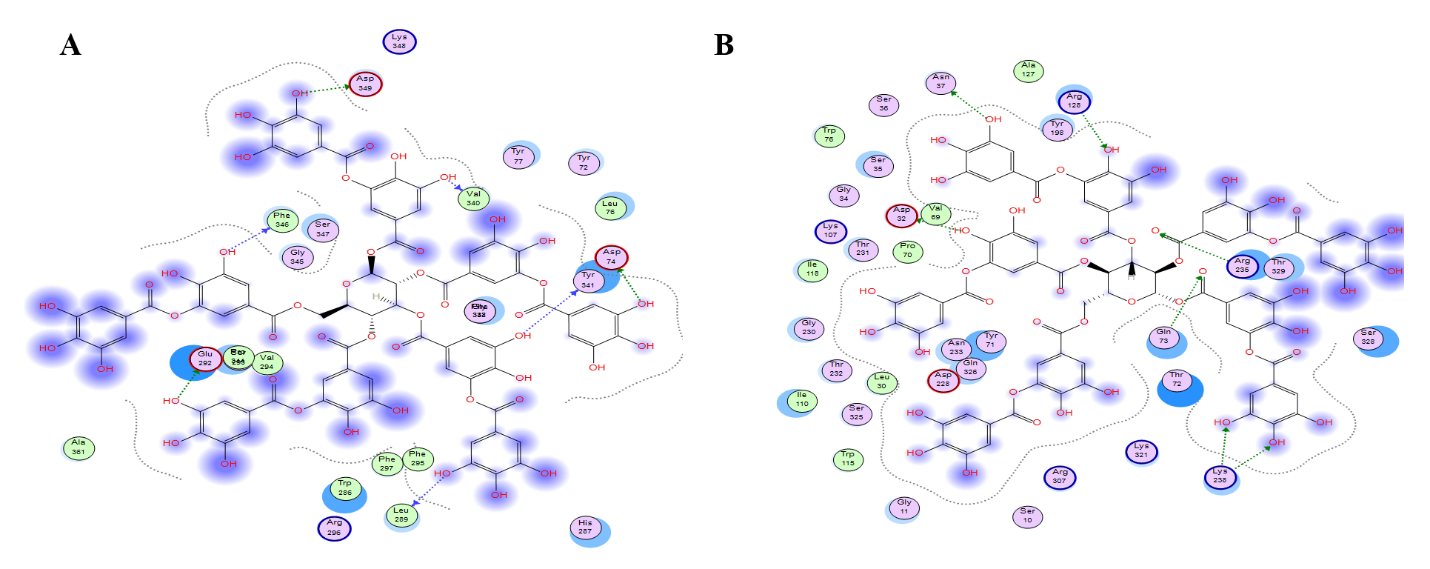

Supplement: S1 Fig — (TIF) [file pone.0325441.s001.tif]

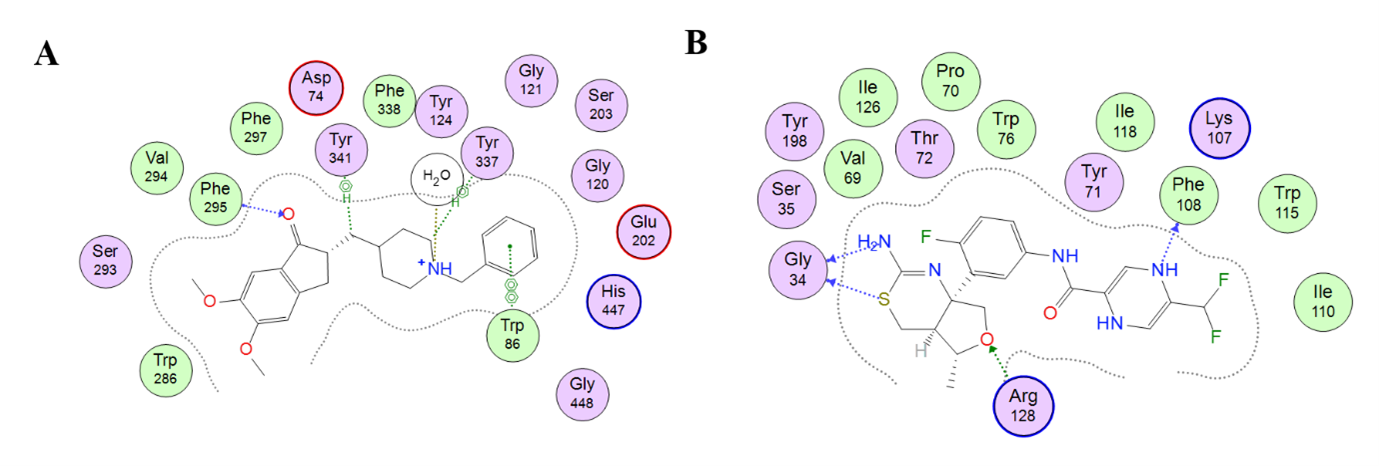

Supplement: S2 Fig — (TIF) [file pone.0325441.s002.tif]

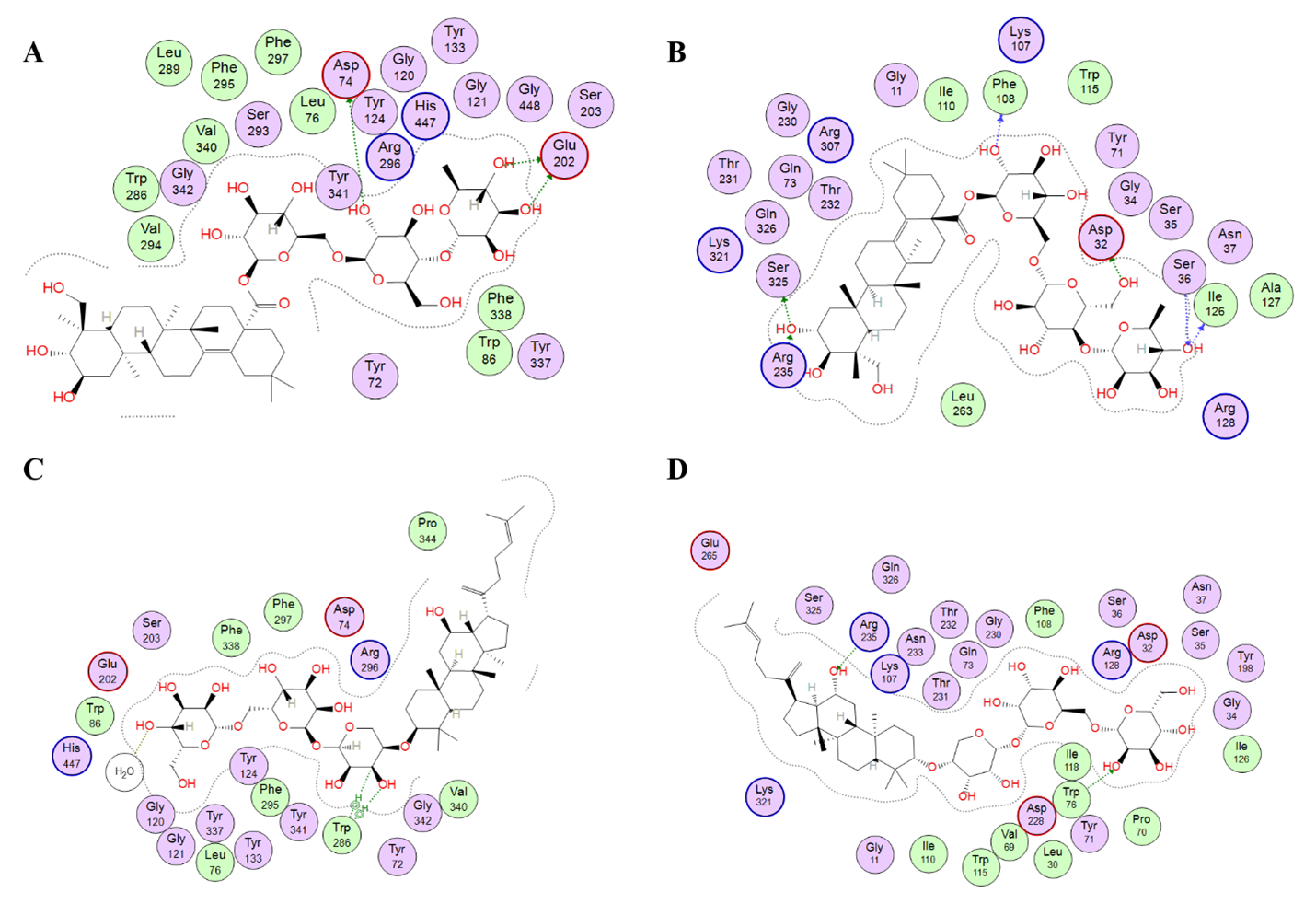

Supplement: S3 Fig — (TIF) [file pone.0325441.s003.tif]

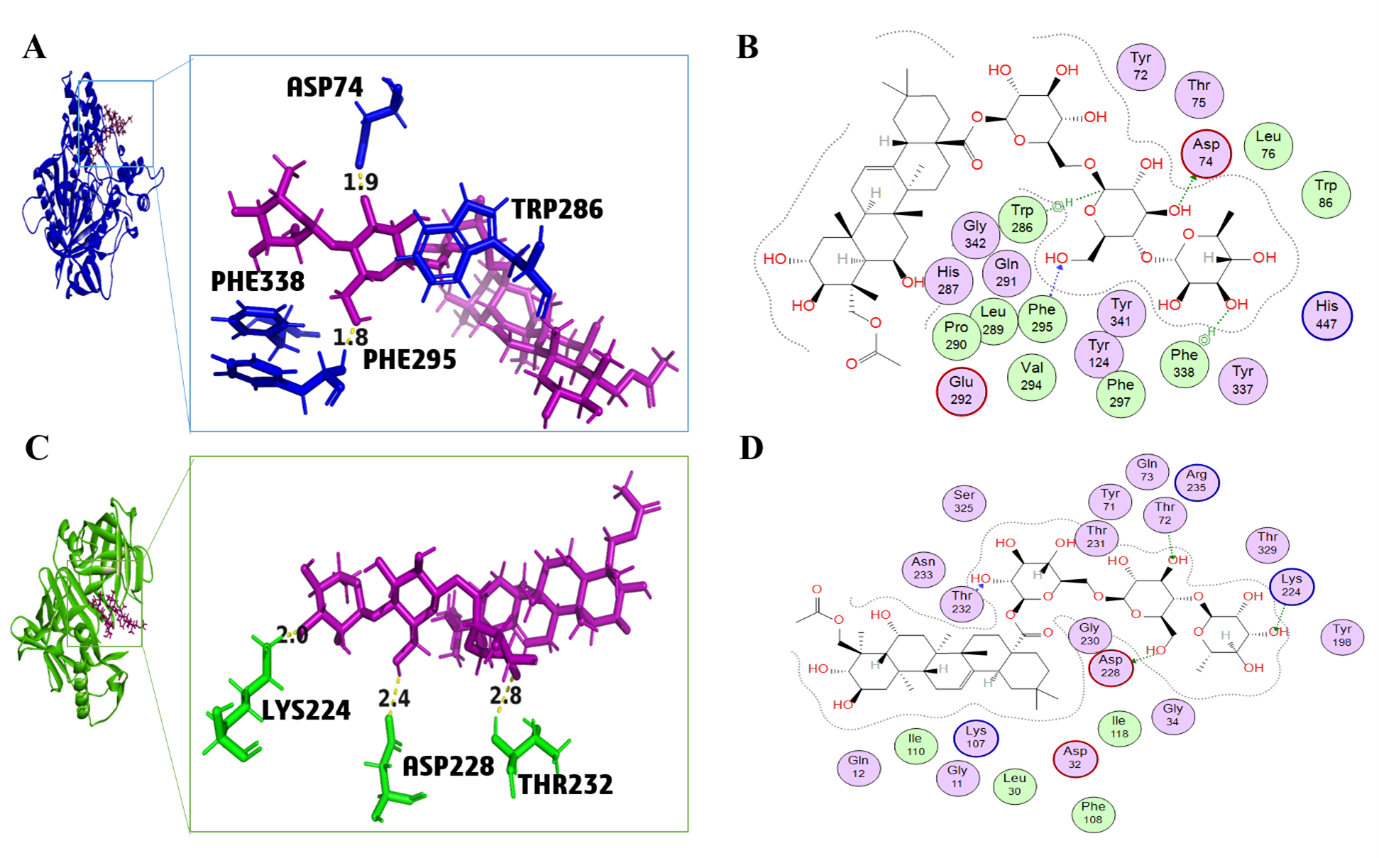

Supplement: S4 Fig — (A) 3D (B) 2D interaction profile of compound 4 with AChE; (C) 3D and (D) 2D interaction profile of compound 4 with BACE1. Residues from AChE are highlighted in blue and residues from BACE1 are highlighted in green. (TIF) [file pone.0325441.s004.tif]

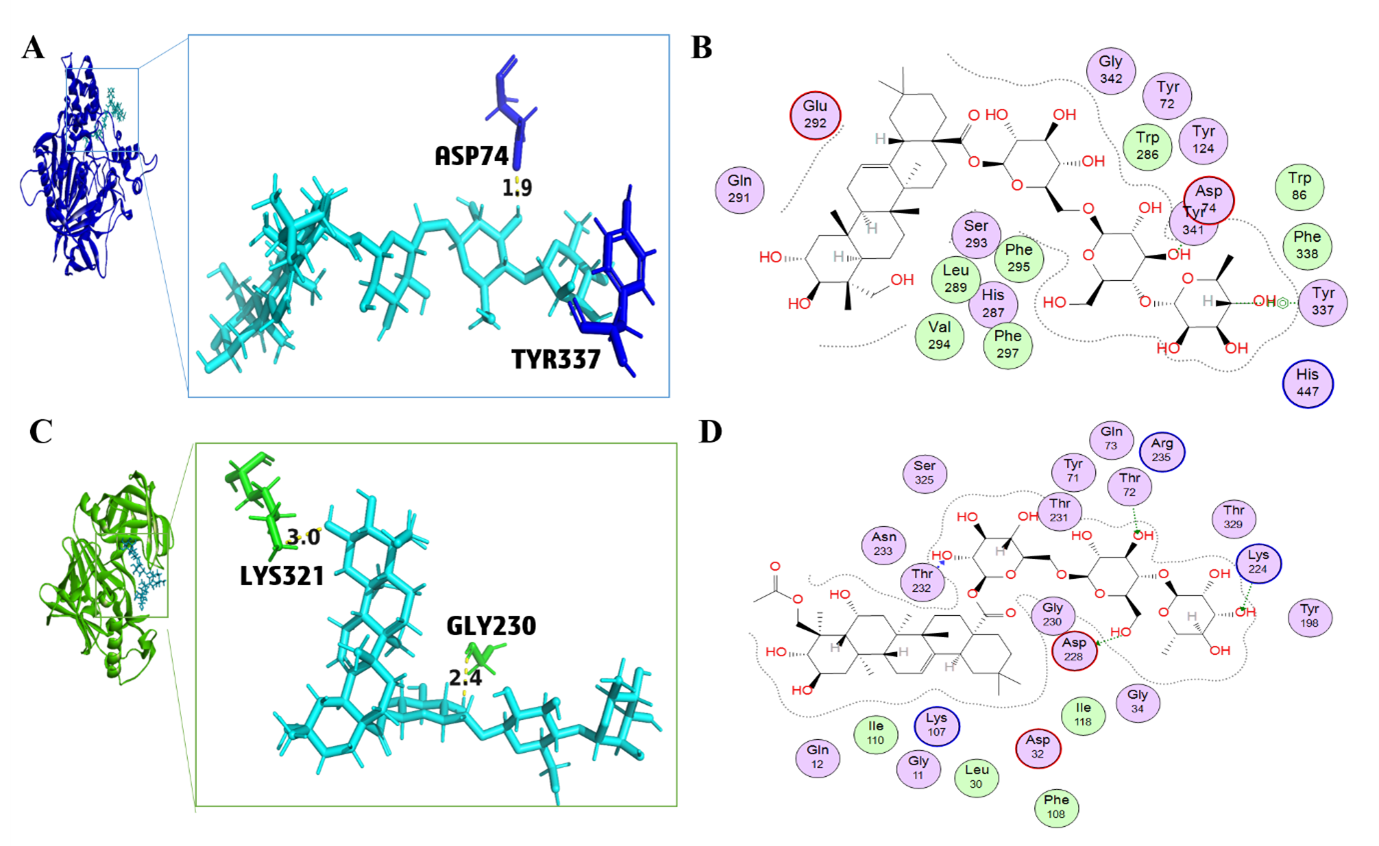

Supplement: S5 Fig — (A) 3D (B) 2D interaction profile of compound 5 with AChE; (C) 3D and (D) 2D interaction profile of compound 5 with BACE1. Residues from AChE are highlighted in blue and residues from BACE1 are highlighted in green. (TIF) [file pone.0325441.s005.tif]

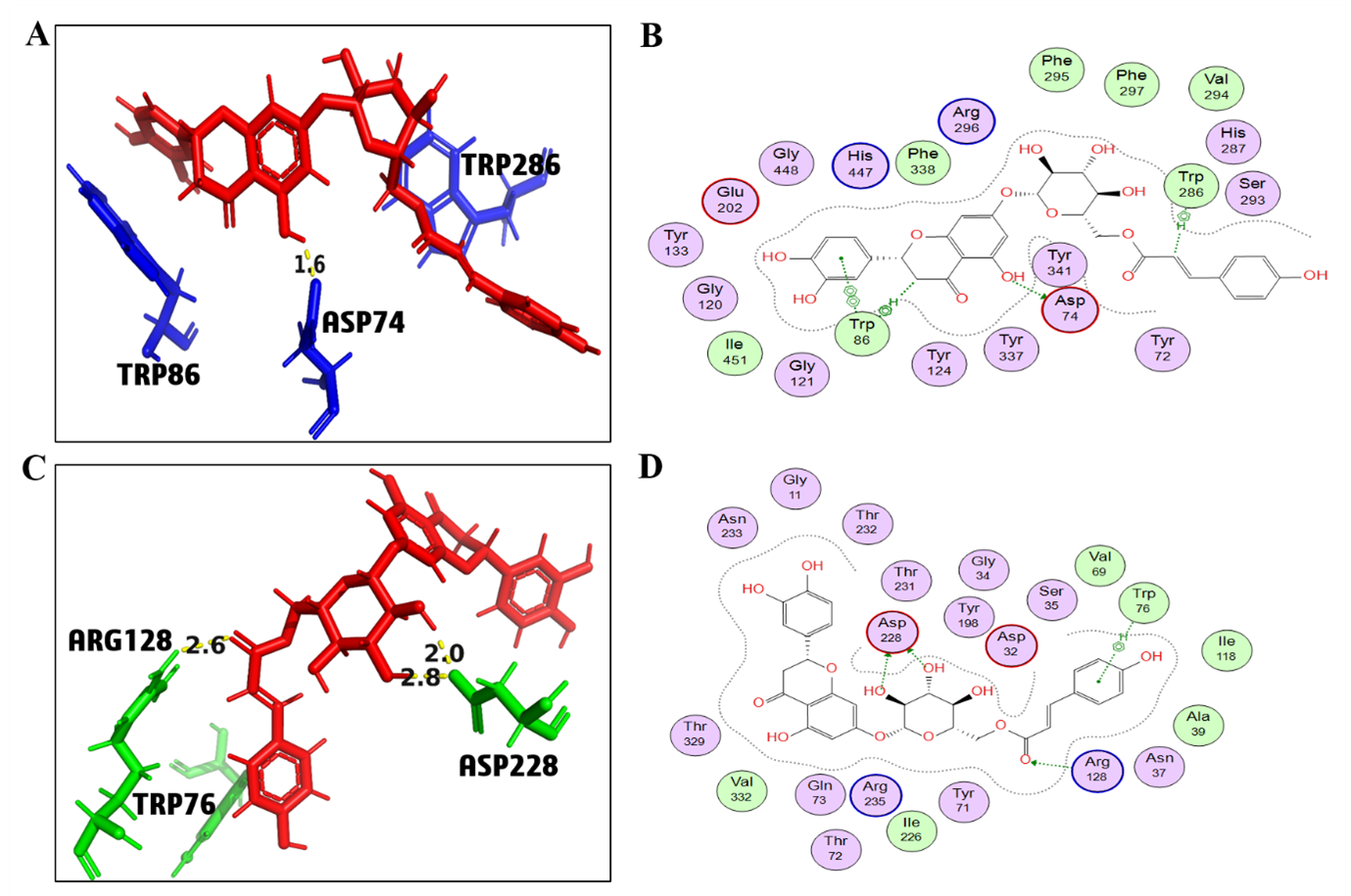

Supplement: S6 Fig — (A) 3D (B) 2D interaction profile of compound 6 with AChE; (C) 3D and (D) 2D interaction profile of compound 6 with BACE1. Residues from AChE are highlighted in blue and residues from BACE1 are highlighted in green. (TIF) [file pone.0325441.s006.tif]

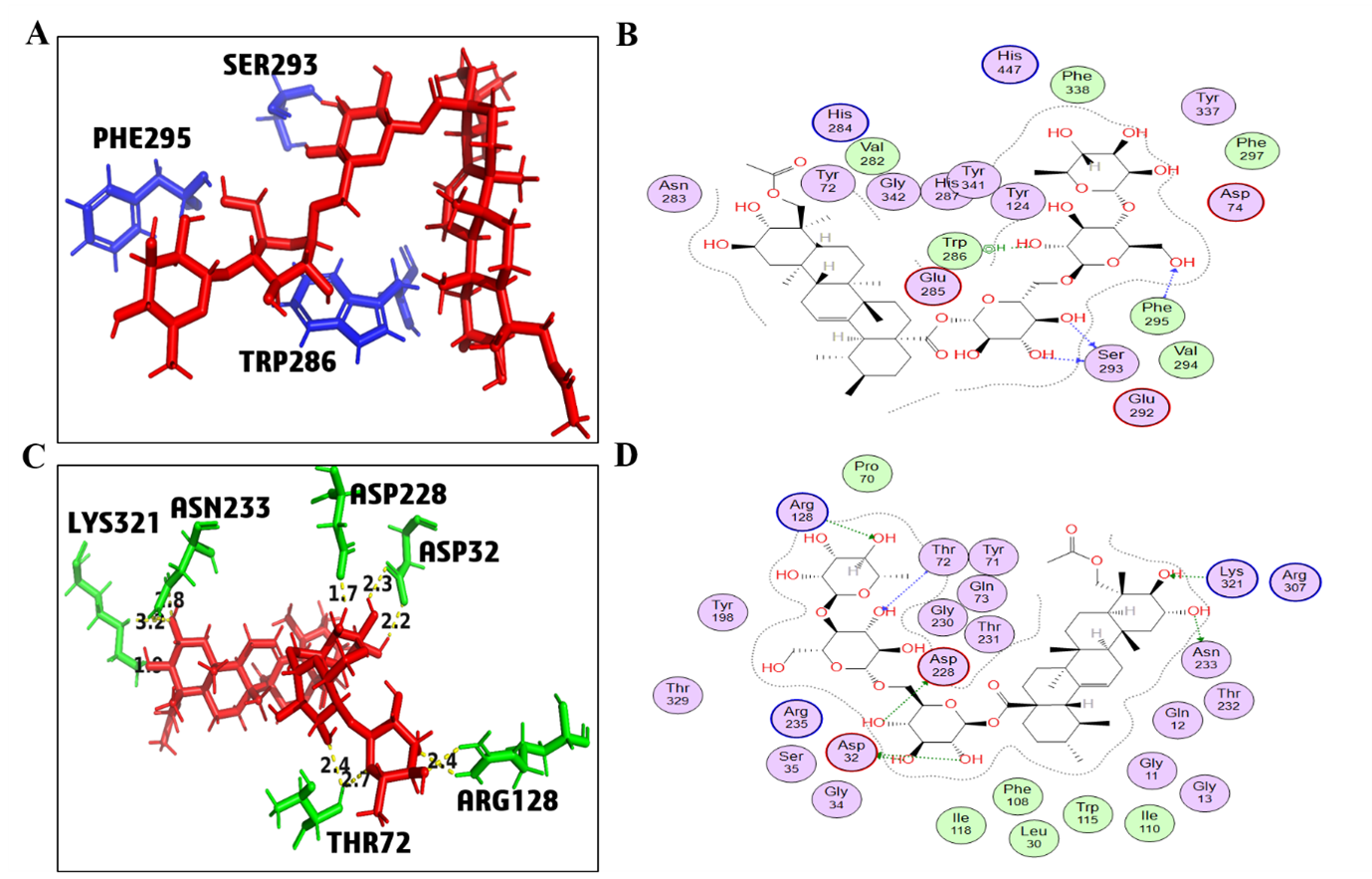

Supplement: S7 Fig — (A) 3D (B) 2D interaction profile of compound 7 with AChE; (C) 3D and (D) 2D interaction profile of compound 7 with BACE1. Residues from AChE are highlighted in blue and residues from BACE1 are highlighted in green. (TIF) [file pone.0325441.s007.tif]

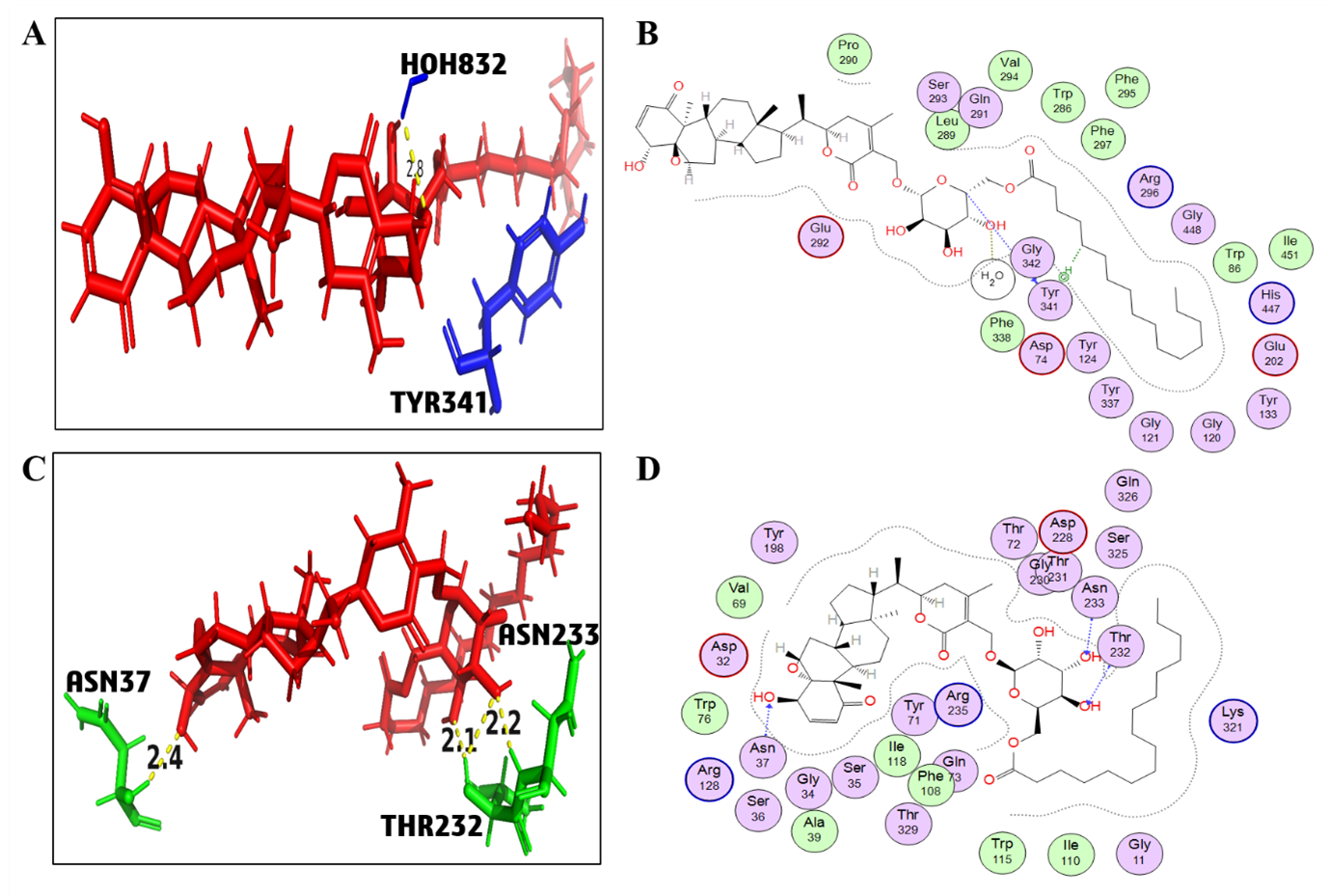

Supplement: S8 Fig — (A) 3D (B) 2D interaction profile of compound 8 with AChE; (C) 3D and (D) 2D interaction profile of compound 8 with BACE1. Residues from AChE are highlighted in blue and residues from BACE1 are highlighted in green. (TIF) [file pone.0325441.s008.tif]

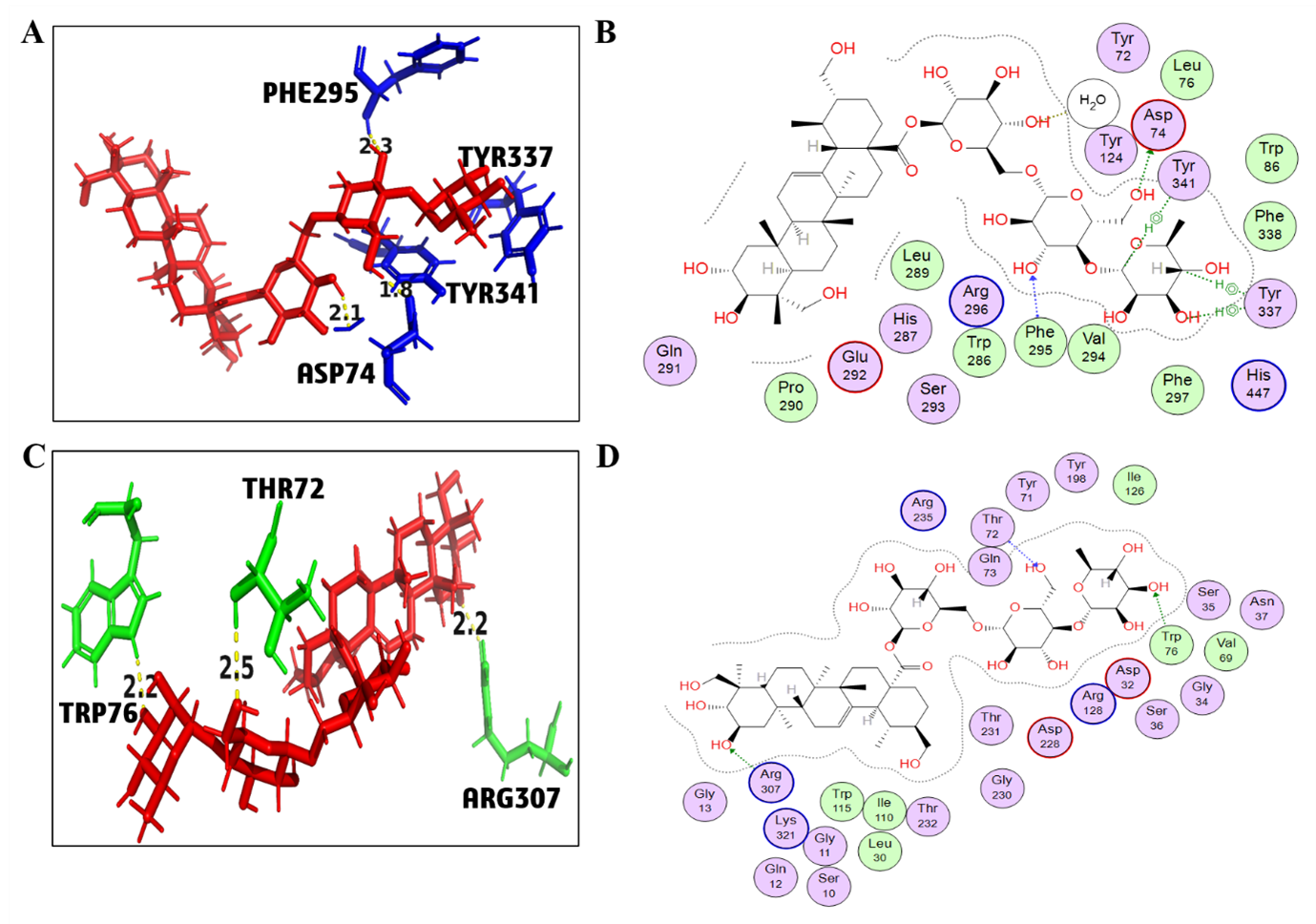

Supplement: S9 Fig — (A) 3D (B) 2D interaction profile of compound 9 with AChE; (C) 3D and (D) 2D interaction profile of compound 9 with BACE1. Residues from AChE are highlighted in blue and residues from BACE1 are highlighted in green. (TIF) [file pone.0325441.s009.tif]

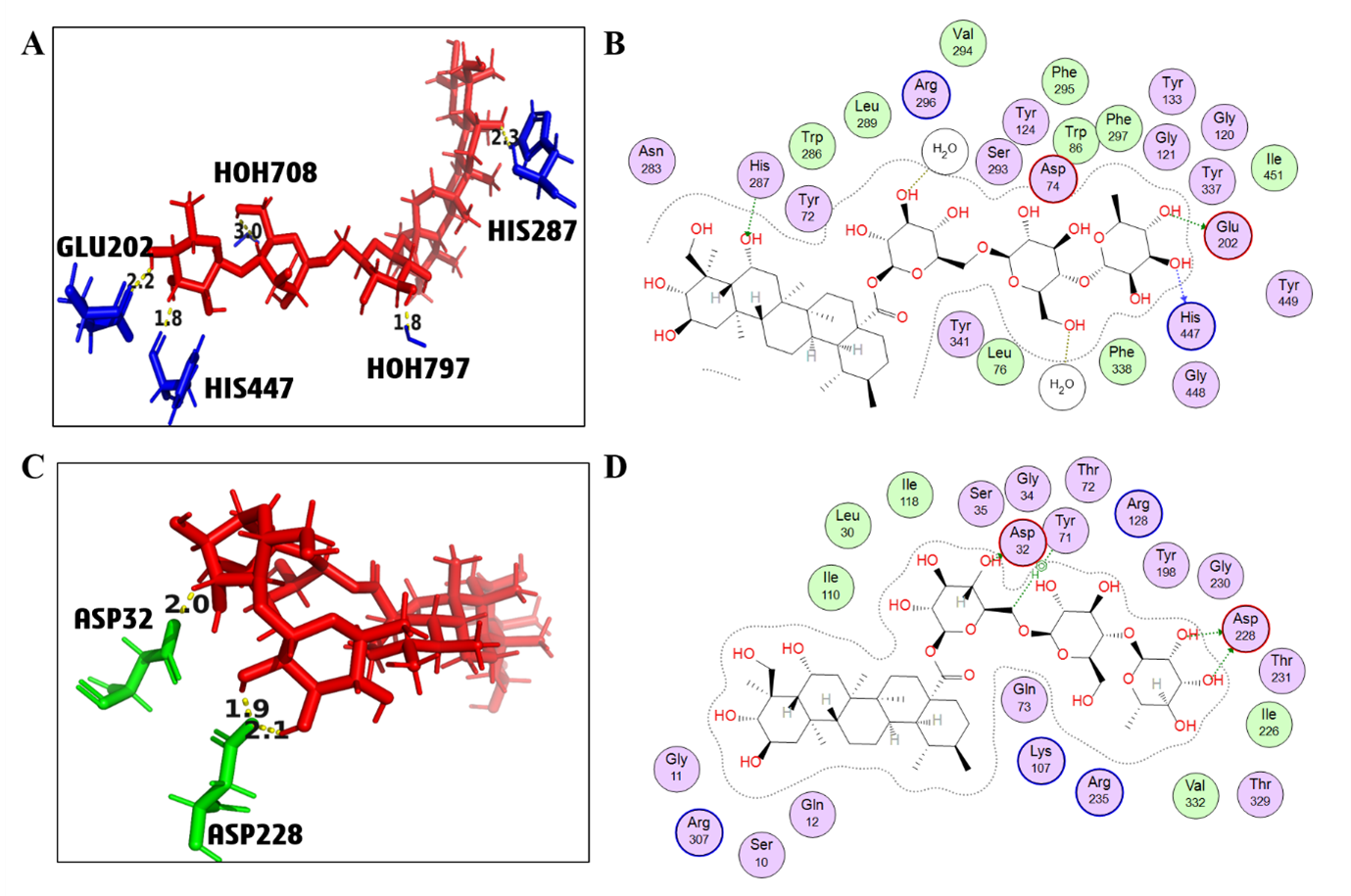

Supplement: S10 Fig — (A) 3D (B) 2D interaction profile of compound 10 with AChE; (C) 3D and (D) 2D interaction profile of compound 10 with BACE1. Residues from AChE are highlighted in blue and residues from BACE1 are highlighted in green. (TIF) [file pone.0325441.s010.tif]

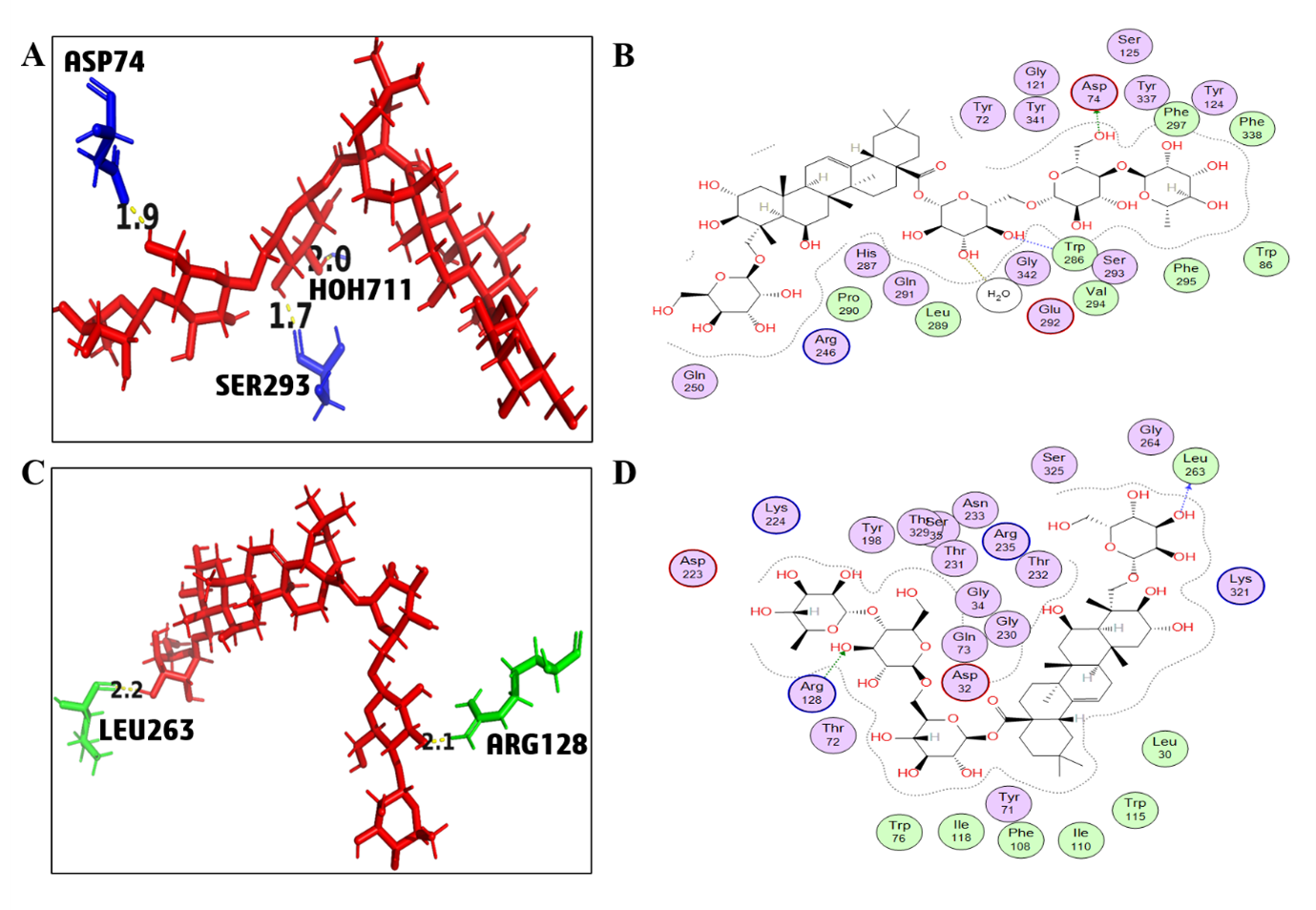

Supplement: S11 Fig — (A) 3D (B) 2D interaction profile of compound 11 with AChE; (C) 3D and (D) 2D interaction profile of compound 11 with BACE1. Residues from AChE are highlighted in blue and residues from BACE1 are highlighted in green. (TIF) [file pone.0325441.s011.tif]

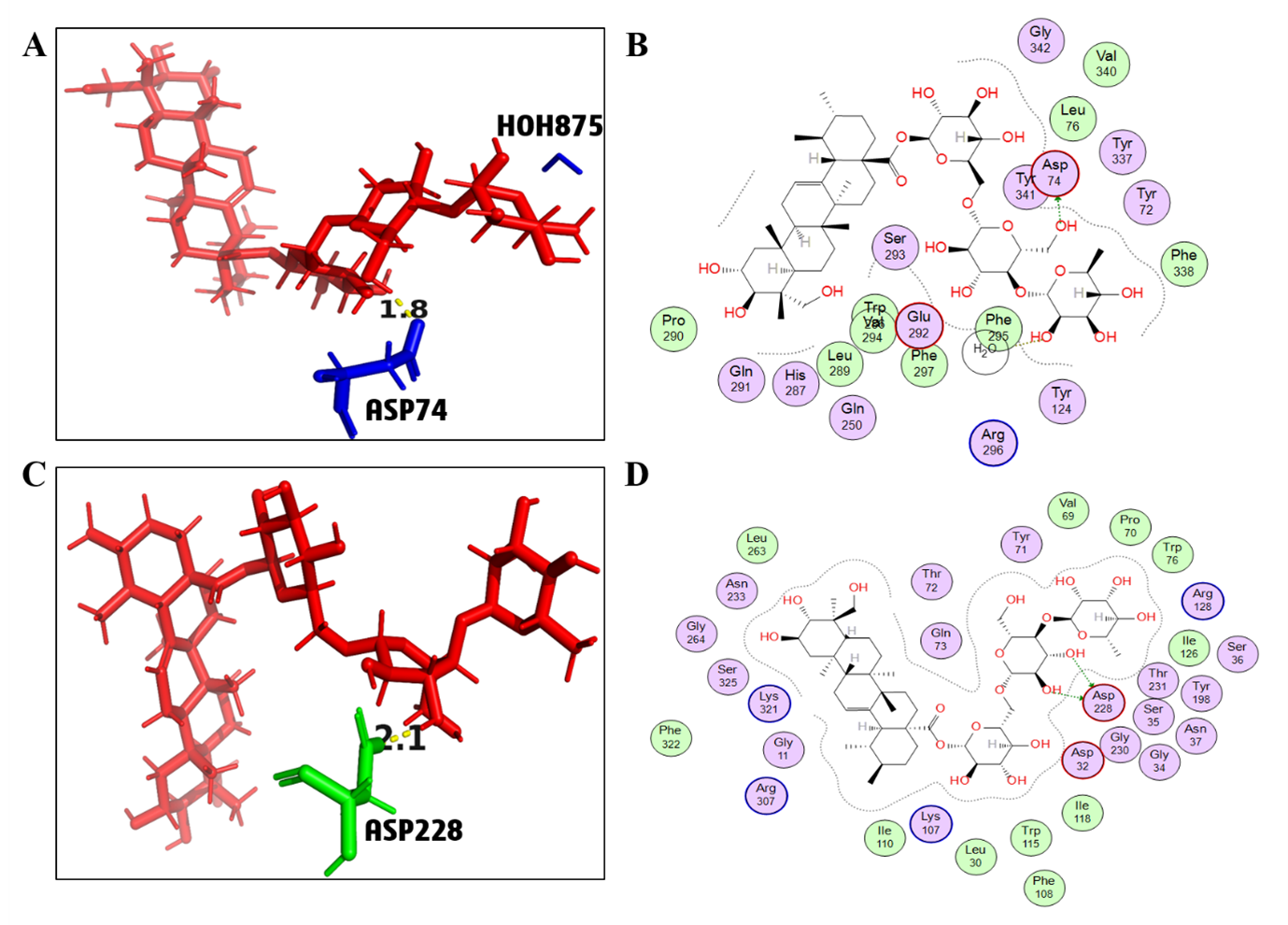

Supplement: S12 Fig — (A) 3D (B) 2D interaction profile of compound 12 with AChE; (C) 3D and (D) 2D interaction profile of compound 12 with BACE1. Residues from AChE are highlighted in blue and residues from BACE1 are highlighted in green. (TIF) [file pone.0325441.s012.tif]

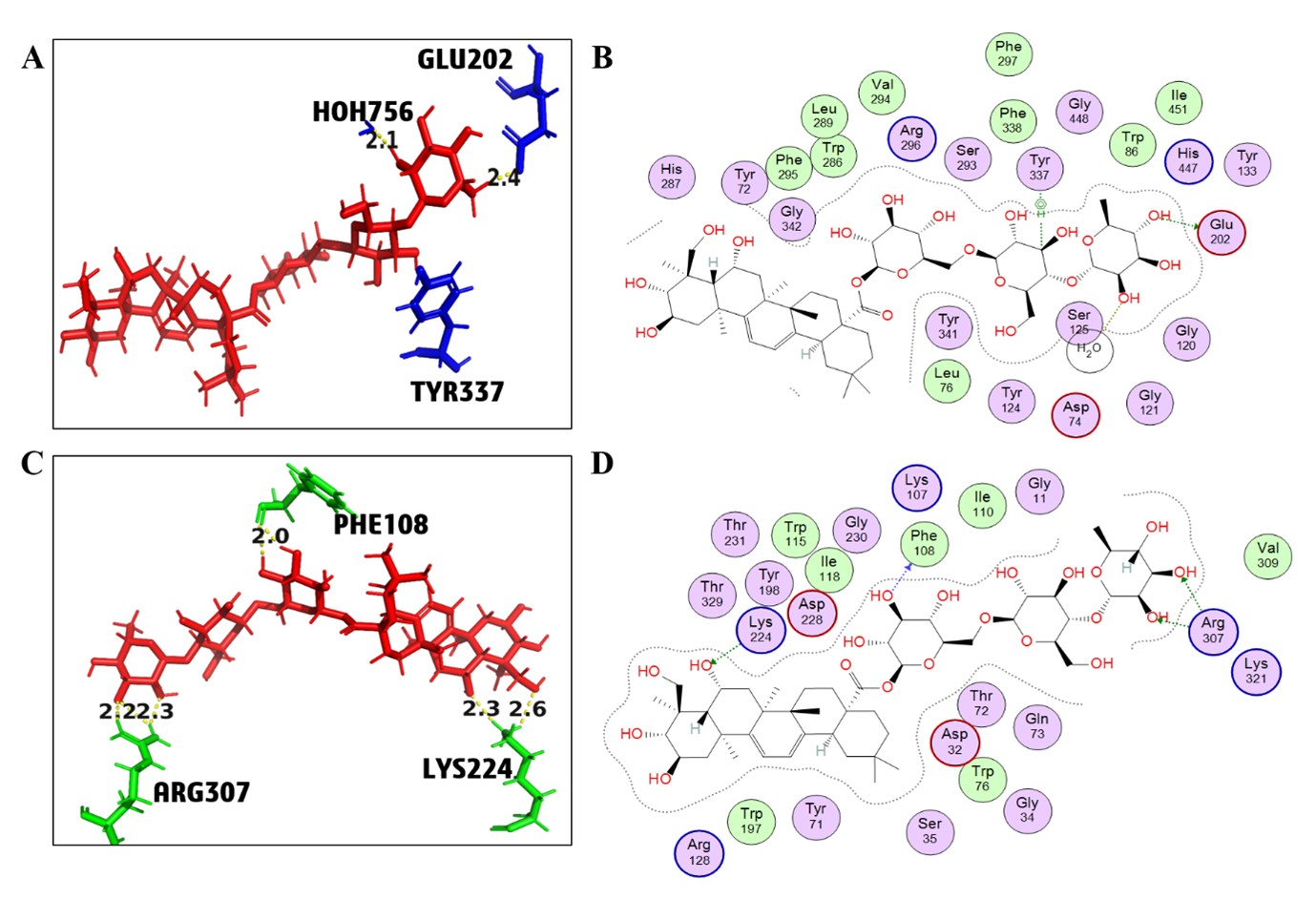

Supplement: S13 Fig — (A) 3D (B) 2D interaction profile of compound 13 with AChE; (C) 3D and (D) 2D interaction profile of compound 13 with BACE1. Residues from AChE are highlighted in blue and residues from BACE1 are highlighted in green. (TIF) [file pone.0325441.s013.tif]

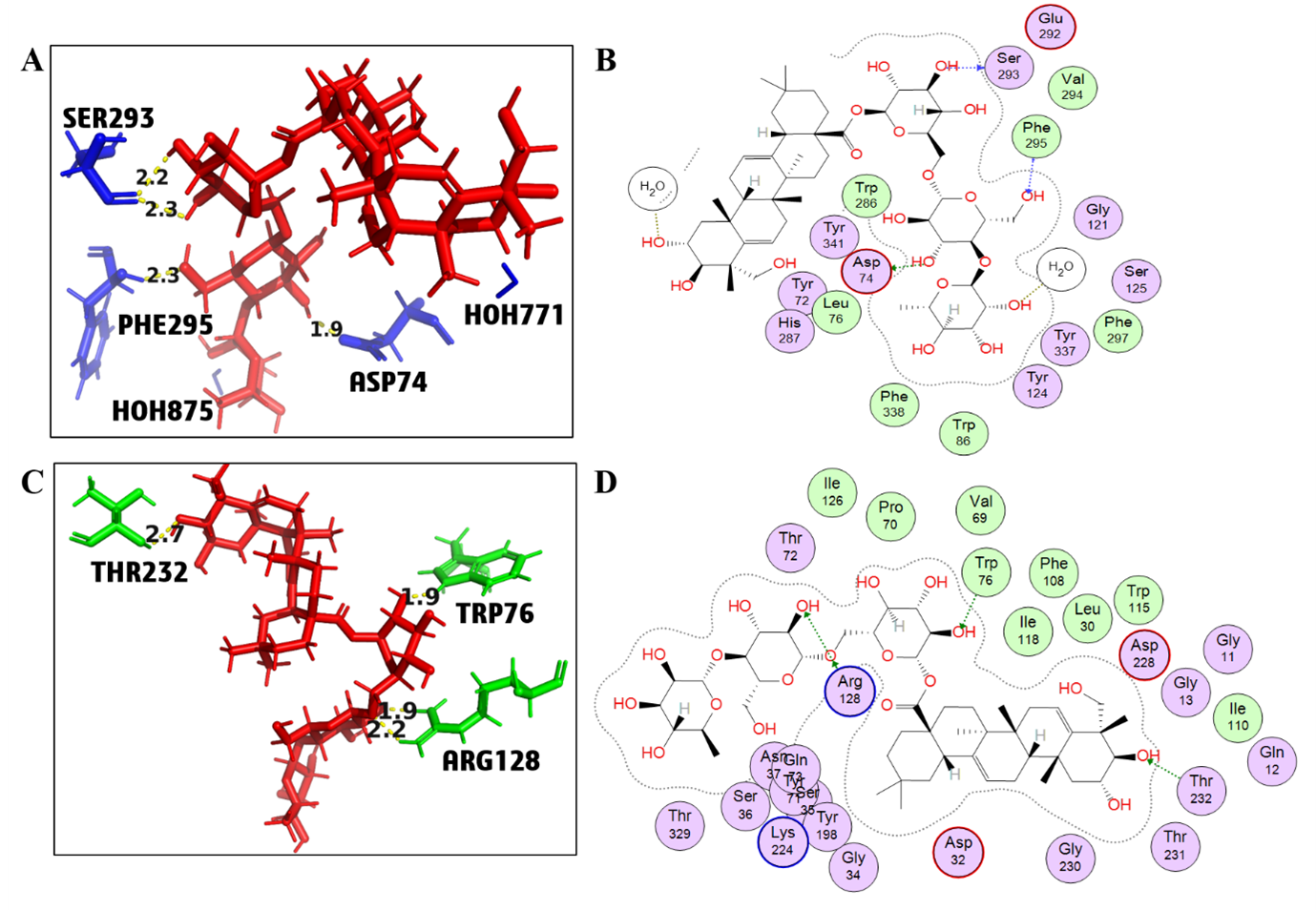

Supplement: S14 Fig — (A) 3D (B) 2D interaction profile of compound 14 with AChE; (C) 3D and (D) 2D interaction profile of compound 14 with BACE1. Residues from AChE are highlighted in blue and residues from BACE1 are highlighted in green. (TIF) [file pone.0325441.s014.tif]

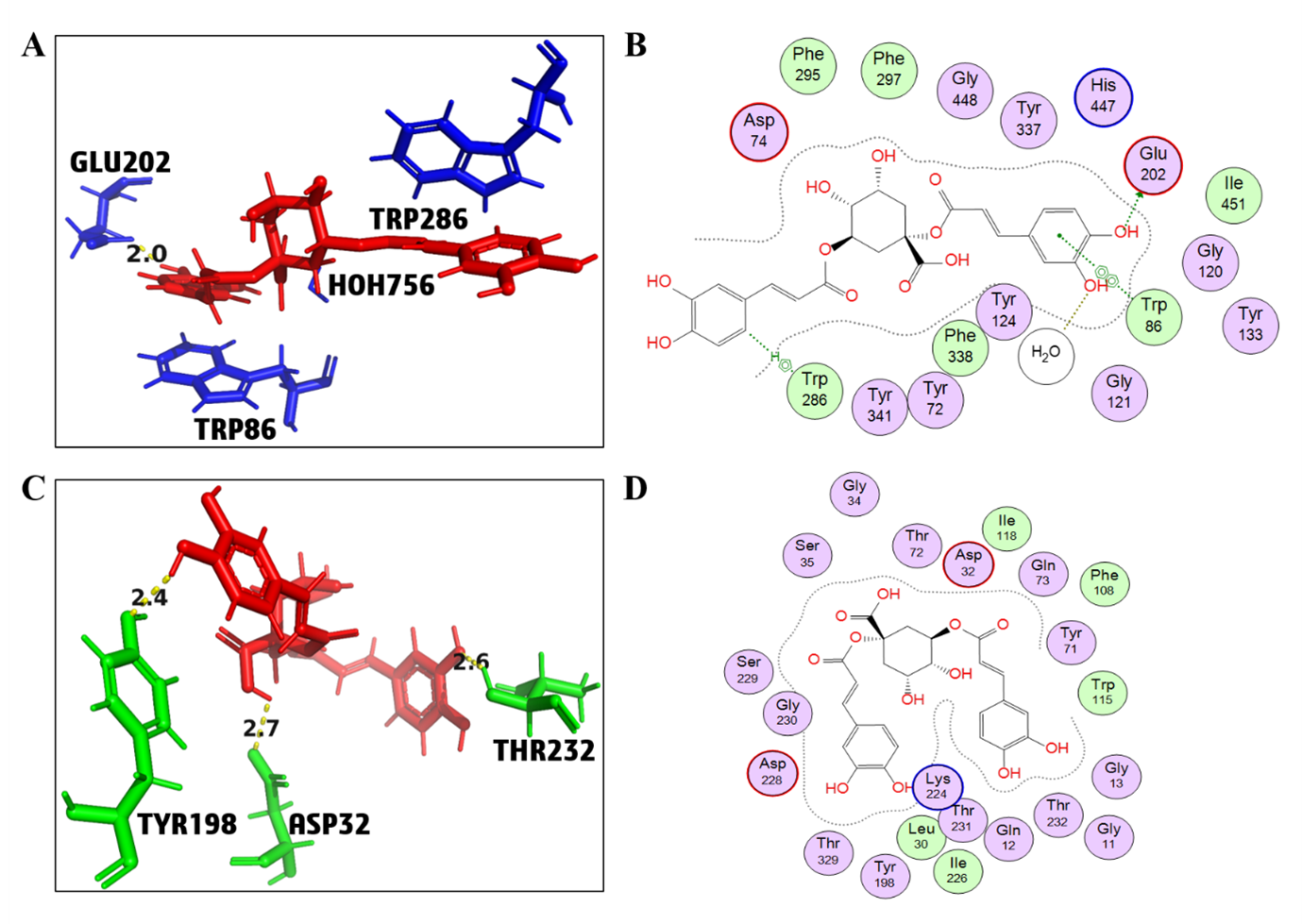

Supplement: S15 Fig — (A) 3D (B) 2D interaction profile of compound 15 with AChE; (C) 3D and (D) 2D interaction profile of compound 15 with BACE1. Residues from AChE are highlighted in blue and residues from BACE1 are highlighted in green. (TIF) [file pone.0325441.s015.tif]
