# Supplementary material for: Computer-aided discovery of dual-target compounds for Alzheimer’s from ayurvedic medicinal plants
Source: PLoS One. 2025 Jun 25;20(6):e0325441. doi: 10.1371/journal.pone.0325441 (PMC12193798; doi:10.1371/journal.pone.0325441)
Supplement: S1 Table — The docking scores of these phytochemicals are mentioned with acetylcholinesterase (AChE) and β-secretase (BACE1). (DOCX) [file pone.0325441.s016.docx]

**S1 Table.** Library of phytoconstituents obtained from selected ayurvedic plants. The docking scores of these phytochemicals are mentioned with acetylcholinesterase (AChE) and β-secretase (BACE1).

| **Sr. No.** | **Name** | **Structure** | ***Plant*** | **Class of compound** | **AChE**  **score** | **BACE1 score** | **Ref** |
| --- | --- | --- | --- | --- | --- | --- | --- |
|  | **Donepezil** | **** |  | **Piperdine derivative** | **-10.34** | **-** |  |
|  | **Elenbecestat** |  |  | **Synthetic organic** | **-** | **-6.22** |  |
| 1 | α-Asarone |  | *Acorus calamus* | Phenylpropanoid | -6.22 | -4.18 | [47] |
| 2 | β-Asarone |  | *Acorus calamus* | Phenylpropanoid | -5.91 | -4.64 | [47] |
| 3 | Gamma-Asarone |  | *Acorus calamus* | Phenylpropanoid | -6.01 | -4.31 | [47] |
| 4 | Eugenyl acetate |  | *Acorus calamus* | Phenylpropanoid | -6.40 | -4.25 | [47] |
| 5 | Eugenol |  | *Acorus calamus* | Phenylpropanoid | -5.94 | -4.42 | [47] |
| 6 | Isoeugenol |  | *Acorus calamus* | Phenylpropanoid | -5.61 | -4.20 | [47] |
| 7 | Methy eugenol |  | *Acorus calamus* | Phenylpropanoid | -5.46 | -4.21 | [47] |
| 8 | Methyl isoeugenol |  | *Acorus calamus* | Phenylpropanoid | -6.08 | -4.14 | [47] |
| 9 | Calamol |  | *Acorus calamus* | Phenylpropanoid | -5.45 | -4.08 | [47] |
| 10 | Azulene |  | *Acorus calamus* | Phenylpropanoid | -5.25 | -3.55 | [47] |
| 11 | Eugenol methyl ether |  | *Acorus calamus* | Phenylpropanoid | -5.22 | -4.30 | [47] |
| 12 | Limolene(dipentene) |  | *Acorus calamus* | Phenylpropanoid | -4.71 | -3.68 | [47] |
| 13 | Asoronaldehyde |  | *Acorus calamus* | Phenylpropanoid | -5.46 | -3.94 | [47] |
| 14 | Terpinolene |  | *Acorus calamus* | Phenylpropanoids | -5.40 | -3.75 | [47] |
| 15 | 1,8-ciol(Eucalyptol) |  | *Acorus calamus* | Phenylpropanoids | -4.65 | -3.48 | [47] |
| 16 | E-isoeugenol acetate |  | *Acorus calamus* | Phenylpropanoids | -6.69 | -4.44 | [47] |
| 17 | E-methyl isoeugenol |  | *Acorus calamus* | Phenylpropanoids | -6.08 | -4.15 | [47] |
| 18 | Cis-methyl isoeugenol |  | *Acorus calamus* | Phenylpropanoids | -6.07 | -4.22 | [47] |
| 19 | Euasarone |  | *Acorus calamus* | Phenylpropanoids | -5.89 | -4.36 | [47] |
| 20 | Cinnamaldehyde |  | *Acorus calamus* | Phenylpropanoids | -4.57 | -3.74 | [47] |
| 21 | Cyclohexanone |  | *Acorus calamus* | Phenylpropanoids | -4.23 | -3.11 | [47] |
| 22- | Acorin |  | *Acorus calamus* | Phenylpropanoids | -9.07 | **-7.66** | [47] |
| 23 | Safrole |  | *Acorus calamus* | Phenylpropanoids | -5.43 | -3.63 | [47] |
| 24 | Z-3-(2,4,5-trimethoxyphenyl)-2-propenal |  | *Acorus calamus* | Phenylpropanoids | - | - | [47] |
| 25 | 2,3-dihydro-4,5,7-trimethoxy-1-ethyl-2-methyl-3 (2,4,5-trimethoxyphenyl)indene |  | *Acorus calamus* | Phenylpropanoids | -6.28 | -4.48 | [47] |
| 26 | (E)-caryophyllene |  | *Acorus calamus* | Phenylpropanoids | -4.92 | -4.25 | [47] |
| 27 | Estragole |  | *Acorus calamus* | Phenylpropanoids | -5.06 | -3.90 | [47] |
| 28 | Carvacrol |  | *Acorus calamus* | Phenylpropanoids | -5.87 | -4.38 | [47] |
| 29 | 2-cyclohexane-1-one |  | *Acorus calamus* | Phenylpropanoids | -4.03 | -2.87 | [47] |
| 30 | Naphthalene |  | *Acorus calamus* | Phenylpropanoids | -4.95 | -3.49 | [47] |
| 31 | γ-Cadinene |  | *Acorus calamus* | Phenylpropanoids | -5.06 | -4.40 | [47] |
| 32 | Aristolene |  | *Acorus calamus* | Phenylpropanoids | - | - | [47] |
| 33 | 1(5),3-aromadenedradiene |  | *Acorus calamus* | Phenylpropanoids | -5.19 | -3.97 | [47] |
| 34 | 5-n-butyltetraline |  | *Acorus calamus* | Phenylpropanoids | -5.57 | -4.21 | [47] |
| 35 | 4,5-dehydroisolongifolene |  | *Acorus calamus* | Phenylpropanoids | -3.85 | -4.26 | [47] |
| 36 | Calarene |  | *Acorus calamus* | Phenylpropanoids | -3.46 | -4.10 | [47] |
| 37 | Isohomogenol |  | *Acorus calamus* | Phenylpropanoids | -6.08 | -4.14 | [47] |
| 38 | Zingiberene |  | *Acorus calamus* | Phenylpropanoids | -6.23 | -4.32 | [47] |
| 39 | α-Calacorene |  | *Acorus calamus* | Phenylpropanoids | -5.15 | -4.48 | [47] |
| 40 | 5,8-dimethyl isoquinoline |  | *Acorus calamus* | Phenylpropanoids | -5.04 | -3.90 | [47] |
| 41 | Cyclohexane methanol |  | *Acorus calamus* | Phenylpropanoids | -4.56 | -3.51 | [47] |
| 42 | Longifolene |  | *Acorus calamus* | Phenylpropanoids | -4.41 | -4.26 | [47] |
| 43 | Isoelemicin |  | *Acorus calamus* | Phenylpropanoids | -5.53 | -4.49 | [47] |
| 44 | Calamene |  | *Acorus calamus* | Sesquiterpenoids | -5.66 | -4.18 | [47] |
| 45 | Calamenenol |  | *Acorus calamus* | Sesquiterpenoids | -5.70 | -4.40 | [47] |
| 46 | Calamoene |  | *Acorus calamus* | Sesquiterpenoids | -5.06 | -4.50 | [47] |
| 47 | Preisocalamendiol |  | *Acorus calamus* | Sesquiterpenoids | -6.35 | -5.06 | [47] |
| 48 | 1,4-(trans)1,7(trans)-acorenone |  | *Acorus calamus* | Sesquiterpenoids | -6.08 | -4.28 | [47] |
| 49 | 1,4-(cis)-1,7-(trans)-acorenone |  | *Acorus calamus* | Sesquiterpenoids | -4.15 | -4.37 | [47] |
| 50 | 2,6 diepishyobunone |  | *Acorus calamus* | Sesquiterpenoids | -5.28 | -4.18 | [47] |
| 51 | α-Gurjunene |  | *Acorus calamus* | Sesquiterpenoids | -4.99 | -3.99 | [47] |
| 52 | β-Gurjunene |  | *Acorus calamus* | Sesquiterpenoids | -4.78 | -4.08 | [47] |
| 53 | α-Cedrene |  | *Acorus calamus* | Sesquiterpenoids | -4.49 | -3.97 | [47] |
| 54 | β-Elemene |  | *Acorus calamus* | Sesquiterpenoids | -5.29 | -4.48 | [47] |
| 55 | β-Cedrene |  | *Acorus calamus* | Sesquiterpenoids | -5.02 | -3.97 | [47] |
| 56 | β-Caryophyllene |  | *Acorus calamus* | Sesquiterpenoids | -4.92 | -4.25 | [47] |
| 57 | Valencene |  | *Acorus calamus* | Sesquiterpenoids | -5.03 | -4.06 | [47] |
| 58 | Viridiflorene |  | *Acorus calamus* | Sesquiterpenoids | -5.10 | -4.28 | [47] |
| 59 | α-Selinene |  | *Acorus calamus* | Sesquiterpenoids | -5.36 | -4.12 | [47] |
| 60 | δ-Cadinene |  | *Acorus calamus* | Sesquiterpenoids | -5.29 | -4.59 | [47] |
| 61 | α-Curcumene |  | *Acorus calamus* | Sesquiterpenoids | -6.51 | -4.69 | [47] |
| 62 | Shyobunone |  | *Acorus calamus* | Sesquiterpenoids | -5.68 | -4.20 | [47] |
| 63 | Isoshyobunone |  | *Acorus calamus* | Sesquiterpenoids | -5.96 | -4.02 | [47] |
| 64 | Caryophyllene oxide |  | *Acorus calamus* | Sesquiterpenoids | -4.48 | -4.15 | [47] |
| 65 | Humulene oxide II |  | *Acorus calamus* | Sesquiterpenoids | -5.11 | -4.61 | [47] |
| 66 | Elemol |  | *Acorus calamus* | Sesquiterpenoids | -5.71 | -4.66 | [47] |
| 67 | Cedrol |  | *Acorus calamus* | Sesquiterpenoids | -3.95 | -4.11 | [47] |
| 68 | Spathulenol |  | *Acorus calamus* | Sesquiterpenoids | -5.40 | -4.51 | [47] |
| 69 | Acorenone |  | *Acorus calamus* | Sesquiterpenoids | -4.30 | -4.24 | [47] |
| 70 | α-Cadinol |  | *Acorus calamus* | Sesquiterpenoids | -5.01 | -4.34 | [47] |
| 71 | Humulene epoxide II |  | *Acorus calamus* | Sesquiterpenoids | -4.85 | -4.02 | [47] |
| 72 | α-Bisabolol |  | *Acorus calamus* | Sesquiterpenoids | -7.34 | -4.67 | [47] |
| 73 | Calamusenone |  | *Acorus calamus* | Sesquiterpenoids | -6.24 | -4.31 | [47] |
| 74 | Isocalamendiol |  | *Acorus calamus* | Sesquiterpenoids | -4.81 | -4.84 | [47] |
| 75 | Dehydroxyisocalamendiol |  | *Acorus calamus* | Sesquiterpenoids | -4.88 | -4.10 | [47] |
| 76 | Epishyobunone |  | *Acorus calamus* | Sesquiterpenoids | -4.78 | -4.14 | [47] |
| 77 | Acorone |  | *Acorus calamus* | Sesquiterpenoids | -5.08 | -4.33 | [47] |
| 78 | Neo-acorane A |  | *Acorus calamus* | Sesquiterpenoids | -5.65 | -4.72 | [47] |
| 79 | Acoric acid |  | *Acorus calamus* | Sesquiterpenoids | -5.22 | -4.80 | [47] |
| 80 | Calamusin D |  | *Acorus calamus* | Sesquiterpenoids | -3.52 | -4.61 | [47] |
| 81 | 1β,5α-Guaiane-4β,10α-diol-6-one |  | *Acorus calamus* | Sesquiterpenoids | -5.67 | -4.36 | [47] |
| 82 | Dioxosarcoguaiacol |  | *Acorus calamus* | Sesquiterpenoids | -6.27 | -4.31 | [47] |
| 83 | 7-tetracycloundecanol,4,4,11,11-tetramethyl |  | *Acorus calamus* | Sesquiterpenoids | - | - | [47] |
| 84 | 4α,7-Methano-4α-naphth[1 ,8a-b] oxirene |  | *Acorus calamus* | Sesquiterpenoids | -3.35 | -4.57 | [47] |
| 85 | Vulgarol B |  | *Acorus calamus* | Sesquiterpenoids | -5.33 | -4.54 | [47] |
| 86 | Tatanan A |  | *Acorus calamus* | Sesquiterpenoid | -4.52 | -3.88 | [47] |
| 87 | Acoramone |  | *Acorus calamus* | Sesquiterpenoid | -6.13 | -4.04 | [47] |
| 88 | 2-hydroxyacorenone |  | *Acorus calamus* | Sesquiterpenoid | -5.99 | -4.72 | [47] |
| 89 | 4-(2-formyl-5-methoxymethyl pyrrol-1-yl) butyric acid methyl ester |  | *Acorus calamus* | Sesquiterpenoid | -6.51 | -4.81 | [47] |
| 90 | 2-acetoxyacorenone |  | *Acorus calamus* | Sesquiterpenoid | -5.93 | -4.75 | [47] |
| 91 | Acoramol |  | *Acorus calamus* | Sesquiterpenoid | -6.50 | -4.36 | [47] |
| 92 | N-transferuloyl  tyramine |  | *Acorus calamus* | Sesquiterpenoid | -7.88 | -5.35 | [47] |
| 93 | Tatarinoid A |  | *Acorus calamus* | Sesquiterpenoid | -6.38 | -4.52 | [47] |
| 94 | Tatarinoid B |  | *Acorus calamus* | Sesquiterpenoid | -5.77 | -4.58 | [47] |
| 95 | Acortatarin A |  | *Acorus calamus* | Sesquiterpenoid | -5.70 | -4.03 | [47] |
| 96 | α-Pinene |  | *Acorus calamus* | Monoterpenes | -4.52 | -3.58 | [47] |
| 97 | β-Pinene |  | *Acorus calamus* | Monoterpenes | -4.45 | -3.50 | [47] |
| 98 | Camphene |  | *Acorus calamus* | Monoterpenes | -4.09 | -3.75 | [47] |
| 99 | o-Cymol |  | *Acorus calamus* | Monoterpenes | -5.10 | -3.63 | [47] |
| 100 | p-Cymene |  | *Acorus calamus* | Monoterpenes | -5.04 | -3.73 | [47] |
| 101 | γ-Terpinene |  | *Acorus calamus* | Monoterpenes | -5.08 | -4.70 | [47] |
| 102 | Anethole |  | *Acorus calamus* | Monoterpenes | -5.49 | -4.85 | [47] |
| 103 | Thymol |  | *Acorus calamus* | Monoterpenes | -5.63 | -4.65 | [47] |
| 104 | Isoaromadendrene  epoxide |  | *Acorus calamus* | Monoterpenes | -5.53 | -4.47 | [47] |
| 105 | CAMPHOR |  | *Acorus calamus* | Monoterpenes | -4.59 | -3.62 | [47] |
| 106 | Sabinene |  | *Acorus calamus* | Monoterpenes | -4.96 | -4.27 | [47] |
| 107 | 2-hexenal |  | *Acorus calamus* | Monoterpenes | -4.10 | -3.74 | [47] |
| 108 | Limonene |  | *Acorus calamus* | Monoterpenes | -5.48 | -4.38 | [47] |
| 109 | Cis-linaloloxide |  | *Acorus calamus* | Monoterpenes | -6.31 | -4.59 | [47] |
| 110 | Cis-sabinene hydrate |  | *Acorus calamus* | Monoterpenes | - | - | [47] |
| 111 | Trans-linalol oxide |  | *Acorus calamus* | Monoterpenes | -5.46 | -4.70 | [47] |
| 112 | Linalool |  | *Acorus calamus* | Monoterpenes | -5.73 | -4.64 | [47] |
| 113 | Terpinen-4-ol |  | *Acorus calamus* | Monoterpenes | -5.71 | -4.83 | [47] |
| 114 | α-Acoradiene |  | *Acorus calamus* | Monoterpenes | -6.28 | -5.38 | [47] |
| 115 | B-Acoradiene |  | *Acorus calamus* | Monoterpenes | -5.46 | -4.55 | [47] |
| 116 | α-Terpineol |  | *Acorus calamus* | Monoterpenes | -5.31 | -4.41 | [47] |
| 117 | Isoborneol |  | *Acorus calamus* | Monoterpenes | - | - | [47] |
| 118 | 4,5,8-trimethoxyxanthone-2-O-β-Dglucopyranosyl (1-2)-O-β-Dgalactopyranoside |  | *Acorus calamus* | Xanthone glycosides | -8.12 | **-8.59** | [47] |
| 119 | 1β,2α,3β, 19α-Tetrahydroxyurs-12-en-28-oic acid-28-O- {(β-D-glucopyranosyl (1-2)}-β-D galactopyranoside |  | *Acorus calamus* | Triterpenoid  saponins | - | - | [47] |
| 120 | 3-β, 22-α-24,29-Tetrahydroxyolean-12-en-3-O-(β-Darabinosyl (1,3)}-β-D-arabinopyranoside |  | *Acorus calamus* | Triterpenoid  saponins | -7.59 | **-8.59** | [47] |
| 121 | Trimethoxyampheta  mine,2,3,5 |  | *Acorus calamus* | Alkaloid | -5.89 | -4.74 | [47] |
| 122 | Pyrimidin-2-one,4-[N-methylureido]-1-[4methyl amino carbonloxy methy] |  | *Acorus calamus* | Alkaloid | -7.20 | **-6.57** | [47] |
| 123 | 22-[(6-deoxy-α-L-rhamnopyranosyl) oxy]-3,23-dihydroxy-, methyl ester, (3β,4β,20α,22β) |  | *Acorus calamus* | Triterpene glycoside | -6.25 | **-7.23** | [47] |
| 124 | β-daucosterol |  | *Acorus calamus* | Steroid | - | - | [47] |
| 125 | Nicotine |  | *Bacopa monnieri* | Alkaliod | -6.03 | -4.87 | [48] |
| 126 | D-mannitol |  | *Bacopa monnieri* | Alcohol | -5.66 | -4.68 | [48] |
| 127 | Bacoside A |  | *Bacopa monnieri* | Triterpanoids  saponin | -7.92 | **-8.40** | [48] |
| 128 | Bacopasaponin A |  | *Bacopa monnieri* | Triterpanoids  saponin | - | - | [48] |
| 129 | Bacopasaponin B |  | *Bacopa monnieri* | Triterpanoids  saponin | -5.89 | **-8.41** | [48] |
| 130 | Bacopasaponin C |  | *Bacopa monnieri* | Triterpanoids  saponin | -6.83 | **-9.93** | [48] |
| 131 | Bacopasaponin D |  | *Bacopa monnieri* | Triterpanoids  saponin | - | - | [48] |
| 132 | Bacopasaponin E |  | *Bacopa monnieri* | Triterpanoids  saponin | -6.70 | **-9.22** | [48] |
| 133 | Bacopasaponin F |  | *Bacopa monnieri* | Triterpanoids  saponin | -6.54 | **-9.48** | [48] |
| 134 | Bacopasaponin G |  | *Bacopa monnieri* | Triterpanoids  saponin | -7.42 | **-8.46** | [48] |
| 135 | Bacopaside I |  | *Bacopa monnieri* | Triterpanoids  saponin | -8.95 | **-9.21** | [48] |
| 136 | Bacopaside II |  | *Bacopa monnieri* | Triterpanoids  saponin | -7.90 | **-8.78** | [48] |
| 137 | Bacopaside III |  | *Bacopa monnieri* | Triterpanoids  saponin | -7.95 | **-8.91** | [48] |
| 138 | Bacopaside IV |  | *Bacopa monnieri* | Triterpanoids  saponin | -8.30 | **-8.28** | [48] |
| 139 | Bacopaside V |  | *Bacopa monnieri* | triterpenoid glycoside | -8.48 | **-8.56** | [48] |
| 140 | Bacopaside VI |  | *Bacopa monnieri* | Triterpanoids  saponin | -9.90 | **-8.26** | [48] |
| 141 | Bacopaside VII |  | *Bacopa monnieri* | Triterpanoids  saponin | -5.67 | **-9.31** | [48] |
| 142 | Bacopaside VIII |  | *Bacopa monnieri* | Triterpanoids  saponin | -7.76 | **-9.19** | [48] |
| 143 | Bacopaside XII |  | *Bacopa monnieri* | Triterpanoids  saponin | -1.02 | **-9.95** | [48] |
| 144 | Plantainoside B |  | *Bacopa monnieri* | Sesquiterpenoid | -9.54 | **-7.62** | [48] |
| 145 | Betulinic acid |  | *Bacopa monnieri* | Sriterpenoid | -1.31 | **-6.65** | [48] |
| 146 | Cucurbitacin A |  | *Bacopa monnieri* | Triterpenoid | -6.75 | **-7.18** | [48] |
| 147 | Cucurbitacin B |  | *Bacopa monnieri* | Triterpenoid | -6.43 | **-6.95** | [48] |
| 148 | Cucurbitacin C |  | *Bacopa monnieri* | Triterpenoid | -6.68 | **-7.97** | [48] |
| 149 | Cucurbitacin D |  | *Bacopa monnieri* | Triterpenoid | -6.16 | **-6.45** | [48] |
| 150 | Cucurbitacin E |  | *Bacopa monnieri* | Triterpenoid | -4.84 | **-7.59** | [48] |
| 151 | 3,4Dimethoxycinnamic acid |  | *Bacopa monnieri* | Triterpenoid | -7.68 | -5.84 | [48] |
| 152 | Ascorbic acid |  | *Bacopa monnieri* | Triterpenoid | -5.52 | -4.09 | [48] |
| 153 | Asiatic acid |  | *Bacopa monnieri* | Triterpenoid | -2.87 | **-6.58** | [48] |
| 154 | Rosavin |  | *Bacopa monnieri* | Cinnamyl alcohol glycoside | -8.40 | **-6.50** | [48] |
| 155 | Brahmic acid |  | *Bacopa monnieri* | Triterpenoid | -4.28 | **-6.41** | [48] |
| 156 | Wogonin |  | *Bacopa monnieri* | Flavanoid | -7.20 | -5.27 | [48] |
| 157 | Oroxindin |  | *Bacopa monnieri* | Flavanoid | -8.97 | **-7.08** | [48] |
| 158 | Loliolide |  | *Bacopa monnieri* | Benzofuran | -5.15 | -4.12 | [48] |
| 159 | Stigmasterol |  | *Bacopa monnieri* | Sterol | -6.73 | **-7.15** | [48] |
| 160 | Ebelin lactone |  | *Bacopa monnieri* | Triterpenoid | -5.68 | **-6.86** | [48] |
| 161 | β-sitosterol |  | *Bacopa monnieri* | Sterol | -6.78 | **-7.12** | [48] |
| 162 | Bacosterol |  | *Bacopa monnieri* | Sterol glycoside | -6.44 | **-6.95** | [48] |
| 163 | Bacosine |  | *Bacopa monnieri* | Triterpenoid | -4.44 | **-6.58** | [48] |
| 164 | Heptacosane |  | *Bacopa monnieri* | Alkane | -9.52 | **-7.40** | [48] |
| 165 | Octacosane |  | *Bacopa monnieri* | Alkane | -9.33 | **-8.02** | [48] |
| 166 | Nonacosane |  | *Bacopa monnieri* | Alkane | -9.10 | **-7.24** | [48] |
| 167 | Triacontane |  | *Bacopa monnieri* | Alkane | -8.94 | **-7.37** | [48] |
| 168 | Hentriacontane |  | *Bacopa monnieri* | Alkane | -10.25 | **-7.80** | [48] |
| 169 | Dotriacontane |  | *Bacopa monnieri* | Alkane | -9.88 | **-8.04** | [48] |
| 170 | Apigenin |  | *Bacopa monnieri* | Flavanoid | -7.07 | -5.14 | [48] |
| 171 | Quercetin |  | *Bacopa monnieri* | Flavanoid | -7.36 | -5.35 | [48] |
| 172 | Ursolic acid |  | *Bacopa monnieri* | triterpenoid | - | - | [48] |
| 173 | Luteolin |  | *Bacopa monnieri* | Flavanoid | -7.12 | -5.21 | [48] |
| 174 | Asiaticoside |  | *Bacopa monnieri* | Triterpenoid | **-10.36** | **-9.85** | [48] |
| 175 | Bacopaside X |  | *Bacopa monnieri* | Triterpenoid saponin | -5.70 | **-9.53** | [48] |
| 176 | Bacopaside A |  | *Bacopa monnieri* | Triterpenoid saponin | -8.42 | -6.04 | [48] |
| 177 | Bacopaside B |  | *Bacopa monnieri* | Triterpenoid saponin | -7.97 | **-7.89** | [48] |
| 178 | Bacopaside C |  | *Bacopa monnieri* | Triterpenoid saponin | -7.35 | **-7.64** | [48] |
| 179 | Bacopaside N1 |  | *Bacopa monnieri* | Triterpenoid saponin | -8.18 | **-8.32** | [48] |
| 180 | Bacopaside N2 |  | *Bacopa monnieri* | Triterpenoid saponin | -7.81 | **-7.94** | [48] |
| 181 | Bcoside A3 |  | *Bacopa monnieri* | Triterpanoids  saponins | -7.34 | **-9.30** | [48] |
| 182 | Bacopaside XI |  | *Bacopa monnieri* | Triterpenoid saponin | -7.98 | **-8.62** | [49] |
| 183 | Bacoside A2 |  | *Bacopa monnieri* | Triterpanoids  saponins | -6.87 | **-8.88** | [49] |
| 184 | Pseudojujubogenin |  | *Bacopa monnieri* | Glycoside | -4.33 | -6.08 | [49] |
| 185 | Luteolin-7-rutinoside |  | *Bacopa monnieri* | Triterpanoids | -9.50 | **-7.70** | [49] |
| 186 | Monnieraside III |  | *Bacopa monnieri* | Triterpanoids | -6.55 | **-6.65** | [33] |
| 187 | 4-O-Caffeoylquinic-Acid |  | *Withania somnifera* | Quinic acids | -8.55 | -5.51 | [35] |
| 188 | Anaferine |  | *Withania somnifera* | Piperidine alkaloid | -6.33 | -4.80 | [35] |
| 189 | Anahygrine |  | *Withania somnifera* | Piperidine alkaloid | -6.52 | -4.58 | [35] |
| 190 | β-Sitosterol |  | *Withania somnifera* | Phytosterols | -7.71 | **-6.68** | [35] |
| 191 | Campesterol |  | *Withania somnifera* | Phytosterols | -8.10 | **-7.40** | [35] |
| 192 | Chlorogenic-Acid |  | *Withania somnifera* | 1-cinnamate ester  2-tannin | -7.53 | -6.16 | [35] |
| 193 | Cuscohygrine |  | *Withania somnifera* | N-alkylpyrrolidine and a pyrrolidine alkaloid. | -7.49 | -5.33 | [35] |
| 194 | Daucosterol |  | *Withania somnifera* | Steroid saponin | -7.93 | **-8.04** | [35] |
| 195 | Dulcitol |  | *Withania somnifera* | Hexitol | -5.92 | -4.14 | [35] |
| 196 | Hydroxyproline |  | *Withania somnifera* | Non-aromatic amina acid | -5.07 | -3.94 | [35] |
| 197 | Isopelletierine |  | *Withania somnifera* | Citraconoyl group. | -5.07 | -4.23 | [35] |
| 198 | Linoleic-Acid |  | *Withania somnifera* | Fatty acid | -7.71 | -5.89 | [35] |
| 199 | Quercetin |  | *Withania somnifera* | flavonoid | -6.82 | -5.44 | [35] |
| 200 | Quinic-Acid |  | *Withania somnifera* | Organooxygen compounds | -5.13 | -4.52 | [35] |
| 201 | Rutin |  | *Withania somnifera* | Flavonol glycoside | -7.15 | **-8.87** | [35] |
| 202 | Scopoletin |  | *Withania somnifera* | Hydroxycoumarin | -5.74 | -4.63 | [35] |
| 203 | Sitoindoside-Ix |  | *Withania somnifera* | Withanolide saponin | -7.61 | **-7.54** | [35] |
| 204 | Somniferine |  | *Withania somnifera* | Alkaloid | -5.03 | **-7.38** | [35] |
| 205 | Stigmasterol |  | *Withania somnifera* | Phytosterol | -6.97 | **-6.91** | [35] |
| 206 | Withacnistin |  | *Withania somnifera* | Steroidal lactone | -5.86 | **-7.19** | [35] |
| 207 | Withaferin A |  | *Withania somnifera* | Withanolide | -7.66 | **-7.64** | [35] |
| 208 | Withanolide-D |  | *Withania somnifera* | Withanolide | -5.44 | **-7.13** | [35] |
| 209 | Withanolide-E |  | *Withania somnifera* | Withanolide | -5.18 | **-6.80** | [35] |
| 210 | Sitoindoside-X |  | *Withania somnifera* | Withanolide saponin | -**10.61** | **-9.69** | [35] |
| 211 | Withanolide A |  | *Withania somnifera* | Steroidal lactone | -6.79 | **-6.87** | [36] |
| 212 | Withanolide B |  | *Withania somnifera* | Steroidal lactone | -6.64 | -**7.07** | [36] |
| 213 | Withanolide C |  | *Withania somnifera* | Steroidal lactone | - | - | [36] |
| 214 | Withanolide F |  | *Withania somnifera* | Steroidal lactone | -5.82 | **-6.68** | [36] |
| 215 | Withanolide G |  | *Withania somnifera* | Steroidal lactone | -5.66 | **-6.40** | [36] |
| 216 | Withanolide H |  | *Withania somnifera* | Steroidal lactone | -6.32 | **-7.02** | [36] |
| 217 | Withanolide I |  | *Withania somnifera* | Steroidal lactone | -6.12 | **-6.75** | [36] |
| 218 | Withanolide J |  | *Withania somnifera* | Steroidal lactone | -6.27 | -**6.73** | [36] |
| 219 | Withanolide K |  | *Withania somnifera* | Steroidal lactone | -8.35 | **-7.40** | [36] |
| 220 | Withanolide L |  | *Withania somnifera* | Steroidal lactone | -6.37 | **-6.91** | [36] |
| 221 | Withanolide M |  | *Withania somnifera* | Steroidal lactone | -6.43 | -**6.96** | [36] |
| 222 | Withanolide N |  | *Withania somnifera* | Steroidal lactone | -5.82 | **-6.30** | [36] |
| 223 | Withanolide O |  | *Withania somnifera* | Steroidal lactone | - | - | [36] |
| 224 | Withanolide P |  | *Withania somnifera* | Steroidal lactone | -6.52 | **-6.81** | [36] |
| 225 | Withanolide Q |  | *Withania somnifera* | Steroidal lactone | -6.40 | **-6.67** | [36] |
| 226 | Withanolide R |  | *Withania somnifera* | Steroidal lactone | - | - | [36] |
| 227 | Withanolide S |  | *Withania somnifera* | Steroidal lactone | - | - | [36] |
| 228 | Withanolide T |  | *Withania somnifera* | Steroidal lactone | -6.18 | **-6.73** | [36] |
| 229 | Withanolide U |  | *Withania somnifera* | Steroidal lactone | -9.53 | **-7.71** | [36] |
| 230 | Withanolide Y |  | *Withania somnifera* | Steroidal lactone | - | - | [36] |
| 231 | Withanone |  | *Withania somnifera* | Withanolide | -6.61 | **-7.13** | [36] |
| 232 | Sominone |  | *Withania somnifera* | Aglycone of withanoside IV | -7.29 | **-6.71** | [36] |
| 233 | Dunawithagenin |  | *Withania somnifera* | Withanolide | - | - | [36] |
| 234 | D 16 Withanolide |  | *Withania somnifera* | Withanolide | - | - | [36] |
| 235 | Sominolide |  | *Withania somnifera* | Withanolide | -6.27 | **-7.35** | [36] |
| 236 | Withasomniferin A |  | *Withania somnifera* | Withanolide | -5.44 | **-7.01** | [36] |
| 237 | Withasomidienone |  | *Withania somnifera* | Withanolide | -8.09 | -**7.38** | [36] |
| 238 | Withaoxylactone |  | *Withania somnifera* | Withanolide | -7.25 | **-7.87** | [36] |
| 239 | Somnifericin |  | *Withania somnifera* | Withanolide | -6.89 | **-7.31** | [36] |
| 240 | Withasomnilide |  | *Withania somnifera* | Withanolide | -6.95 | **-6.70** | [36] |
| 241 | Withasomniferanolide |  | *Withania somnifera* | Withanolide | -5.58 | **-6.35** | [36] |
| 242 | Somniferawithanolide |  | *Withania somnifera* | Withanolide | -8.91 | **-6.30** | [36] |
| 243 | Somniferanolide |  | *Withania somnifera* | Withanolide | -9.72 | **-6.50** | [36] |
| 244 | Somniwithanolide |  | *Withania somnifera* | Withanolide | -7.16 | **-7.41** | [36] |
| 245 | Withasomniferol A |  | *Withania somnifera* | Withanolide | -6.95 | **-6.92** | [36] |
| 246 | Withasomniferol B |  | *Withania somnifera* | Withanolide | -6.22 | **-6.64** | [36] |
| 247 | Withasomniferol C |  | *Withania somnifera* | Withanolide | -5.49 | - | [36] |
| 248 | Viscosalactone B |  | *Withania somnifera* | Withanolide | -8.21 | **-6.90** | [36] |
| 249 | Gallic acid |  | *Terminalia chebula* | Tannins | -5.06 | -4.56 | [34] |
| 250 | Chebulagic acid |  | *Terminalia chebula* | Tannin | -6.67 | **-8.86** | [34] |
| 251 | Punicalagin |  | *Terminalia chebula* | Tannin | -5.91 | **-11.68** | [34] |
| 252 | Chebulanin |  | *Terminalia chebula* | Tannin | -6.28 | **-8.02** | [34] |
| 253 | Corilagin |  | *Terminalia chebula* | Tannin | -7.23 | -**7.67** | [34] |
| 254 | Neo-chebulinic acid |  | *Terminalia chebula* | Tannin | -8.19 | **-10.86** | [34] |
| 255 | Ellagic acid |  | *Terminalia chebula* | Tannin | -6.33 | **-8.02** | [34] |
| 256 | Chebulinic acid |  | *Terminalia chebula* | Tannin | -7.56 | **-10.08** | [34] |
| 257 | 1,2,3,4,6-penta-O-galloyl-β-D-glucose |  | *Terminalia chebula* | Tannin | -7.14 | **-11.01** | [34] |
| 258 | 1,6-di-o-galloyl-D-glucose |  | *Terminalia chebula* | Tannin | -10.19 | **-7.00** | [34] |
| 259 | Casuarinin |  | *Terminalia chebula* | Tannin | -4.98 | **-9.07** | [34] |
| 260 | 3,4,6-tri-o-glloyl-D-glucose |  | *Terminalia chebula* | Tannin | -9.43 | **-7.71** | [34] |
| 261 | Terchebulin |  | *Terminalia chebula* | Tannin | -6.33 | **-9.94** | [34] |
| 262 | Terflavin A |  | *Terminalia chebula* | Tannin | -8.86 | **-10.15** | [34] |
| 263 | 1, 3, 6-trigalloyl glucose |  | *Terminalia chebula* | Tannin | -8.02 | **-9.01** | [34] |
| 264 | Terflavins B |  | *Terminalia chebula* | Tannin | -7.62 | **-8.87** | [34] |
| 265 | Terflavin C |  | *Terminalia chebula* | Tannin | -6.53 | **-9.79** | [34] |
| 266 | Terflavin D |  | *Terminalia chebula* | Tannin | -7.24 | **-7.84** | [34] |
| 267 | Punicalin |  | *Terminalia chebula* | Tannin | -6.04 | **-7.95** | [34] |
| 268 | Chebulic acid |  | *Terminalia chebula* | Tannin | -5.30 | -5.15 | [34] |
| 269 | Neo-chebulic acid |  | *Terminalia chebula* | Tannin | -5.50 | -5.86 | [34] |
| 270 | 4-O-methylgallic acid |  | *Terminalia chebula* | Tannin | -5.46 | -4.14 | [34] |
| 271 | Ethyl gallate |  | *Terminalia chebula* | Tannin | -5.44 | -4.59 | [34] |
| 272 | Methyl gallate |  | *Terminalia chebula* | Tannin | -5.16 | -4.77 | [34] |
| 273 | Methyl(S)-flavogallonate |  | *Terminalia chebula* | Tannin | -6.52 | -5.95 | [34] |
| 274 | Methyl neochebulagate |  | *Terminalia chebula* | Tannin | - | - | [34] |
| 275 | Eugenol |  | *Terminalia chebula* | Tannin | -5.46 | -4.45 | [34] |
| **276** | **Tannic acid** |  | ***Terminalia chebula*** | **Tannin** | **-11.08** | **-13.59** | [34] |
| 277 | Triethyl chebulate |  | *Terminalia chebula* | Tannin | -7.53 | -5.95 | [34] |
| 278 | 2, 4-Chebulyl-beta-D-glucopyranose |  | *Terminalia chebula* | Tannin | -5.75 | **-6.29** | [34] |
| 279 | Arjungenin |  | *Terminalia chebula* | Triterpenoid | -3.06 | **-6.23** | [34] |
| 280 | Arjunolic acid |  | *Terminalia chebula* | Triterpenoid saponin | -4.26 | -6.01 | [34] |
| 281 | Arjunic acid |  | *Terminalia chebula* | Triterpenoid | -4.22 | **-6.46** | [34] |
| 282 | Terminolic acid |  | *Terminalia chebula* | Triterpenoid | -4.06 | **-6.55** | [34] |
| 283 | Arjunglucoside I |  | *Terminalia chebula* | Triterpenoid | -4.39 | **-7.74** | [34] |
| 284 | Arjunglucoside II |  | *Terminalia chebula* | Triterpenoid | -6.17 | **-7.27** | [34] |
| 285 | Arjunetin |  | *Terminalia chebula* | Triterpenoid | -5.70 | **-7.54** | [34] |
| 286 | Chebuloside II |  | *Terminalia chebula* | Triterpenoid glycosides | -5.71 | -**7.55** | [34] |
| 287 | Bellericoside |  | *Terminalia chebula* | Triterpenoid saponin | -5.80 | -**7.74** | [42] |
| 288 | Chebuloside I |  | *Terminalia chebula* | Triterpenoid glycosides | -4.81 | **-8.06** | [42] |
| 289 | 2α-Hydroxyursolic acid |  | *Terminalia chebula* | Triterpenoid | - | - | [42] |
| 290 | Maslinic acid |  | *Terminalia chebula* | Triterpenoid | -3.06 | -5.80 | [42] |
| 291 | β-Caryophyllene |  | *Terminalia chebula* | Sesquiterpenoids | -5.38 | -4.92 | [42] |
| 292 | α-Phellandrene |  | *Terminalia chebula* | Monoterpenes | -5.03 | -4.48 | [42] |
| 293 | α-Terpinene |  | *Terminalia chebula* | Monoterpene | -5.05 | -4.66 | [42] |
| 294 | Terpinen-4-ol |  | *Terminalia chebula* | Monoterpenes | -5.57 | -4.91 | [42] |
| 295 | Terpinolene |  | *Terminalia chebula* | Monoterpene | -5.40 | -4.85 | [42] |
| 296 | Chebupentol |  | *Terminalia chebula* | Triterpenoid | -5.09 | **-6.58** | [42] |
| 297 | Rutin |  | *Terminalia chebula* | Flavanoid | -7.25 | -**8.33** | [42] |
| 298 | Quercetin |  | *Terminalia chebula* | Favonoid | -6.72 | -4.81 | [42] |
| 299 | Luteolin |  | *Terminalia chebula* | Flavanoid | -7.11 | -5.15 | [42] |
| 300 | Isoquercetin |  | *Terminalia chebula* | Flavanoid | -7.04 | **-6.92** | [42] |
| 301 | 3’-Methoxy quercetin |  | *Terminalia chebula* | Flavanoid | -7.25 | -5.35 | [42] |
| 302 | 3, 4-Dimethoxy quercetin |  | *Terminalia chebula* | Flavanoid | -7.57 | -5.78 | [42] |
| 303 | Pelargonidin |  | *Terminalia chebula* | Flavanoid | -7.42 | -5.52 | [42] |
| 304 | Phloroglucinol [benzene-1, 3, 5-triol ] |  | *Terminalia chebula* | Phenol | -4.30 | -4.02 | [42] |
| 305 | Pyragallol [1, 2, 3-Trihydroxybenzene] |  | *Terminalia chebula* | Phenol | -5.00 | -4.26 | [42] |
| 306 | Phenol |  | *Terminalia chebula* | Phenol | -4.73 | -3.84 | [42] |
| 307 | Shikimic acid |  | *Terminalia chebula* | Phenolic Carboxylic Compounds | -5.52 | -4.28 | [42] |
| 308 | Ferulic acid |  | *Terminalia chebula* | Phenolic Carboxylic Compounds | -5.64 | -4.23 | [42] |
| 309 | Vanillic acid |  | *Terminalia chebula* | Phenolic Carboxylic Compounds | -5.41 | -4.66 | [42] |
| 310 | P-Coumaric acid |  | *Terminalia chebula* | Phenolic Carboxylic Compounds | -5.54 | -4.23 | [42] |
| 311 | 3-(3,4-Dihydroxyphenyl)-2-propenoic acid |  | *Terminalia chebula* | Phenolic Carboxylic Compounds | -5.65 | -4.07 | [42] |
| 312 | Melilotic acid |  | *Terminalia chebula* | Phenolic Carboxylic Compounds | -5.06 | -5.06 | [42] |
| 313 | Asiatic acid |  | *Centella asiatica* | Triterpenes | -5.17 | **-6.44** | [43] |
| 314 | Madecassic acid/Brahmic acid |  | *Centella asiatica* | Triterpenes | -3.92 | **-6.53** | [43] |
| 315 | 23-  trihydroxyurs-20-en-28-oic acid, |  | *Centella asiatica* | Triterpenes | -3.86 | **-7.11** | [43] |
| 316 | 2α, 3β, 20, 23-tetrahydroxyurs-28-oic acid |  | *Centella asiatica* | Triterpenes | - | - | [43] |
| 317 | Pomolic acid |  | *Centella asiatica* | Triterpenes | -3.76 | **-6.29** | [43] |
| 318 | Corosolic acid |  | *Centella asiatica* | Triterpenes | -2.47 | **-6.55** | [43] |
| 319 | Ursolic acid |  | *Centella asiatica* | Triterpenes | -2.30 | -6.18 | [43] |
| 320 | Asiaticoside |  | *Centella asiatica* | Triterpenes | -**10.49** | **-8.66** | [43] |
| 321 | Madecassoside/ Asiaticoside a |  | *Centella asiatica* | Triterpenes | - | - | [43] |
| 322 | Isothankuniside |  | *Centella asiatica* | Triterpenes | - | - | [43] |
| 323 | Centellasaponin B |  | *Centella asiatica* | Triterpenes | -7.91 | **-8.81** | [43] |
| 324 | Centellasaponin C |  | *Centella asiatica* | Triterpenes | -9.10 | **-9.39** | [43] |
| **325** | **Asiaticoside C** |  | ***Centella asiatica*** | **Triterpenes** | **-10.33** | **-9.93** | [43] |
| 326 | Asiaticoside D |  | *Centella asiatica* | Triterpenes | -8.95 | **-9.57** | [43] |
| 327 | Asiaticoside E |  | *Centella asiatica* | Triterpenes | **-6.17** | **-8.03** | [43] |
| 328 | Asiaticoside F |  | *Centella asiatica* | Triterpenes | - | - | [43] |
| 329 | Quadranoside IV |  | *Centella asiatica* | Triterpenes | **-6.60** | **-7.56** | [43] |
| 330 | Isoasiaticoside |  | *Centella asiatica* | Triterpenes | **-9.28** | **-9.24** | [43] |
| 331 | Isoasiatic acid |  | *Centella asiatica* | Triterpenes | - | - | [43] |
| 332 | Isomadecassoside |  | *Centella asiatica* | Triterpenes | - | - | [43] |
| 333 | 23-O-Acetyl  Madecassoside |  | *Centella asiatica* | Triterpenes | -9.31 | **-9.82** | [43] |
| 334 | Asiaticoside G |  | *Centella asiatica* | Triterpenes | **-10.54** | **-9.10** | [43] |
| 335 | Asiaticoside H |  | *Centella asiatica* | Triterpenes | -7.50 | **-9.72** | [43] |
| 336 | Asiaticoside I |  | *Centella asiatica* | Triterpenes | **-10.54** | **-9.73** | [43] |
| 337 | Centelloside C |  | *Centella asiatica* | Triterpenes | -5.08 | **-8.38** | [43] |
| 338 | Centelloside E |  | *Centella asiatica* | Triterpenes | -9.26 | **-9.81** | [43] |
| 339 | Centelloside G |  | *Centella asiatica* | Triterpenes | - | -**9.65** | [43] |
| 340 | Centellasaponin J |  | *Centella asiatica* | Triterpenes | -8.05 | **-9.89** | [43] |
| 341 | Centellasaponin F |  | *Centella asiatica* | Triterpenes | - | - | [43] |
| 342 | Centellasaponin G |  | *Centella asiatica* | Triterpenes | -10.24 | **-10.81** | [43] |
| 343 | 11-Oxo-madecassoside |  | *Centella asiatica* | Triterpenes | - | - | [43] |
| 344 | 11(B)-Methoxy madecassoside |  | *Centella asiatica* | Triterpenes | - | - | [43] |
| 345 | Asiaticoside B |  | *Centella asiatica* | Triterpenes | -9.82 | **-9.59** | [43] |
| 346 | Centellasaponin A |  | *Centella asiatica* | Triterpenes | **-12.07** | **-10.31** | [43] |
| 347 | Centellasaponin D |  | *Centella asiatica* | Triterpenes | -9.82 | **-9.23** | [43] |
| 348 | 23-O-acetyl  asiaticoside B |  | *Centella asiatica* | Triterpenes | **-11.12** | **-9.99** | [43] |
| 349 | Chebuloside II |  | *Centella asiatica* | Triterpenes | -5.72 | **-7.73** | [43] |
| 350 | Centelloside D |  | *Centella asiatica* | Triterpenes | -5.72 | **-9.83** | [43] |
| 351 | centelloside F |  | *Centella asiatica* | Triterpenes | **-10.49** | **-9.15** | [43] |
| 352 | Centellasaponin I |  | *Centella asiatica* | Triterpenes | **-10.48** | **-9.43** | [43] |
| 353 | Centellasaponin H |  | *Centella asiatica* | Triterpenes | -10.50 | **-10.17** | [43] |
| 354 | Centellasaponin E |  | *Centella asiatica* | Triterpenes | - | - | [43] |
| 355 | 11-Oxo-asiaticoside B |  | *Centella asiatica* | Triterpenes | - | - | [43] |
| 356 | 11(B)-Methoxy asiaticoside B |  | *Centella asiatica* | Triterpenes | - | - | [43] |
| 357 | Centelloside A |  | *Centella asiatica* | Triterpenes | **-11.77** | **-9.68** | [43] |
| 358 | Centelloside B |  | *Centella asiatica* | Triterpenes | - | - | [43] |
| 359 | Madasiatic acid |  | *Centella asiatica* | Triterpenes | -2.27 | -6.41 | [43] |
| 360 | Isothankunic acid |  | *Centella asiatica* | Triterpenes | - | - | [43] |
| 361 | Scheffursoside F |  | *Centella asiatica* | Triterpenes | -9.56 | -9.57 | [43] |
| 362 | Scheffuroside B |  | *Centella asiatica* | Triterpenes | - | - | [43] |
| 363 | Scheffoleoside A |  | *Centella asiatica* | Triterpenes | **-10.73** | **-9.71** | [43] |
| 364 | Castasterone |  | *Centella asiatica* | Steroid | -6.83 | -**7.00** | [43] |
| 365 | Castellicetin |  | *Centella asiatica* | Flavanoid | -7.79 | **-7.11** | [43] |
| 366 | Castilliferol |  | *Centella asiatica* | Flavanoid | -7.34 | **-6.51** | [43] |
| 367 | Kaempferol |  | *Centella asiatica* | Flavanoid | -7.27 | -5.07 | [43] |
| 368 | kaempferol-3-o-β-d-glucuronide |  | *Centella asiatica* | Flavanoid | -9.09 | -**6.86** | [43] |
| 369 | Quercetin |  | *Centella asiatica* | Flavanoid | -7.37 | -5.37 | [43] |
| 370 | quercitin-3-o-β -d-glucuronide |  | *Centella asiatica* | Flavanoid | -7.89 | **-6.78** | [43] |
| 371 | Rosmarinic acid |  | *Centella asiatica* | Phenylpropanoids | -8.00 | -6.06 | [43] |
| 372 | chlorogenic acid |  | *Centella asiatica* | Phenylpropanoids | -8.14 | -**6.24** | [43] |
| 373 | 3,4-di-o-caffeoyl quinic acid |  | *Centella asiatica* | Phenylpropanoids | -9.96 | **-7.40** | [43] |
| 374 | 1,5-di-o-caffeoyl quinic acid |  | *Centella asiatica* | Phenylpropanoids | -**10.37** | **-7.50** | [43] |
| 375 | 3,5-di-o-caffeoyl quinic acid |  | *Centella asiatica* | Phenylpropanoids | -8.65 | **-7.81** | [43] |
| 376 | 4,5-di-o-caffeoyl quinic acid |  | *Centella asiatica* | Phenylpropanoids | -7.43 | **-7.61** | [43] |
| 377 | isochlorogenic acid |  | *Centella asiatica* | Phenylpropanoids | -8.23 | -5.72 | [44] |
| 378 | Irbic acid |  | *Centella asiatica* | Phenolic acid | -8.67 | **-8.91** | [44] |
| 379 | Emblicanin A |  | *Emblica officinalis .* | Tannin | -9.67 | **-9.00** | [37] |
| 380 | Emblicanin B |  | *Emblica officinalis* | Tannin | -6.55 | **-8.36** | [37] |
| 381 | Punigluconin |  | *Emblica officinalis* | Tannin | -8.32 | -**9.50** | [37] |
| 382 | Pedunculagin |  | *Emblica officinalis* | Tannin | -5.70 | **-8.54** | [37] |
| 383 | Chebulinic acid |  | *Emblica officinalis* | Tannin | -6.94 | **-9.26** | [37] |
| 384 | Chebulagic acid |  | *Emblica officinalis* | Tannin | -6.88 | **-9.76** | [37] |
| 385 | Corilagin |  | *Emblica officinalis* | Tannin | -6.55 | **-8.04** | [37] |
| 386 | Geraniin |  | *Emblica officinalis* | Tannin | - | - | [37] |
| 387 | Phyllantidine |  | *Emblica officinalis* | Alkaloids | -6.45 | -4.95 | [37] |
| 388 | Phyllanthin |  | *Emblica officinalis* | Alkaloids | -7.05 | **-7.30** | [37] |
| 389 | Glucogallin |  | *Emblica officinalis* | Phenolic compound | -7.52 | -6.17 | [37] |
| 390 | Gallic acid |  | *Emblica officinalis* | Phenolic compound | -5.02 | -4.24 | [37] |
| 391 | 3,6-di-O-galloyl-d-glucose |  | *Emblica officinalis* | Phenolic compound | -9.98 | **-7.57** | [37] |
| 392 | 1,6-di-O-galloyl-B-d-glucose |  | *Emblica officinalis* | Phenolic compound | -9.78 | -7.48 | [37] |
| 393 | Methyl gallate |  | *Emblica officinalis* | Phenolic compound | -5.16 | -4.74 | [37] |
| 394 | Ellagic acid |  | *Emblica officinalis* | Phenolic compound | -6.33 | -5.21 | [37] |
| 395 | Trigallayl glucose |  | *Emblica officinalis* | Phenolic compound | -8.33 | **-8.21** | [37] |
| 396 | 3-ethylgallic  acid |  | *Emblica officinalis* | Phenolic compound | -6.13 | -4.79 | [37] |
| 397 | l-malic acid 2-O-gallate |  | *Emblica officinalis* | Phenolic compound | -6.79 | -5.06 | [37] |
| 398 | Mucic acid 2-  O-gallate |  | *Emblica officinalis* | Phenolic compound | -8.38 | -5.78 | [37] |
| 399 | Mucic acid 1,4-lactone 5-O-gallate |  | *Emblica officinalis* | Phenolic compound | -8.14 | -5.75 | [37] |
| 400 | Phyllaemblicin-A |  | *Emblica officinalis* | Norsesquiterpenoid | -7.58 | **-7.60** | [37] |
| 401 | Phyllaemblicin B |  | *Emblica officinalis* | Norsesquiterpenoid | -9.36 | **-8.19** | [37] |
| 402 | Phyllaemblicin C |  | *Emblica officinalis* | Nor-sesquiterpenoid | -7.32 | **-7.96** | [37] |
| 403 | Phyllaemblic acid B |  | *Emblica officinalis* | Sesquiterpenoids | -5.93 | -5.33 | [37] |
| 404 | Phyllaemblic acid C |  | *Emblica officinalis* | Sesquiterpenoids | -6.25 | -5.15 | [37] |
| 405 | Phyllaemblicin D |  | *Emblica officinalis* | Sesquiterpenoids | -9.40 | **-6.86** | [37] |
| 406 | 2-carboxylmethylphenol 1-O-B-d-glucopyranoside |  | *Emblica officinalis* | Phenolic glycosides | -7.77 | -6.10 | [37] |
| 407 | 2-6-dimethoxy-4-(2-hydroxyethyl)phenol 1-O-B-d-glucopyranoside |  | *Emblica officinalis* | Phenolic glycosides | -6.51 | -5.76 | [37] |
| 408 | Phyllanemblinin A |  | *Emblica officinalis* | Ellagitanin | -7.77 | **-7.15** | [37] |
| 409 | Phyllanemblinin B |  | *Emblica officinalis* | Ellagitanin | -6.12 | **-8.13** | [37] |
| 410 | Phyllanemblinin C |  | *Emblica officinalis* | Ellagitanin | - | - | [37] |
| 411 | Phyllanemblinin D |  | *Emblica officinalis* | Ellagitanin | -9.29 | **-8.55** | [37] |
| 412 | Phyllanemblinin E |  | *Emblica officinalis* | Ellagitanin | -8.85 | **-8.37** | [37] |
| 413 | Phyllanemblinin F |  | *Emblica officinalis* | Ellagitanin | -10.24 | **-8.26** | [37] |
| 414 | Apigenin-7-O-(6'-butyryl-B-glucopyranoside). |  | *Emblica officinalis* | Apigenin | -9.05 | **-7.54** | [37] |
| 415 | (S)-eriodictyol 7-O-(6-B--O-trans-p-coumaroyl)-B-d-glucopyranoside |  | *Emblica officinalis* | Acrylated flavanone glycosides | **-10.72** | **-8.68** | [37] |
| 416 | (2S)-eriodictyol 7-O-(6-B -O-galloyl)-B-d-glucopyranoside |  | *Emblica officinalis* | Acrylated flavanone glycosides | -9.82 | **-8.54** | [37] |
| 417 | 1,2,3,4,6-penta-O-galloylglucose |  | *Emblica officinalis* | Gallotannin | -7.61 | **-10.88** | [37] |
| 418 | Luteolin-4-O-neohesperidoside |  | *Emblica officinalis* | Flavanoid | -8.26 | **-7.45** | [37] |
| 419 | Trihydroxysitosterol |  | *Emblica officinalis* | Sterol | -6.24 | **-7.48** | [37] |
| 420 | 5-hydroxymethylfurfural |  | *Emblica officinalis* | Furan | -4.60 | -4.27 | [37] |
| 421 | 2-acetyl-5-methyl furan |  | *Emblica officinalis* | Furan | -4.79 | -3.94 | [37] |
| 422 | Pyragallol [1, 2, 3-Trihydroxybenzene] |  | *Emblica officinalis* | Phenol | -5.09 | -4.26 | [37] |
| 423 | Propyl 3,4,5-trihydroxybenzoate |  | *Emblica officinalis* | Polyphenol | -6.54 | -4.69 | [37] |
| 424 | 3,5,7,3’,4’-penta-hydroxy flavone |  | *Emblica officinalis* | Flavanoid | -8.38 | **-6.33** | [37] |
| 425 | 5-(1,2-dihydroxyethyl)-3,4-dihydroxy-2,5-dihydrofuran-2-one |  | *Emblica officinalis* | Polyphenol | -5.49 | -4.25 | [37] |
| 426 | P-Coumaric acid |  | *Emblica officinalis* | Hydroxycinnamic acids | -5.55 | -4.04 | [37] |
| 427 | Myricetin |  | *Emblica officinalis* | Flavone | -7.38 | -4.81 | [37] |
| 428 | Caffeic acid |  | *Emblica officinalis* | Polyphenol | -5.64 | -4.04 | [37] |
| 429 | Syringic acid |  | *Emblica officinalis* | Phenol | -5.09 | -4.68 | [37] |
| 430 | Quercetin |  | *Emblica officinalis* | Flavanoid | -7.64 | -5.19 | [37] |
| 431 | Kaempferol |  | *Emblica officinalis* | Flavanoid | -7.25 | -5.04 | [37] |
| 432 | Arbutin |  | *Nelumbo nucifera* | Aromatic phenolic compounds | -7.36 | -5.00 | [41] |
| 433 | Gallic acid |  | *Nelumbo nucifera* | Aromatic phenolic compounds | -5.02 | -4.04 | [41] |
| 434 | Ferulic acid |  | *Nelumbo nucifera* | Aromatic phenolic compounds | -5.63 | -4.27 | [41] |
| 435 | p-Coumaric acid |  | *Nelumbo nucifera* | Aromatic phenolic compounds | -5.55 | -4.04 | [41] |
| 436 | p-Hydroxybenzoic acid |  | *Nelumbo nucifera* | Aromatic phenolic compounds | -4.48 | -3.59 | [41] |
| 437 | Protocatechuic acid |  | *Nelumbo nucifera* | Aromatic phenolic compounds | -4.86 | -4.03 | [41] |
| 438 | Tannic acid |  | *Nelumbo nucifera* | Aromatic phenolic compounds | **-13.19** | **-13.31** | [41] |
| 439 | (−)-Boscialin |  | *Nelumbo nucifera* | Megastigmane/sesquiterpenes compounds | -5.00 | -4.69 | [41] |
| 440 | (+)-Dehydrovomifoliol |  | *Nelumbo nucifera* | Megastigmane/sesquiterpenes compounds | -5.52 | -5.15 | [41] |
| 441 | (+)-Epiloliolide |  | *Nelumbo nucifera* | Megastigmane/sesquiterpenes compounds | -4.99 | -4.57 | [41] |
| 442 | 3-oxo-Retro-α-ionol I |  | *Nelumbo nucifera* | Megastigmane/sesquiterpenes compounds | -6.13 | -5.49 | [41] |
| 443 | 5,6-epoxy-3-Hydroxy-7-megastigmen-9-one |  | *Nelumbo nucifera* | Megastigmane/sesquiterpenes compounds | -5.57 | -5.98 | [41] |
| 444 | Annuionone D |  | *Nelumbo nucifera* | Megastigmane/sesquiterpenes compounds | -5.68 | -5.59 | [41] |
| 445 | Byzantionoside A |  | *Nelumbo nucifera* | Megastigmane/sesquiterpenes compounds | -7.48 | **-6.28** | [41] |
| 446 | Grasshopper ketone |  | *Nelumbo nucifera* | Megastigmane/sesquiterpenes compounds | -6.17 | -5.28 | [41] |
| 447 | Icariside B2 |  | *Nelumbo nucifera* | Megastigmane/sesquiterpenes compounds | -8.10 | -5.41 | [41] |
| 448 | Nelumnucifoside A |  | *Nelumbo nucifera* | Megastigmane/sesquiterpenes compounds | -7.11 | **-6.70** | [41] |
| 449 | Nelumnucifoside B |  | *Nelumbo nucifera* | Megastigmane/sesquiterpenes compounds | - | - | [41] |
| 450 | Vomifoliol |  | *Nelumbo nucifera* | Megastigmane/sesquiterpenes compounds | -5.56 | -4.97 | [41] |
| 451 | (−)-1(R)-N-methylcoclaurine |  | *Nelumbo nucifera* | Alkaloid | -7.20 | -5.83 | [41] |
| 452 | (−)-Anonaine |  | *Nelumbo nucifera* | Alkaloid | -5.09 | -5.09 | [41] |
| 453 | (−)-Asimilobine |  | *Nelumbo nucifera* | Alkaloid | -6.29 | -4.86 | [41] |
| 454 | (−)-Caaverine |  | *Nelumbo nucifera* | Alkaloid | -5.67 | -4.98 | [41] |
| 455 | (−)-N-Methylasimilobine |  | *Nelumbo nucifera* | Alkaloid | -5.62 | -5.39 | [41] |
| 456 | (−)-nor-Nuciferine |  | *Nelumbo nucifera* | Alkaloid | -5.63 | -5.38 | [41] |
| 457 | (−)-Nuciferine |  | *Nelumbo nucifera* | Alkaloid | -5.45 | -5.72 | [41] |
| 458 | (−)-Roemerine |  | *Nelumbo nucifera* | Alkaloid | -4.78 | -5.51 | [41] |
| 459 | 2-Hydroxy-1-methoxy-6a,7-dehydroaporphine |  | *Nelumbo nucifera* | Alkaloid | -4.79 | -5.45 | [41] |
| 460 | 3-Indoleacetic acid |  | *Nelumbo nucifera* | Alkaloid | -5.99 | -4.90 | [41] |
| 461 | Anisic acid |  | *Nelumbo nucifera* | Alkaloid | -5.03 | -4.33 | [41] |
| 462 | Armepavine |  | *Nelumbo nucifera* | Alkaloid | -6.63 | -5.99 | [41] |
| 463 | Cepharadione B |  | *Nelumbo nucifera* | Alkaloid | -4.95 | -5.75 | [41] |
| 464 | cis-N-Coumaroyltyramine |  | *Nelumbo nucifera* | Alkaloid | -6.85 | -5.34 | [41] |
| 465 | cis-N-Feruloyltyramine |  | *Nelumbo nucifera* | Alkaloid | -8.22 | -6.15 | [41] |
| 466 | Coclaurine |  | *Nelumbo nucifera* | Alkaloid | -6.95 | -5.74 | [41] |
| 467 | Dauricine |  | *Nelumbo nucifera* | Alkaloid | -8.78 | **-8.64** | [41] |
| 468 | Dehydroanonaine |  | *Nelumbo nucifera* | Alkaloid | -5.11 | -5.18 | [41] |
| 469 | Dehydroemetine |  | *Nelumbo nucifera* | Alkaloid | -8.36 | **-7.58** | [41] |
| 470 | Dehydronuciferine |  | *Nelumbo nucifera* | Alkaloid | -4.97 | -5.40 | [41] |
| 471 | Demethylcoclaurine |  | *Nelumbo nucifera* | Alkaloid | -6.67 | -4.79 | [41] |
| 472 | Isoliensinine |  | *Nelumbo nucifera* | Alkaloid | -9.48 | **-7.98** | [41] |
| 473 | Liensinine |  | *Nelumbo nucifera* | Alkaloid | **-10.31** | **-8.07** | [41] |
| 474 | Liriodenine |  | *Nelumbo nucifera* | Alkaloid | -5.82 | -5.13 | [41] |
| 475 | Lotusine |  | *Nelumbo nucifera* | Alkaloid | -5.58 | -6.09 | [41] |
| 476 | Lysicamine |  | *Nelumbo nucifera* | Alkaloid | -6.38 | -5.34 | [41] |
| 477 | Neferine |  | *Nelumbo nucifera* | Alkaloid | -8.42 | **-8.61** | [41] |
| 478 | N-Norarmepavine |  | *Nelumbo nucifera* | Alkaloid | -7.65 | **-6.29** | [41] |
| 479 | N-Nornuciferine |  | *Nelumbo nucifera* | Alkaloid | -6.14 | -5.59 | [41] |
| 480 | Norjuziphine |  | *Nelumbo nucifera* | Alkaloid | -7.37 | **-6.24** | [41] |
| 481 | Nuciferine N-oxide |  | *Nelumbo nucifera* | Alkaloid | -5.56 | -5.42 | [41] |
| 482 | Oleracein E |  | *Nelumbo nucifera* | Alkaloid | -6.38 | -4.52 | [41] |
| 483 | Pronuciferine |  | *Nelumbo nucifera* | Alkaloid | -5.01 | -5.98 | [41] |
| 484 | Reserpine |  | *Nelumbo nucifera* | Alkaloid | -8.84 | **-8.09** | [41] |
| 485 | Roemerin |  | *Nelumbo nucifera* | Alkaloid | -4.70 | -5.31 | [41] |
| 486 | trans-N-Coumaroyltyramine |  | *Nelumbo nucifera* | Alkaloid | -7.88 | -6.10 | [41] |
| 487 | trans-N-Feruloyltyramine |  | *Nelumbo nucifera* | alkaloids | -8.51 | **-6.27** | [41] |
| 488 | (−)-Catechin |  | *Nelumbo nucifera* | Flavanoid | -6.95 | -5.59 | [41] |
| 489 | 5,7,3’,5’-Tetrahydroxyflavanone |  | *Nelumbo nucifera* | Flavanoid | -7.57 | -5.46 | [41] |
| 490 | Chrysoeriol 7-O-β-D-glucopyranoside |  | *Nelumbo nucifera* | Flavanoid | -9.17 | **-6.46** | [41] |
| 491 | Elephantorrhizol |  | *Nelumbo nucifera* | Flavanoid | -7.32 | -5.83 | [41] |
| 492 | Epitaxifolin |  | *Nelumbo nucifera* | Flavanoid | -7.38 | -5.53 | [41] |
| 493 | Hyperoside |  | *Nelumbo nucifera* | Flavanoid | -7.03 | **-6.77** | [41] |
| 494 | Isoquercitrin (Hirsutrin) |  | *Nelumbo nucifera* | Flavanoid | -6.61 | **-7.03** | [41] |
| 495 | Isorhamnetin |  | *Nelumbo nucifera* | Flavanoid | -7.82 | -5.64 | [41] |
| 496 | Isorhamnetin 3-O-β-D-glucopyranoside |  | *Nelumbo nucifera* | Flavanoid | -7.14 | **-6.88** | [41] |
| 497 | Isorhamnetin 3-O-rutinoside |  | *Nelumbo nucifera* | Flavanoid | -8.08 | **-8.94** | [41] |
| 498 | Isoschaftoside |  | *Nelumbo nucifera* | Flavanoid | -8.08 | **-7.25** | [41] |
| 499 | Kaempferol |  | *Nelumbo nucifera* | Flavanoid | -7.25 | -5.04 | [41] |
| 500 | Kaempferol 3-O-robinobioside |  | *Nelumbo nucifera* | Flavanoid | -8.98 | **-7.92** | [41] |
| 501 | Astragalin |  | *Nelumbo nucifera* | Flavanoid | -6.89 | **-6.56** | [41] |
| 502 | Kaempferol 3-O-β-D-galactopyranoside/Trifolin |  | *Nelumbo nucifera* | Flavanoid | -6.73 | **-6.67** | [41] |
| 503 | Kaempferol 7-O-β-D-glucopyranoside |  | *Nelumbo nucifera* | Flavanoid | -8.70 | **-7.01** | [41] |
| 504 | Luteolin |  | *Nelumbo nucifera* | Flavanoid | -7.12 | -5.09 | [41] |
| 505 | Myricetin 3-O-galactoside |  | *Nelumbo nucifera* | Flavanoid | -7.71 | **-6.90** | [41] |
| 506 | Myricetin 3-O-glucoside |  | *Nelumbo nucifera* | Flavanoid | -7.51 | **-6.78** | [41] |
| 507 | Myricetin 3-O-glucuronide |  | *Nelumbo nucifera* | Flavanoid | -7.78 | **-6.84** | [41] |
| 508 | Kaempferol 3-O-β-D-glucuronopyranoside |  | *Nelumbo nucifera* | Flavanoid | -6.63 | **-6.68** | [41] |
| 509 | Quercetin |  | *Nelumbo nucifera* | Flavanoid | -6.71 | -5.07 | [41] |
| 510 | quercitin-3-o-β -d-glucuronide |  | *Nelumbo nucifera* | Flavanoid | -7.35 | **-6.39** | [41] |
| 511 | Quercetin 3-O-β-D-glucopyranoside |  | *Nelumbo nucifera* | Flavanoid | -7.67 | -6.20 | [41] |
| 512 | Rutin |  | *Nelumbo nucifera* | Flavanoid | -7.00 | **-7.88** | [41] |
| 513 | Syringetin 3-O-glucoside |  | *Nelumbo nucifera* | Flavanoid | -7.37 | **-7.58** | [41] |
| 514 | Taxifolin |  | *Nelumbo nucifera* | Flavanoid | -6.81 | -4.92 | [41] |
| 515 | Kaempferol 3-O-α L-rhamnopyranosyl-(1→6)-β-D-glucopyranosid |  | *Nelumbo nucifera* | Flavanoid | - | - | [41] |
| 516 | Kaempferol 3-O-α-L-rhamnopyranosyl-(1→2)-β-Dglucopyranos |  | *Nelumbo nucifera* | Flavanoid | -8.44 | **-8.01** | [41] |
| 517 | Stigmasta-4,22-dien-3-one |  | *Nelumbo nucifera* | Sterols and triterpenoids | **-6.24** | **-6.89** | [41] |
| 518 | B-Sitostenone |  | *Nelumbo nucifera* | Sterols and triterpenoids | -7.61 | **-7.36** | [41] |
| 519 | β-Sitosterol |  | *Nelumbo nucifera* | Sterols and triterpenoids | -6.13 | **-7.25** | [41] |
| 520 | β-Sitosterol-3-O-glucoside |  | *Nelumbo nucifera* | Sterols and triterpenoids | - | - | [41] |
| 521 | Betulinic acid |  | *Nelumbo nucifera* | Sterols and triterpenoids | -3.58 | **-6.56** | [41] |
| 522 | α-Amyrin |  | *Nelumbo nucifera* | Sterols and triterpenoids | - | - | [41] |
| 523 | Tryptamine |  | *Punica granatum* | Alkaloids | -6.28 | -4.92 | [38] |
| 524 | Serotonin |  | *Punica granatum* | Alkaloids | -5.47 | -4.02 |  |
| 525 | Melatonin |  | *Punica granatum* | Alkaloids | -7.01 | -6.02 | [38] |
| 526 | Pelletierine |  | *Punica granatum* | Alkaloids | -5.12 | -3.92 | [38] |
| 527 | N-Methylpelletierine |  | *Punica granatum* | Alkaloids | -5.59 | -4.91 | [38] |
| 528 | Pseudopelletierine |  | *Punica granatum* | Alkaloids | -4.94 | -3.92 | [38] |
| 529 | Norpseudopelletierine |  | *Punica granatum* | Alkaloids | -4.64 | -3.68 | [38] |
| 530 | Sedridine | s | *Punica granatum* | Alkaloids | -5.12 | -4.12 | [38] |
| 531 | Hygrine |  | *Punica granatum* | Alkaloids | -5.82 | -4.48 | [38] |
| 532 | NorHygrine |  | *Punica granatum* | Alkaloids | -5.05 | -3.86 | [38] |
| 533 | Brevifolin |  | *Punica granatum* | Tannin | -5.64 | -4.94 | [38] |
| 534 | Brevifolincarboxylic acid |  | *Punica granatum* | Tannin | -6.39 | -5.23 | [38] |
| 535 | Ethyl brevifolincarboxylate |  | *Punica granatum* | Tannin | -7.67 | -4.91 | [38] |
| 536 | 3,4,8,9,10-penta-hydroxydibenzo[b,d]pyran-6-one |  | *Punica granatum* | Tannin | -6.47 | -4.53 | [38] |
| 537 | Casuarinin |  | *Punica granatum* | Tannin | -9.12 | **-9.69** | [38] |
| 538 | corilagin |  | *Punica granatum* | Tannin | -6.54 | **-6.33** | [38] |
| 539 | ellagic acid |  | *Punica granatum* | Tannin | -6.33 | -5.20 | [38] |
| 540 | Gallic acid |  | *Punica granatum* | Tannin | -5.08 | -4.56 | [38] |
| 541 | Methyl gallate |  | *Punica granatum* | Tannin | -5.17 | -4.77 | [38] |
| 542 | 1,2,4,6-tetra-O-galloyl -B-D-glucose |  | *Punica granatum* | Tannin | -7.52 | **-9.41** | [38] |
| 543 | 1,4-di-O-galloyl-3,6-(R)-hexahydroxydiphenoyl-B-glucose |  | *Punica granatum* | Tannin | -6.91 | **-8.32** | [38] |
| 544 | 2-O-Galloylpunicalin |  | *Punica granatum* | Tannin | -7.42 | **-10.59** | [38] |
| 545 | 2,3-(S)-HHDP-D-glucose |  | *Punica granatum* | Tannin | -7.10 | **-6.66** | [38] |
| 546 | Granatin A |  | *Punica granatum* | Tannin | -6.60 | **-8.54** | [38] |
| 547 | Granatin B |  | *Punica granatum* | Tannin | -4.79 | **-9.23** | [38] |
| 548 | 3-O-methylellagic acid |  | *Punica granatum* | Tannin | -6.73 | -5.24 | [38] |
| 549 | 3,3’-di-O-methylellagic acid |  | *Punica granatum* | Tannin | -7.19 | -5.10 | [38] |
| 550 | 4,4-di-O-methylellagic acid |  | *Punica granatum* | Tannin | -7.22 | -5.42 | [38] |
| 551 | 3,3’,4’-tri-O-methylellagic acid |  | *Punica granatum* | Tannin | -7.44 | -5.84 | [38] |
| 552 | 3’-O-methyl-3,4-methylenedioxyellagic acid |  | *Punica granatum* | Tannin | -6.78 | -5.12 | [38] |
| 553 | Pedunculagin |  | *Punica granatum* | Tannin | -7.01 | **-8.65** | [38] |
| 554 | Punicacortein A |  | *Punica granatum* | Tannin | -6.89 | **-8.31** | [38] |
| 555 | Punicacortein B |  | *Punica granatum* | Tannin | -7.30 | **-7.48** | [38] |
| 556 | Punicacortein C |  | *Punica granatum* | Tannin | -7.04 | **-9.44** | [38] |
| 557 | Punicafolin |  | *Punica granatum* | Tannin | - | - | [38] |
| 558 | Punicalagin |  | *Punica granatum* | Tannin | -7.29 | **-11.06** | [38] |
| 559 | Punicalin |  | *Punica granatum* | Tannin | -5.44 | **-7.27** | [38] |
| 560 | Punigluconin |  | *Punica granatum* | Tannin | -6.95 | **-8.78** | [38] |
| 561 | Tellimagrandin I |  | *Punica granatum* | Tannin | -9.13 | **-9.33** | [38] |
| 562 | Punicacortein D |  | *Punica granatum* | Tannin | -5.52 | **-9.55** | [38] |
| 563 | 5-O-galloylpunicacortein D |  | *Punica granatum* | Tannin | -6.76 | **-8.98** | [38] |
| 564 | Apigenin |  | *Punica granatum* | Flavanoid | -7.10 | -5.39 | [38] |
| 565 | Apigenin 4’-O-glucopyranoside |  | *Punica granatum* | Flavanoid | -9.56 | **-7.49** | [38] |
| 566 | Catechin |  | *Punica granatum* | Flavanoid | -6.33 | -5.47 | [38] |
| 567 | Catechol |  | *Punica granatum* | Flavanoid | -5.16 | -3.83 | [38] |
| 568 | Cyanidin |  | *Punica granatum* | Flavanoid | -7.37 | -4.64 | [38] |
| 569 | Cyanidin 3-O-glucoside |  | *Punica granatum* | Flavanoid | -6.92 | **-6.56** | [38] |
| 570 | Cyanidin 3,5-di-O-glucosid |  | *Punica granatum* | Flavanoid | -9.44 | **-7.64** | [38] |
| 571 | Delphinidin 3-O-glucoside |  | *Punica granatum* | Flavanoid | -6.58 | **-6.78** | [38] |
| 572 | Delphinidin 3,5-di-O-glucoside |  | *Punica granatum* | Flavanoid | -10.14 | **-8.85** | [38] |
| 573 | Epicatechin |  | *Punica granatum* | Flavanoid | -6.77 | -5.50 | [38] |
| 574 | Epigallocatechin 3-gallate |  | *Punica granatum* | Flavanoid | -6.66 | **-6.76** | [38] |
| 575 | Flavan-3-ol |  | *Punica granatum* | Flavanoid | -6.87 | -4.69 | [38] |
| 576 | Isoquercetin |  | *Punica granatum* | Flavanoid | -7.65 | **-6.49** | [38] |
| 577 | Kaempferol |  | *Punica granatum* | Flavanoid | -7.22 | -5.05 | [38] |
| 578 | Kaempferol-3-O-glucoside/astragalin |  | *Punica granatum* | Flavanoid | -7.13 | **-6.56** | [38] |
| 579 | Kaempferol-3-O-rhamnoglycoside |  | *Punica granatum* | Flavanoid | -7.52 | **-7.59** | [38] |
| 580 | Luteolin |  | *Punica granatum* | Flavanoid | -7.12 | -5.10 | [38] |
| 581 | Luteolin 7-O-glucoside |  | *Punica granatum* | Flavanoid | -8.82 | **-6.83** | [38] |
| 582 | Luteolin 4’-O-glucopyranoside |  | *Punica granatum* | Flavanoid | -9.59 | **-6.71** | [38] |
| 583 | Luteolin 3’-O-glucopyranoside |  | *Punica granatum* | Flavanoid | -9.07 | **-6.52** | [38] |
| 584 | Naringin |  | *Punica granatum* | Flavanoid | -10.02 | **-7.19** | [38] |
| 585 | Pelargonidin |  | *Punica granatum* | Flavanoid | -7.43 | -5.51 | [38] |
| 586 | Pelargonidin 3-O-glucoside |  | *Punica granatum* | Flavanoid | -7.10 | **-7.02** | [38] |
| 587 | Pelargonidin 3,5-di-O-glucoside |  | *Punica granatum* | Flavanoid | -3.67 | **-7.96** | [38] |
| 588 | Procyanidin |  | *Punica granatum* | Flavanoid | -5.25 | **-8.41** | [38] |
| 589 | Prodelphinidin |  | *Punica granatum* | Flavanoid | -5.33 | **-8.05** | [38] |
| 590 | Quercetin |  | *Punica granatum* | Flavanoid | -7.62 | -5.39 | [38] |
| 591 | Rutin |  | *Punica granatum* | Flavanoid | **-**9.43 | **-8.09** | [38] |
| 592 | ursolic acid |  | *Punica granatum* | Triterpenoid | -2.28 | -6.18 | [38] |
| 593 | oleanolic acid |  | *Punica granatum* | Triterpenoid | -2.46 | **-6.27** | [38] |
| 594 | Maslinic acid |  | *Punica granatum* | Triterpenoid | -3.14 | **-6.37** | [38] |
| 595 | Punicanolic acid |  | *Punica granatum* | Triterpenoid | -2.81 | **-6.24** | [38] |
| 596 | friedelin |  | *Punica granatum* | Triterpenoid | -4.50 | **-6.38** | [38] |
| 597 | Betulinic acid |  | *Punica granatum* | Triterpenoid | -3.88 | **-6.56** | [38] |
| 598 | Asiatic acid |  | *Punica granatum* | Triterpenoid | -3.78 | -6.09 | [38] |
| 599 | coumestrol |  | *Punica granatum* | coumestans | -6.62 | -4.89 | [38] |
| 600 | phenethyl rutinoside |  | *Punica granatum* | Glycoside | -9.52 | **-7.47** | [38] |
| 601 | Reserpine |  | *Rauvolfia serpentina.* | Alkaloid | -8.46 | **-7.67** | [39] |
| 602 | Ajmaline |  | *Rauvolfia serpentina.* | Indole Alkaloid | -5.20 | -5.09 | [39] |
| 603 | Isoajmaline |  | *Rauvolfia serpentina.* | Indole alakloid | -4.81 | -5.27 | [39] |
| 604 | Ajmalicine |  | *Rauvolfia serpentin* | indole alkaloid | -6.80 | -5.62 | [39] |
| 605 | Yohimbine |  | *Rauvolfia serpentina* | Indole Alkaloid | -7.41 | -6.09 | [39] |
| 606 | Deserpidine |  | *Rauvolfia serpentina* | Indole Alkaloid | -9.36 | **-7.92** | [39] |
| 607 | Rescinnamine |  | *Rauvolfia serpentina* | Indole Alkaloid | -7.88 | **-8.36** | [39] |
| 608 | Serpentinine |  | *Rauvolfia serpentina* | Indole Alkaloid | -8.50 | **-8.51** | [39] |
| 609 | Corynanthine |  | *Rauvolfia serpentina* | Alkaloid | -7.01 | **-6.35** | [39] |
| 610 | Papaverine |  | *Rauvolfia serpentina* | Alkaloid | -7.85 | **-6.30** | [39] |
| 611 | Sarpagine |  | *Rauvolfia serpentina* | Indole Alkaloid | -6.44 | -5.31 | [39] |
| 612 | Serpentine |  | *Rauvolfia serpentina* | Indole Alkaloid | -7.30 | -5.25 | [39] |
| 613 | Alstonine |  | *Rauvolfia serpentina* | Alkaloid | -6.82 | -5.36 | [39] |
| 614 | Renoxidine |  | *Rauvolfia serpentina* | Indole Alkaloid | -8.18 | **-8.17** | [39] |
| 615 | Reserpiline |  | *Rauvolfia serpentina* | Alkaloid | -7.53 | **-7.56** | [39] |
| 616 | Ophioxylin |  | *Rauvolfia serpentina* | phenol | -6.31 | -4.05 | [39] |
| 617 | Rauwolscine |  | *Rauvolfia serpentina* | alkaloid | -7.05 | -5.59 | [39] |
| 618 | Thebaine |  | *Rauvolfia serpentina* | alakloid | -5.09 | -4.97 | [39] |
| 619 | 7-Dehydrositosterol |  | *Rauvolfia serpentina* | sterol | -6.78 | **-7.16** | [39] |
| 620 | Stigmasterol |  | *Rauvolfia serpentina* | steroid | -7.41 | **-6.75** | [39] |
| 621 | 2,6-Dimethoxybenzoquinone |  | *Rauvolfia serpentina* | Benzoquinone | -5.20 | -3.59 | [39] |
| 622 | Tetraphyllicine |  | *Rauvolfia serpentina* | Alkaloid | -5.54 | -5.15 | [39] |
| 623 | Raucaffricine |  | *Rauvolfia serpentina* | Alkaloid | -6.70 | **-7.23** | [39] |
| 624 | Vomilenine |  | *Rauvolfia serpentina* | alkaloid | -5.09 | -5.21 | [39] |
| 625 | Raumacline |  | *Rauvolfia serpentina* | Alkaloid | -5.97 | -5.06 | [39] |
| 626 | Raucaffrinoline |  | *Rauvolfia serpentina* | Alkaloid | -5.12 | -5.63 | [39] |
| 627 | Perakine |  | *Rauvolfia serpentina* | Alkaloid | -4.55 | -5.33 | [39] |
| 628 | Vinorine |  | *Rauvolfia serpentina* | Alkaloid | -6.12 | -5.65 | [39] |
| 629 | 16-epi-vellosimine |  | *Rauvolfia serpentina* | Alkaloid | -6.37 | -5.19 | [39] |
| 630 | 11-Methoxyvinorine |  | *Rauvolfia serpentina* | Alkaloid | -6.66 | -5.58 | [39] |
| 631 | Vellosimine |  | *Rauvolfia serpentina* | Alkaloid | -6.08 | -4.65 | [39] |
| 632 | 1,2-dihydrovomilenine |  | *Rauvolfia serpentina* | Alkaloid | -5.16 | -5.45 | [39] |
| 633 | 17-O-Acetyl-norajmaline |  | *Rauvolfia serpentina* | alkaloid | -4.21 | -5.67 | [39] |
| 634 | Norajmaline |  | *Rauvolfia serpentina* | Alkaloid | -5.77 | -5.44 | [39] |
| 635 | Rescinnamidine |  | *Rauvolfia serpentina* | Alkaloid | -9.00 | **-8.73** | [39] |
| 636 | Tetraphylline |  | *Rauvolfia serpentina* | Alkaloid | -7.64 | -6.21 | [39] |
| 637 | Indobine |  | *Rauvolfia serpentina* | Alkaloid | -7.96 | -5.55 | [39] |
| 638 | Indobinine |  | *Rauvolfia serpentina* | Alkaloid | -7.16 | -5.65 | [39] |
| 639 | Isorauhimbine |  | *Rauvolfia serpentina* | Alkaloid | -6.93 | -5.34 | [39] |
| 640 | Rauhimbine |  | *Rauvolfia serpentina* | Alkaloid | -7.00 | -5.47 | [39] |
| 641 | Sandwicoline |  | *Rauvolfia serpentina* | Alkaloid |  |  | [39] |
| 642 | Sandwicolidine |  | *Rauvolfia serpentina* | Alkaloid | - | - | [39] |
| 643 | Fumaric acid |  | *Rauvolfia serpentina* | dicarboxylic acids | -4.83 | -3.88 | [39] |
| 644 | GAMMA-Sitosterol |  | *Rauvolfia serpentina* | sterol | -6.87 | -6.03 | [39] |
| 645 | b-Sitosterol |  | *Rauvolfia serpentina* | sterol | -6.71 | **-6.42** | [39] |
| 646 | Diisobutylphthalate |  | *Rauvolfia serpentina* | phthalate ester | -8.51 | -4.82 | [39] |
| 647 | Vallesiachotamine |  | *Rauvolfia serpentina* | Alkaloid | -6.51 | **-6.30** | [39] |
| 648 | Yohimbinic acid |  | *Rauvolfia serpentina* | Alkaloid | -6.90 | -5.72 | [39] |
| 649 | 3-Hydroxysarpagine |  | *Rauvolfia serpentina* | Alkaloid | -6.36 | -5.83 | [39] |
| 650 | Isorauhimbinic acid |  | *Rauvolfia serpentina* | Alkaloid | -6.29 | -5.57 | [39] |
| 651 | 7-Epiloganin |  | *Rauvolfia serpentina* | Alkaloid | -7.16 | **-6.23** | [39] |
| 652 | Normacusine B |  | *Rauvolfia serpentina* | Alkaloid | -5.85 | -5.53 | [39] |
| 653 | 6’-O-(3,4,5-  trimethoxybenzoyl)glomeratose |  | *Rauvolfia serpentina* | Alkaloid | -10.16 | **-9.17** | [39] |
| 654 | Geissoschizol |  | *Rauvolfia serpentina* | Alkaloid | -6.81 | -5.32 | [39] |
| 655 | Rhazimanine |  | *Rauvolfia serpentina* | Alkaoid | -7.49 | **-6.06** | [39] |
| 656 | Methyl reserpate |  | *Rauvolfia serpentina* | Alkaloid | -7.72 | **-6.33** | [39] |
| 657 | 18-Hydroxyepialloyohimbine |  | *Rauvolfia serpentina* | Alkaloid | -6.45 | -5.72 | [39] |
| 658 | Loganic acid |  | *Rauvolfia serpentina* | iridoid monoterpeneglusoside | -7.38 | -5.79 | [39] |
| 659 | 7-Deoxyloganic acid |  | *Rauvolfia serpentina* | Monoterpenoid glycoside | -8.06 | -5.40 | [39] |
| 660 | Secoxyloganin |  | *Rauvolfia serpentina* | glycoside | -7.37 | -6.06 | [39] |
| 661 | Glomeratose A |  | *Rauvolfia serpentina* | sesquiterpenoid | -9.28 | **-7.77** | [39] |
| 662 | 16-Epinormacusine B |  | *Rauvolfia serpentina* | Alkaloid | -6.46 | -5.03 | [39] |
| 663 | Swertiaside |  | *Rauvolfia serpentina* | Alkaloid | -9.03 | **-6.91** | [39] |
| 664 | 3,4,5,6-Tetradehydroyohimbine |  | *Rauvolfia serpentina* | Alkaloid | -7.25 | -5.48 | [39] |
| 665 | 3,4,5,6-Tetradehydro-(Z)-geissoschizol |  | *Rauvolfia serpentina* | Alkaoid | -6.77 | -5.17 | [39] |
| 666 | 3,4,5,6-Tetradehydrogeissoschizol |  | *Rauvolfia serpentina* | Alkaloid | -8.54 | -6.19 | [39] |
| 667 | Arbutin |  | *Rauvolfia serpentina* | glycosylated hydroquinone | -7.35 | -4.89 | [39] |
| 668 | Ajmalimine |  | *Rauvolfia serpentina* | Alkaoid | -6.27 | **-7.01** | [39] |
| 669 | Tryptamine |  | *Rauvolfia serpentina* | Indole amine | -6.28 | -4.68 | [39] |
| 670 | Secologanin |  | *Rauvolfia serpentina* | Monoterpene | -8.23 | -6.14 | [39] |
| 671 | Nb-Methylraumacline |  | *Rauvolfia serpentina* | Alkaloid | -5.16 | **-6.26** | [39] |
| 672 | Tetrahydroalstonine |  | *Rauvolfia serpentina* | Alkaloid | -5.65 | -5.45 | [39] |
| 673 | 19(S),20(R)-dihydroperaksine |  | *Rauvolfia serpentina* | Alkaoid | -5.56 | -5.79 | [39] |
| 674 | 12-Hydroxyajmaline |  | *Rauvolfia serpentina* | Alakoid | -5.48 | -4.72 | [39] |
| 675 | 18-beta-hydroxy-3-epi-alpha-yohimbine |  | *Rauvolfia serpentina* | Alkaloid | -6.90 | -5.70 | [39] |
| 676 | Strictosidine |  | *Rauvolfia serpentina* | Alkaloid | -7.77 | **-7.54** | [39] |
| 677 | Strictosidine lactam |  | *Rauvolfia serpentina* | Alkaloid | -8.47 | **-7.34** | [39] |
| 678 | Eburnamonine |  | *Rauvolfia serpentina* | Alkaloid | -5.56 | -4.79 | [39] |
| 679 | Stemmadenine |  | *Rauvolfia serpentina* | Alkaloid | -4.52 | -5.48 | [39] |
| 680 | Akuammigine |  | *Rauvolfia serpentina* | Alkaloid | -6.68 | -5.54 | [39] |
| 681 | Gardnerine |  | *Rauvolfia serpentina* | Alkaloid | -7.24 | -5.54 | [39] |
| 682 | Isosandwicine |  | *Rauvolfia serpentina* | Alkaloid | -4.51 | -5.24 | [39] |
| 683 | Rauniticine |  | *Rauvolfia serpentina* | Alkaloid | -6.82 | -5.65 | [39] |
| 684 | Sandwicine |  | *Rauvolfia serpentina* | Alkaloid | - | - | [39] |
| 685 | Vincoside |  | *Rauvolfia serpentina* | Alkaloid | -8.41 | **-6.83** | [39] |
| 686 | Beta-yohimbine |  | *Rauvolfia serpentina* | Alkaloid | -7.41 | -6.16 | [39] |
| 687 | Tubotaiwine |  | *Rauvolfia serpentina* | Alkaloid | -5.73 | **-6.34** | [39] |
| 688 | Suaveoline |  | *Rauvolfia serpentina* | Alkaloid | -6.31 | -4.94 | [39] |
| 689 | Macrophylline |  | *Rauvolfia serpentina* | Alkaloid | -6.70 | -4.86 | [39] |
| 690 | Rhazinilam |  | *Rauvolfia serpentina* | Alkaloid | -5.01 | -4.86 | [39] |
| 691 | Raunescine |  | *Rauvolfia serpentina* | Alkaloid | -7.03 | **-7.42** | [39] |
| 692 | Ajmalicidine |  | *Rauvolfia serpentina* | Alkaloid | -6.94 | -5.55 | [39] |
| 693 | Ajmalinimine |  | *Rauvolfia serpentina* | Alkaloid | - | - | [39] |
| 694 | Dehydrogeissoschizine |  | *Rauvolfia serpentina* | Alkaloid | -7.81 | -6.07 | [39] |
| 695 | chlorogenic acid |  | *Coriandrum sativum* | Polyphenol(phenolic acids) | -7.97 | -6.10 | [45] |
| 696 | Gallic acid |  | *Coriandrum sativum* | Phenolic acid | -5.08 | -4.58 | [45] |
| 697 | Vanillic acid |  | *Coriandrum sativum* | Phenolic acid | -5.42 | -4.07 | [45] |
| 698 | Caffeic acid |  | *Coriandrum sativum* | Phenolic acid | -5.64 | -4.18 | [45] |
| 699 | P-Coumaric acid |  | *Coriandrum sativum* | Phenolic acid | -5.54 | -3.89 | [45] |
| 700 | Rosmarinic acid |  | *Coriandrum sativum* | Phenolic acid | -8.67 | -5.94 | [45] |
| 701 | Ferulic acid |  | *Coriandrum sativum* | Phenolic acid | -5.64 | -4.23 | [45] |
| 702 | O-coumaric acid |  | *Coriandrum sativum* | Phenolic acid | -4.89 | -4.10 | [45] |
| 703 | Salicylic acid |  | *Coriandrum sativum* | Phenolic acid | -4.80 | -4.29 | [45] |
| 704 | Trans-cinnamic acid |  | *Coriandrum sativum* | Polyphenol | -5.10 | -4.28 | [45] |
| 705 | Quercetin-3-rhamnoside |  | *Coriandrum sativum* | Flavanoids | -6.31 | -6.00 | [45] |
| 706 | Luteolin |  | *Coriandrum sativum* | Flavanoid | -7.12 | -5.10 | [45] |
| 707 | Rutin trihydrate |  | *Coriandrum sativum* | Flavanoid | - | - | [45] |
| 708 | Resorcinol |  | *Coriandrum sativum* | Flavanoid | -4.45 | -3.37 | [45] |
| 709 | Quercetin dihydrate |  | *Coriandrum sativum* | Flavanoid | - | - | [45] |
| 710 | Kaempferol |  | *Coriandrum sativum* | Flavanoid | -7.26 | -5.15 | [45] |
| 711 | Apigenin |  | *Coriandrum sativum* | Flavanoid | -7.05 | -5.38 | [45] |
| 712 | Naringin |  | *Coriandrum sativum* | Flavanoid | -10.00 | **-6.88** | [45] |
| 713 | Coumarin |  | *Coriandrum sativum* | Flavanoid | -4.58 | -4.11 | [45] |
| 714 | Flavone |  | *Coriandrum sativum* | Flavanoid | -6.63 | -5.13 | [45] |
| 715 | caffeoyl N-tryptophan hexoside |  | *Coriandrum sativum* | Phenol derivative | -8.76 | **-7.71** | [45] |
| 716 | N-Caffeoyltryptophan |  | *Coriandrum sativum* | Phenol derivative | -9.54 | -5.99 | [45] |
| 717 | 4,5-di-o-caffeoyl quinic acid |  | *Coriandrum sativum* | Phenol derivative | -7.46 | **-7.74** | [45] |
| 718 | β-carotene |  | *Coriandrum sativum* | Terpenoid | -8.65 | **-7.55** | [45] |
| 719 | (S)-(+)-linalool |  | *Coriandrum sativum* | Monoterpene | -5.71 | -4.23 | [45] |
| 720 | P-mentha-1,4-dien-7-ol |  | *Coriandrum sativum* | Monoterpenoid | -5.32 | -4.06 | [45] |
| 721 | Neryl acetate |  | *Coriandrum sativum* | Monoterpenoid | -6.81 | -5.24 | [45] |
| 722 | α-pinene |  | *Coriandrum sativum* | Monoterpene | -4.90 | -3.95 | [45] |
| 723 | γ-terpinene |  | *Coriandrum sativum* | Monoterpene | -4.86 | -4.46 | [45] |
| 724 | Geranyl acetate |  | *Coriandrum sativum* | Monoterpenoid | -6.67 | -5.01 | [45] |
| 725 | Anethole |  | *Coriandrum sativum* | phenylpropanoid | -5.48 | -4.62 | [45] |
| 726 | P-cymene |  | *Coriandrum sativum* | Monoterpene | -4.78 | -4.50 | [45] |
| 727 | Camphor |  | *Coriandrum sativum* | Monoterpene | -4.74 | -3.49 | [45] |
| 728 | Phellandrene |  | *Coriandrum sativum* | Monoterpene | -5.16 | -4.72 | [45] |
| 729 | Linalyl acetate |  | *Coriandrum sativum* | Monoterpenoid | -6.06 | -4.840 | [45] |
| 730 | Limonene |  | *Coriandrum sativum* | Monoterpene | -5.30 | -4.45 | [45] |
| 731 | Geraniol |  | *Coriandrum sativum* | Monoterpenoid | -5.88 | -4.97 | [45] |
| 732 | β-Sitosterol |  | *Coriandrum sativum* | Phytosterol | -6.68 | **-7.49** | [45] |
| 733 | Stigmasterol |  | *Coriandrum sativum* | Steroid | -6.42 | **-6.79** | [45] |
| 734 | Δ7-stigmasterol |  | *Coriandrum sativum* | Steroid | -5.78 | -6.53 | [45] |
| 735 | Campesterol |  | *Coriandrum sativum* | Phytosterol | -7.64 | **-8.04** | [45] |
| 736 | Δ5-avenasterol |  | *Coriandrum sativum* | Phytosterol | -6.95 | **-6.79** | [45] |
| 737 | Δ7-avenasterol |  | *Coriandrum sativum* | Phytosterol | -7.12 | **-6.93** | [45] |
| 738 | Lanosterol |  | *Coriandrum sativum* | Triterpenoid | -5.54 | **-6.44** | [45] |
| 739 | Ergosterol |  | *Coriandrum sativum* | Phytosterol | -6.90 | **-7.44** | [45] |
| 740 | α-tocopherol |  | *Coriandrum sativum* | Tocopherol | -7.93 | **-7.49** | [45] |
| 741 | δ-tocopherol |  | *Coriandrum sativum* | Tocopherol | -7.74 | **-6.94** | [45] |
| 742 | γ-tocopherol |  | *Coriandrum sativum* | Tocopherol | -7.98 | **-7.15** | [45] |
| 743 | γ-tocotrienol |  | *Coriandrum sativum* | Tocotrienol | -9.07 | -**7.22** | [45] |
| 744 | α-Tocotrienol |  | *Coriandrum sativum* | Tocotrienol | -8.30 | **-7.68** | [45] |
| 745 | δ-tocotrienol |  | *Coriandrum sativum* | Tocotrienol | -9.40 | **-7.48** | [45] |
| 746 | Quercetin-3-O-rutinoside |  | *Coriandrum sativum* | Polyphenol | -6.89 | **-8.23** | [45] |
| 747 | Quercetin 3-O-glucuronide |  | *Coriandrum sativum* | Polyphenol | -6.29 | **-6.73** | [45] |
| 748 | Quercetin-3-O-glucoside |  | *Coriandrum sativum* | Polyphenol | -6.91 | **-7.17** | [45] |
| 749 | Kaempferol-3-rutinoside |  | *Coriandrum sativum* | Polyphenol | **-**7.58 | **-8.44** | [45] |
| 750 | P-coumaroylquinic acid |  | *Coriandrum sativum* | Flavanoids | -7.59 | -5.85 | [45] |
| 751 | Ferulic acid glucoside |  | *Coriandrum sativum* | Flavanoid | -7.78 | -5.66 | [45] |
| 752 | Hyperoside |  | *Coriandrum sativum* | Flavanoid | -7.10 | **-6.53** | [45] |
| 753 | Vicenin-2 |  | *Coriandrum sativum* | Flavanoid | **-**7.32 | **-7.37** | [45] |
| 754 | Hesperidin |  | *Coriandrum sativum* | Flavanoid | -8.82 | **-8.14** | [45] |
| 755 | Orientine |  | *Coriandrum sativum* | Flavanoid | **-**7.41 | **-6.21** | [45] |
| 756 | Diosmin |  | *Coriandrum sativum* | Flavanoid | -8.71 | **-8.12** | [45] |
| 757 | Taxifolin |  | *Coriandrum sativum* | Flavanoid | -6.81 | -4.92 | [45] |
| 758 | Catechin |  | *Coriandrum sativum* | Flavanoid | -6.35 | -5.48 | [45] |
| 759 | Chrysoeriol |  | *Coriandrum sativum* | Flavanoid | -7.82 | -5.78 | [45] |
| 760 | Dicoumarin |  | *Coriandrum sativum* | Hydroxycoumarin | -8.05 | -5.25 | [45] |
| 761 | Esculin |  | *Coriandrum sativum* | Hydroxycoumarin | -7.99 | -5.38 | [45] |
| 762 | 4-hydroxycoumarin |  | *Coriandrum sativum* | Hydroxycoumarin | -5.44 | -3.94 | [45] |
| 763 | Esculetin |  | *Coriandrum sativum* | Hydroxycoumarin | -5.29 | -4.27 | [45] |
| 764 | Arbutin |  | *Coriandrum sativum* | Phenolic glycoside | -7.35 | -5.03 | [45] |
| 765 | Tartaric acid |  | *Coriandrum sativum* | Organic acid | -4.80 | -3.69 | [45] |
| 766 | Cis-Ferulic acid |  | *Coriandrum sativum* | Phenolic acid | -5.37 | -4.85 | [45] |
| 767 | Vanillic acids |  | *Coriandrum sativum* | Phenolic acid | -5.42 | -4.32 | [45] |
| 768 | Acacetin |  | *Coriandrum sativum* | Flavanoid | -7.43 | -5.86 | [45] |
| 769 | Quercetin |  | *Coriandrum sativum* | Flavanoid | -7.63 | -5.38 | [45] |
| 770 | 4'-O-Methyl Quercetin |  | *Coriandrum sativum* | Flavanoid | -7.82 | -5.01 | [45] |
| 771 | 3'-O-Methylquercetin |  | *Coriandrum sativum* | Flavanoid | -7.47 | -5.37 | [45] |
| 772 | Protocatechuic acid |  | *Coriandrum sativum* | Phenolic acid | -4.88 | -3.71 | [45] |
| 773 | Maleic acid |  | *Coriandrum sativum* | Dicarboxylic acid | -4.83 | -3.89 | [45] |
| 774 | Glycitin |  | *Coriandrum sativum* | Flavanoid | -8.82 | **-7.05** | [45] |
| 775 | Anthocyanin |  | *Coriandrum sativum* | Flavanoid | -6.72 | -5.05 | [45] |
| 776 | Camphene |  | *Coriandrum sativum* | Monoterpene | -4.57 | -3.93 | [40] |
| 777 | Δ-3-carene |  | *Coriandrum sativum* | Monoterpene | -4.68 | -4.49 | [40] |
| 778 | Myrcene |  | *Coriandrum sativum* | Monoterpene | -5.34 | -4.53 | [40] |
| 779 | Trans-ocimene |  | *Coriandrum sativum* | Monoterpene | -5.21 | -4.58 | [40] |
| 780 | Cis-Ocimene |  | *Coriandrum sativum* | Monoterpene | -5.29 | -4.80 | [40] |
| 781 | B-phellandrene |  | *Coriandrum sativum* | Monoterpene | -5.17 | -4.37 | [40] |
| 782 | α-terpinene |  | *Coriandrum sativum* | Monoterpene | -5.14 | -4.64 | [40] |
| 783 | β-pinene |  | *Coriandrum sativum* | Monoterpene | -4.75 | -4.01 | [40] |
| 784 | Sabinene |  | *Coriandrum sativum* | Monoterpene | -4.71 | -4.26 | [40] |
| 785 | Terpinolene |  | *Coriandrum sativum* | Monoterpene | -5.14 | -4.44 | [40] |
| 786 | α-thujene |  | *Coriandrum sativum* | Monoterpene | -4.94 | -4.15 | [40] |
| 787 | 1,8-ciol(Eucalyptol) |  | *Coriandrum sativum* | Monoterpenoid | -4.87 | -4.06 | [40] |
| 788 | Linalol oxide |  | *Coriandrum sativum* | Monoterpenoid | -6.02 | -4.35 | [40] |
| 789 | Carvone |  | *Coriandrum sativum* | Monoterpenoid | -4.74 | -4.26 | [40] |
| 790 | Geranial |  | *Coriandrum sativum* | Monoterpenoid | -5.33 | -4.60 | [40] |
| 791 | Borneol |  | *Coriandrum sativum* | monoterpenoid | -4.40 | -3.68 | [40] |
| 792 | Citronellol |  | *Coriandrum sativum* | Monoterpenoid | -6.23 | -4.22 | [40] |
| 793 | Nerol |  | *Coriandrum sativum* | Monoterpenoid | -5.56 | -4.60 | [40] |
| 794 | α-terpineol |  | *Coriandrum sativum* | Monoterpenoid | -5.25 | -4.10 | [40] |
| 795 | 4-terpinenol |  | *Coriandrum sativum* | monoterpenoid | -5.50 | -4.32 | [40] |
| 796 | Bornyl acetate |  | *Coriandrum sativum* | Monoterpenoid | -5.03 | -4.39 | [40] |
| 797 | α-terpinyl acetate |  | *Coriandrum sativum* | Monoterpenoid | -5.76 | -5.08 | [40] |
| 798 | β-Caryophyllene |  | *Coriandrum sativum* | Sesquiterpenes | -4.93 | -4.98 | [40] |
| 799 | Caryophyllene oxide |  | *Coriandrum sativum* | Sesquiterpenes | -5.01 | -4.60 | [40] |
| 800 | Elemol |  | *Coriandrum sativum* | Sesquiterpenes | -4.61 | -4.95 | [40] |
| 801 | Nerolidol |  | *Coriandrum sativum* | Sesquiterpenes | -6.59 | -5.56 | [40] |
| 802 | Myristicin |  | *Coriandrum sativum* | Phenol | -5.50 | -4.61 | [40] |
| 803 | Thymol |  | *Coriandrum sativum* | Phenol | -5.04 | -4.70 | [40] |
| 804 | Norboreneolacetate |  | *Coriandrum sativum* | Monoterpenoids | -4.79 | -4.40 | [40] |
| 805 | Citronellal |  | *Coriandrum sativum* | Monoterpenoid | -5.39 | -4.63 | [40] |
| 806 | α-cedrene |  | *Coriandrum sativum* | Sesquiterpenoid | -4.26 | -4.35 | [40] |
| 807 | α- farnesene |  | *Coriandrum sativum* | Sesquiterpenoids | -6.30 | -5.50 | [40] |
| 808 | β-Sesquiphellandrene |  | *Coriandrum sativum* | Sesquiterpenoids | -6.87 | -5.54 | [40] |
| 809 | E-Verbenol |  | *Coriandrum sativum* | Monoterpenoid | -4.93 | -3.78 | [40] |
| 810 | Umbellulone |  | *Coriandrum sativum* | Monoterpenoid | -5.20 | -4.36 | [40] |
| 811 | Isothujol |  | *Coriandrum sativum* | Azoles | -5.35 | -4.03 | [40] |
| 812 | α-Bisabolol |  | *Coriandrum sativum* | Sesquiterpenoid | -6.91 | -5.01 | [40] |
| 813 | β-Bisabolene |  | *Coriandrum sativum* | Sesquiterpenoid | -6.11 | -5.07 | [40] |
| 814 | Curcumene |  | *Coriandrum sativum* | Sesquiterpenoid | -6.56 | -5.04 | [40] |
| 815 | Coriandrone A |  | *Coriandrum sativum* | 2-benzopyran | -6.68 | -5.43 | [40] |
| 816 | Coriandrone B |  | *Coriandrum sativum* | 2-benzopyran | -6.63 | -5.49 | [40] |
| 817 | Coriandrone C |  | *Coriandrum sativum* | Isocoumarins | -6.52 | -4.75 | [40] |
| 818 | Coriandrone D |  | *Coriandrum sativum* | 2-benzopyran | -7.66 | -5.48 | [40] |
| 819 | Coriandrone E |  | *Coriandrum sativum* | 2-benzopyran | -5.96 | -4.90 | [40] |
| 820 | Coriandrin |  | *Coriandrum sativum* | Isocoumarins | -6.17 | -4.80 | [46] |

**References**

33. Dubey T, Chinnathambi S. Brahmi (Bacopa monnieri): An ayurvedic herb against the Alzheimer’s disease. Arch Biochem Biophys. 2019;676: 108153. doi:10.1016/j.abb.2019.108153

34. Bag A, Bhattacharyya SK, Chattopadhyay RR. The development of Terminalia chebula (Combretaceae) in clinical research. Asian Pac J Trop Biomed. 2013;3: 244–252. doi:10.1016/S2221-1691(13)60059-3

35. Saleem S, Muhammad G, Hussain MA, Altaf M, Bukhari SNA. Withania somnifera L.: Insights into the phytochemical profile, therapeutic potential, clinical trials, and future prospective. Iran J Basic Med Sci. 2020;23: 1501–1526. doi:10.22038/IJBMS.2020.44254.10378

36. Kunjumon R, Johnson AJ, Baby S. Centella asiatica: Secondary metabolites, biological activities and biomass sources. Phytomedicine Plus. 2022;2: 100176. doi:10.1016/j.phyplu.2021.100176

37. Variya BC, Bakrania AK, Patel SS. Emblica officinalis (Amla): A review for its phytochemistry, ethnomedicinal uses and medicinal potentials with respect to molecular mechanisms. Pharmacol Res. 2016;111: 180–200. doi:10.1016/j.phrs.2016.06.013

38. Wang R-F, Ding Y, Liu X, Du L. Pomegranate: Constituents, Bioactivities and Pharmacokinetics. Fruit Veg Cereal Sci Biotechnol. 2010;4.

39. Pathania S, Randhawa V, Bagler G. Prospecting for Novel Plant-Derived Molecules of Rauvolfia serpentina as Inhibitors of Aldose Reductase, a Potent Drug Target for Diabetes and Its Complications. Zheng J, editor. PLoS One. 2013;8: e61327. doi:10.1371/journal.pone.0061327

40. Al-Snafi PDAE. A review on chemical constituents and pharmacological activities of Coriandrum sativum. IOSR J Pharm. 2016;06: 17–42. doi:10.9790/3013-067031742

41. Bishayee A, Patel PA, Sharma P, Thoutireddy S, Das N. Lotus (Nelumbo nucifera .) and Its Bioactive Phytocompounds: A Tribute to Cancer Prevention and Intervention. Cancers (Basel). 2022;14: 529. doi:10.3390/cancers14030529

42. Riaz M, Khan O, Sherkheli MA KM and RR. Chemical Constituents of Terminalia chebula. Nat Prod Ind J. 2017;13: 112.

43. Azerad R. Chemical structures, production and enzymatic transformations of sapogenins and saponins from Centella asiatica (L.) Urban. Fitoterapia. 2016;114: 168–187. doi:10.1016/j.fitote.2016.07.011

44. CHONG NJ AZ. A systematic review on the chemical constituents of Centella asiatica. Res J Pharm Biol Chem Sci. 2011;2: 445.

45. Iqbal MJ, Butt MS, Suleria HAR. Coriander (Coriandrum sativum L.): Bioactive Molecules and Health Effects. 2018. pp. 1–37. doi:10.1007/978-3-319-54528-8_44-1

46. Nimish LP, Sanjay BK, Nayna MB, Jaimik DR. Phytopharmacological properties of coriander sativum as a potential medicinal tree: An overview. J Appl Pharm Sci. 2011;1: 20–25.

47. Sharma V, Sharma R, Gautam D, Kuca K, Nepovimova E, Martins N. Role of Vacha (Acorus calamus .) in Neurological and Metabolic Disorders: Evidence from Ethnopharmacology, Phytochemistry, Pharmacology and Clinical Study. J Clin Med. 2020;9: 1176. doi:10.3390/jcm9041176

48. Jeyasri R, Muthuramalingam P, Suba V, Ramesh M, Chen J-T. Bacopa monnieri and Their Bioactive Compounds Inferred Multi-Target Treatment Strategy for Neurological Diseases: A Cheminformatics and System Pharmacology Approach. Biomolecules. 2020;10: 536. doi:10.3390/biom10040536

49. Remya C, Dileep K V., Variayr EJ, Sadasivan C. An in silico guided identification of nAChR agonists from Withania somnifera. Front Life Sci. 2016;9: 201–213. doi:10.1080/21553769.2016.1207569
