# Supplementary material for: Computer-aided discovery of dual-target compounds for Alzheimer’s from ayurvedic medicinal plants
Source: PLoS One. 2025 Jun 25;20(6):e0325441. doi: 10.1371/journal.pone.0325441 (PMC12193798; doi:10.1371/journal.pone.0325441)
Supplement: S2 Table — (DOCX) [file pone.0325441.s017.docx]

**S2 Table** Ligand Interaction report of dual inhibitors of human acetylcholinesterase (AChE, PDB: 6O4W) and β-secretase (BACE1, PDB: 6EJ3).

| **Compound** | **Target** | **Ligand**  **Groups** | **Receptor**  **Groups** | **Interaction**  **Type** | **Distance**  **(Å)** | **Energy**  **(kcal/mol)** |
| --- | --- | --- | --- | --- | --- | --- |
| Standard | AChE | N 4 | O HOH 708 | H-donor | 2.93 | -5.2 |
|  |  | O 1 | N PHE 295 | H-acceptor | 3.14 | -3.6 |
|  |  | C 6 | 6-ring TYR 341 | H-pi | 3.58 | -0.6 |
|  |  | C 11 | 6-ring TYR 337 | H-pi | 3.66 | -0.8 |
|  |  | 6-ring | 6-ring TRP 86 | pi-pi | 3.88 | -0.0 |
| Standard | BACE-1 | S 1 | O GLY 34 | H-donor | 3.10 | -0.8 |
|  |  | N 8 | O GLY 34 | H-donor | 3.01 | -1.2 |
|  |  | N 11 | O PHE 108 | H-donor | 3.25 | -2.0 |
|  |  | O 5 | NH2 ARG 128 | H-acceptor | 2.80 | -4.1 |
| Compound 2 | AChE | O 12 | OD2 ASP 74 | H-donor | 2.68 | -1.2 |
|  |  | O 18 | OE2 GLU 202 | H-donor | 2.91 | -1.4 |
|  |  | O 19 | OE1 GLU 202 | H-donor | 2.96 | -3.6 |
|  | BACE-1 | O 2 | OG SER 325 | H-donor | 2.70 | -8.6 |
|  |  | O 7 | O PHE 108 | H-donor | 2.76 | -2.0 |
|  |  | O 15 | OD2 ASP 32 | H-donor | 3.27 | -2.3 |
|  |  | O 19 | O ILE 126 | H-donor | 2.92 | -1.5 |
|  |  | O 2 | NE ARG 235 | H-acceptor | 3.33 | -1.2 |
|  |  | O 19 | N SER 36 | H-acceptor | 3.09 | -2.1 |
| Compound 3 | AChE | O 8 | O HOH 875 | H-acceptor | 2.74 | -1.4 |
|  |  | C 25 | 6-ring TRP 286 | H-pi | 3.86 | -0.6 |
|  |  | O 31 | 5-ring TRP 286 | H-pi | 3.85 | -0.6 |
|  | BACE-1 | O 7 | NE1 TRP 76 | H-acceptor | 3.12 | -1.7 |
|  |  | O 62 | NE ARG 235 | H-acceptor | 3.01 | -1.5 |
| Compound 4 | AChE | O 14 | OD2 ASP 74 | H-donor | 2.67 | -2.2 |
|  |  | O 15 | N PHE 295 | H-acceptor | 2.80 | -3.2 |
|  |  | O 18 | 6-ring PHE 338 | H-pi | 3.54 | -1.0 |
|  |  | C 56 | 6-ring TRP 286 | H-pi | 4.16 | -0.6 |
|  | BACE-1 | O 15 | OD2 ASP 228 | H-donor | 3.22 | -1.0 |
|  |  | O 7 | N THR 232 | H-acceptor | 3.37 | -0.6 |
|  |  | O 14 | OG1 THR 72 | H-acceptor | 2.65 | -1.4 |
| Compound 5 | AChE | O 14 | OD2 ASP 74 | H-donor | 2.52 | -0.6 |
|  |  | C 66 | 6-ring TYR 337 | H-pi | 4.00 | -0.6 |
|  |  | O 18 | NZ LYS 224 | H-acceptor | 2.89 | -5.5 |
|  | BACE-1 | O 9 | O GLY 230 | H-donor | 3.12 | -0.7 |
|  |  | O 2 | NZ LYS 321 | H-acceptor | 2.99 | -3.7 |
| Compound 6 | AChE | O 43 | OD2 ASP 74 | H-donor | 2.36 | 3.0 |
|  |  | C 8 | 5-ring TRP 86 | H-pi | 3.78 | -1.6 |
|  |  | C 8 | 6-ring TRP 86 | H-pi | 4.70 | -0.6 |
|  |  | C 27 | 5-ring TRP 286 | H-pi | 3.87 | -1.0 |
|  |  | 6-ring | 6-ring TRP 86 | pi-pi | 3.84 | -0.0 |
|  | BACE-1 | O 37 | OD2 ASP 228 | H-donor | 2.75 | -3.1 |
|  |  | O 38 | OD2 ASP 228 | H-donor | 2.91 | -4.1 |
|  |  | O 28 | NH1 ARG 128 | H-acceptor | 3.49 | -0.8 |
|  |  | 6-ring | NE1 TRP 76 | pi-H | 3.81 | -1.0 |
| Compound 7 | AChE | O 9 | O SER 293 | H-donor | 2.87 | -2.1 |
|  |  | O 10 | O SER 293 | H-donor | 2.86 | -2.1 |
|  |  | O 16 | N PHE 295 | H-acceptor | 3.16 | -1.4 |
|  |  | O 13 | 5-ring TRP 286 | H-pi | 3.91 | -0.9 |
|  | BACE-1 | O 2 | OD1 ASN 233 | H-donor | 2.79 | -0.9 |
|  |  | O 8 | OD2 ASP 32 | H-donor | 3.12 | -2.2 |
|  |  | O 9 | OD1 ASP 32 | H-donor | 3.00 | -0.9 |
|  |  | O 10 | OD2 ASP 228 | H-donor | 2.53 | -2.8 |
|  |  | O 1 | NZ LYS 321 | H-acceptor | 2.71 | -4.2 |
|  |  | O 15 | N THR 72 | H-acceptor | 3.28 | -0.6 |
|  |  | O 20 | NH1 ARG 128 | H-acceptor | 3.36 | -1.0 |
| Compound 8 | AChE | C 44 | O TYR 341 | H-donor | 3.26 | -0.8 |
|  |  | O 10 | O HOH 832 | H-acceptor | 2.45 | 0.5 |
|  |  | C 51 | 6-ring TYR 341 | H-pi | 3.75 | -0.7 |
|  | BACE-1 | O 2 | N ASN 37 | H-acceptor | 3.30 | -0.8 |
|  |  | O 9 | N ASN 233 | H-acceptor | 3.20 | -1.6 |
|  |  | O 10 | N THR 232 | H-acceptor | 3.02 | -2.0 |
| Compound 9 | AChE | O 10 | O HOH 788 | H-donor | 2.94 | -0.7 |
|  |  | O 16 | OD2 ASP 74 | H-donor | 2.65 | -2.1 |
|  |  | O 15 | N PHE 295 | H-acceptor | 3.17 | -0.8 |
|  |  | C 63 | 6-ring TYR 341 | H-pi | 3.61 | -0.9 |
|  |  | C 67 | 6-ring TYR 337 | H-pi | 4.16 | -0.9 |
|  |  | O 19 | 6-ring TYR 337 | H-pi | 3.87 | -0.7 |
|  | BACE-1 | O 2 | NH1 ARG 307 | H-acceptor | 3.15 | -2.2 |
|  |  | O 16 | N THR 72 | H-acceptor | 3.49 | -0.5 |
|  |  | O 19 | NE1 TRP 76 | H-acceptor | 3.16 | -1.7 |
| Compound 10 | AChE | O 9 | O HOH 797 | H-donor | 2.61 | 0.6 |
|  |  | O 16 | O HOH 708 | H-donor | 3.00 | -0.8 |
|  |  | O 19 | O HIS 447 | H-donor | 2.71 | -1.9 |
|  |  | O 20 | OE1 GLU 202 | H-donor | 3.08 | -1.6 |
|  |  | O 2 | NE2 HIS 287 | H-acceptor | 3.14 | -0.6 |
|  | BACE-1 | O 10 | OD2 ASP 32 | H-donor | 2.97 | -3.0 |
|  |  | O 18 | OD2 ASP 228 | H-donor | 2.80 | -2.6 |
|  |  | O 19 | OD2 ASP 228 | H-donor | 3.00 | -2.6 |
|  |  | C 56 | 6-ring TYR 71 | H-pi | 3.84 | -0.6 |
| Compound 11 | AChE | O 9 | O HOH 711 | H-donor | 2.94 | -1.3 |
|  |  | O 10 | O SER 293 | H-donor | 2.51 | -1.3 |
|  |  | O 16 | OD2 ASP 74 | H-donor | 2.53 | -0.3 |
|  | BACE-1 | O 77 | O LEU 263 | H-donor | 3.09 | -1.1 |
|  |  | O 15 | NH1 ARG 128 | H-acceptor | 2.80 | -1.3 |
| Compound 12 | AChE | O 15 | OD2 ASP 74 | H-donor | 2.63 | -1.8 |
|  |  | O 17 | O HOH 875 | H-acceptor | 2.91 | -0.7 |
|  | BACE-1 | O 12 | OD2 ASP 228 | H-donor | 2.98 | -3.6 |
|  |  | O 14 | OD2 ASP 228 | H-donor | 3.11 | -2.2 |
| Compound 13 | AChE | O 18 | O HOH 756 | H-donor | 3.00 | -0.4 |
|  |  | O 20 | OE1 GLU 202 | H-donor | 3.26 | -1.1 |
|  |  | C 59 | 6-ring TYR 337 | H-pi | 4.11 | -0.8 |
|  | BACE-1 | O 9 | O PHE 108 | H-donor | 2.90 | -2.3 |
|  |  | O 1 | NZ LYS 224 | H-acceptor | 3.30 | -2.9 |
|  |  | O 18 | NH2 ARG 307 | H-acceptor | 3.24 | -1.3 |
|  |  | O 19 | NH1 ARG 307 | H-acceptor | 3.18 | -2.1 |
|  |  | O 19 | NH2 ARG 307 | H-acceptor | 3.39 | -0.4 |
| Compound 14 | AChE | O 8 | O SER 293 | H-donor | 2.89 | -0.8 |
|  |  | O 14 | OD2 ASP 74 | H-donor | 2.58 | -1.3 |
|  |  | O 2 | O HOH 771 | H-acceptor | 2.83 | -1.4 |
|  |  | O 15 | N PHE 295 | H-acceptor | 3.19 | -1.2 |
|  |  | O 17 | O HOH 875 | H-acceptor | 2.48 | -0.2 |
|  | BACE-1 | O 1 | OG1 THR 232 | H-acceptor | 2.78 | -0.9 |
|  |  | O 7 | NE1 TRP 76 | H-acceptor | 2.79 | -2.5 |
|  |  | O 10 | NH1 ARG 128 | H-acceptor | 2.80 | -0.8 |
|  |  | O 12 | NH2 ARG 128 | H-acceptor | 2.87 | -4.1 |
| Compound 15 | AChE | O 9 | O HOH 756 | H-donor | 3.20 | -0.7 |
|  |  | O 11 | OE1 GLU 202 | H-donor | 2.86 | -3.7 |
|  |  | C 31 | 5-ring TRP 286 | H-pi | 4.14 | -0.6 |
|  |  | 6-ring | 6-ring TRP 86 | pi-pi | 3.97 | -0.0 |
|  | BACE-1 | / | / | / | / | / |
